# Shape selective bifacial recognition of double helical DNA

**DOI:** 10.1038/s42004-018-0080-5

**Published:** 2018-11-07

**Authors:** Shivaji A. Thadke, V.M. Hridya, J. Dinithi R. Perera, Roberto R. Gil, Arnab Mukherjee, Danith H. Ly

**Affiliations:** 1 Department of Chemistry and Institute for Biomolecular Design and Discovery (IBD), Carnegie Mellon University, 4400 Fifth Avenue, Pittsburgh, PA 15213, USA.; 2 Department of Chemistry, Indian Institute of Science Education and Research (IISER), Pune, Maharashtra 411008, India.

## Abstract

An impressive array of antigene approaches has been developed for recognition of double helical DNA over the past three decades; however, few have exploited the ‘Watson–Crick’ base-pairing rules for establishing sequence-specific recognition. One approach employs peptide nucleic acid as a molecular reagent and strand invasion as a binding mode. However, even with integration of the latest conformationally-preorganized backbone design, such an approach is generally confined to sub-physiological conditions due to the lack of binding energy. Here we report the use of a class of shape-selective, bifacial nucleic acid recognition elements, namely Janus bases, for targeting double helical DNA or RNA. Binding occurs in a highly sequence-specific manner under physiologically relevant conditions. The work may provide a foundation for the design of oligonucleotides for targeting the secondary and tertiary structures of nucleic acid biopolymers.

It has been over six decades since the structure of double helical DNA was first unveiled^1^. To chemists and biologists working in the field of molecular recognition at the time, the structure provided more than just insight into the mechanism for the storage and transmission of genetic information. It provided a paradigm for recognition of genetic materials, in a way in which the two opposing strands are held together: through hydrogen-bonding interaction between the adenine (A) nucleobase and thymine (T), and between cytosine (C) and guanine (G). These so called ‘Watson-Crick’ base-pairing rules are routinely employed in the design of oligonucleotide molecules for targeting single-stranded or perturbed regions of double-stranded DNA or RNA; however, they are rarely applied in the recognition of double helical B-form DNA (B-DNA)—the most stable form of DNA double helix. The reason is that most oligonucleotide molecules developed to date do not have sufficient binding energy to invade B-DNA. To circumvent this challenge, past research efforts have generally been focused on the minor and major grooves, in large part, because of the ease of accessibility of the chemical groups and the precedence for their recognition in Nature^2,3^. This pursuit has led to the development of several major classes of antigene molecules including polyamides^4^, triplex-forming oligonucleotides^5^, zinc-finger peptides^6,7^, and transcription activator-like effectors (TALEs)^8,9^. Notwithstanding considerable advances in these areas, the issues of sequence selection and/or specificity, to a certain extent, still remain with many of these classes of molecules. Recently, a CRISPR-Cas9 system has been harnessed from bacteria for the manipulation of genomes and has achieved remarkable success^10,11^. Even with this state-of-the-art technology, though, an improvement in sequence-specificity is warranted for many of the anticipated biomedical applications due to the concern for off-target genome modifications^12,13^.

In concert with these efforts, Nielsen and coworkers^14,15^ have shown over the past two decades that peptide nucleic acid (PNA), a nucleic acid mimic comprising a pseudopeptide backbone (Fig. 1a), can invade B-DNA. Strand invasion of DNA by PNA occurs predominantly through Watson-Crick^16^, or in combination with Hoogsteen base-pairing^17^, depending on the sequence context. The simplicity and the generality in sequence design, along with its exquisite recognition-specificity, make PNA an attractive antigene agent. However, with the original (achiral) backbone design, PNA recognition is restricted to mostly homopurine and homopyrimidine targets. Mixed-sequence PNA generally does not have sufficient binding energy to invade B-DNA. Progress has been made to relax the sequence constraint with the application of tail-clamp^18,19^ and double-duplex invasion strategies^20^, but they are not without limitations^21^. More recently we showed that PNA, when preorganized into a right-handed helical motif by installing an (*R*)-stereogenic center at the γ-backbone (Fig. 1b), can invade B-DNA without sequence limitation (Fig. 1c)^22^. This newly endowed property of PNA has been exploited in a number of biological applications, including diagnostics^23–25^ and gene editing^26^. Despite this recent advance, it remains a challenge to invade B-DNA at elevated, physiologically relevant ionic strengths. With increasing ionic strengths, particularly in the presence of divalent metal cations, such as Mg^2+^, the DNA double helix becomes significantly thermodynamically more stable. As such, additional binding energy would be required to invade B-DNA, a major hurdle facing the design of oligonucleotide molecules for targeting mixed-sequence double helical B-DNA (or RNA). It should be noted that our work is focused on targeting double stranded DNA in vitro and not in its native state in a cell, which is epigenetically modified, wrapped around histones, and bound to other proteins.

The required binding energy, in principle, could be attained by replacing natural nucleobases with synthetic analogs^27^, such as the replacements of adenine with 2,6-diaminopurine^28^ and cytosine with G-clamp^29^, with improved hydrogen-bonding and base-stacking interactions. However, such an approach could compromise recognition-specificity due to the propensity of the resultant ultra-high affinity probes to bind to DNA (or RNA) with closely related sequences, in addition to their intended binding sites. The challenge is in how to design oligonucleotide molecules that can invade B-DNA under physiologically relevant conditions without compromising sequence-specificity.

Here we report the use of a class of shape-selective, bifacial nucleic acid recognition elements for targeting double helical DNA with improvements in binding energy and sequence-specificity, enabling an effective strand invasion of double helical B-DNA, as well as RNA, under a physiologically simulated condition.

## Results

### Design rationale of bifacial recognition.

**Fig. 1:**
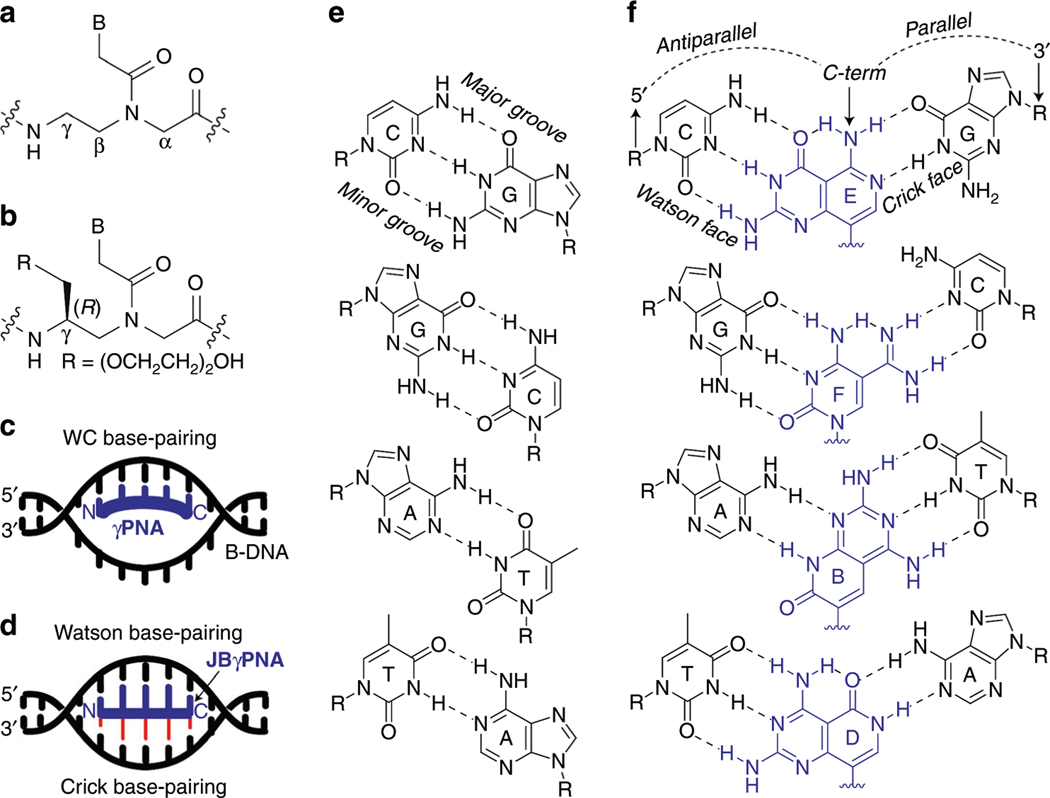
Chemical structures and binding modes of γPNAs. Structure of **(a)** PNA and **(b)** γPNA, **(c)** binding mode of γPNA containing natural nucleobases, and **(d)** that containing JBs. **e** Hydrogen-bonding interactions of natural bases, and **(f)** that of JBs with the canonical base-pairs

To augment the binding energy of γPNA, while simultaneously improving its recognition specificity, we envisioned the application of Janus bases (or JBs) as recognition elements since they have the potential to form hydrogen-bonds with nucleobases in both strands of the DNA double helix (Fig. 1d). We expected their interactions with double helical DNA to be more favorable than that of natural nucleobases (Fig. 1e), as a result of a significant increase in the number of hydrogen-bonding and in the degree of base-stacking interactions of the resulting triplex as compared to that of a duplex (Fig. 1f). An improvement in base-stacking interactions was anticipated due to the expanded aromatic ringsystems of JBs and as a result of the formation of base-triads. For every two hydrogen-bonds in an A–T or T–A pair, and for every three in a C–G or G–C that are broken, five new ones are formed upon the invasion of DNA by JBγPNA. Moreover, we expected their binding to be more sequence-specific because a single base-mismatch that would normally occur on one face of a natural nucleobase would occur on both faces of a JB. The concept of bifacial nucleic acid recognition, however, is not new. It was conceived by Lehn^30^ more than two decades ago and, subsequently, expounded upon by others in the development of Janus-wedges^31–38^. Despite concerted efforts from several research groups, only a small set of Janus-wedges has been developed, and they vary considerably in shapes and sizes, such that they cannot be effectively combined in a modular format for recognition of a non-homogenous nucleic acid sequence. Examples include those developed by Chen and McLaughlin^31,32^, which contained single-aromatic ring systems. As such, not only are they degenerate in recognition, unable to distinguish a C–G from G–C pair or an A–T from T–A, they neither possess the necessary binding energy to invade B-DNA nor a means to suppress self-hybridization. The latter is an intrinsic property of JBs, one that presents a major challenge in the design of bifacial nucleic acid recognition elements for targeting (canonical) double helical DNA or RNA. In contrast, the JBs under development are novel in structures and enabling in recognition capabilities. They were strategically designed and optimized so that they contain the appropriate shapes, sizes, chemical functionalities, and tautomeric structures for proper recognition of the respective DNA base-pairs (Fig. 1f), in addition to the asymmetry in the helically-folded γPNA backbone that enables them to hybridize to the designated base-pairs in double helical DNA or RNA, but not to each other.

### Molecular dynamics simulations.

**Fig. 2:**
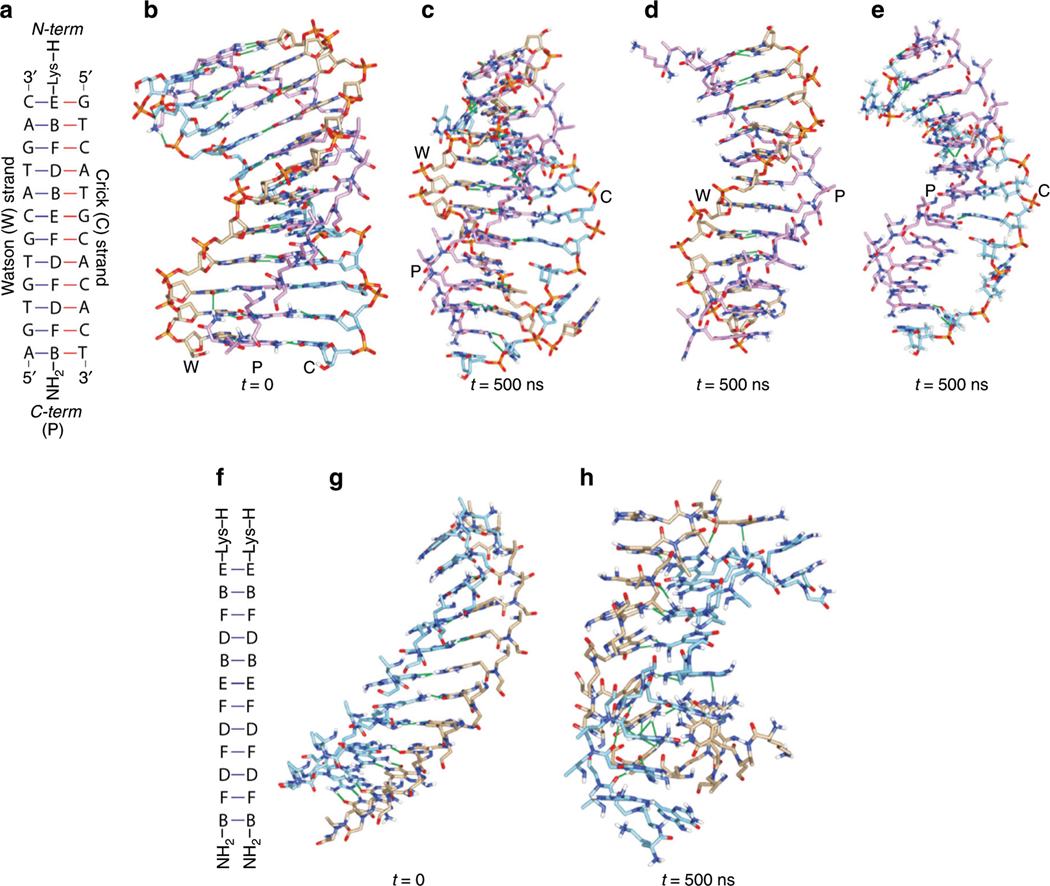
MD simulations of the triplex and duplex structures. **a–e** DNA-JBγPNA-DNA triplex, and **(f–h)** JBγPNA-JBγPNA duplex. **a** Sequence of triplex, **(b)** initial structure of triplex, **(c)** simulated structure of triplex after 500 ns, and **(d)** and **(e)** are the same as **(c)** but with the respective C and W strands removed for clarification. **f** Sequence of parallel duplex, **(g)** initial structure of duplex, and **(h)** simulated structure of duplex after 500 ns

Molecular dynamics (MD) simulations were initially performed to assess the feasibility of JB recognition (Supplementary Table 1). A dodecameric γPNA containing a mixture of all four JBs, H-Lys-EBFDBEFDFDFB-NH_2_, was chosen as a model system for computational modeling (Fig. 2a). This extended sequence was selected to ensure its proper binding with the Watson strand, as well as the Crick strand of DNA; and should it fail to hybridize to each other, this occurs as a result of steric clash in the backbone and not due to the short sequence. The four C-E-G, G-F-C, A-B-T, and T-D-A base-triads were built and optimized by the HF/6–31G* basis set and grafted onto the respective DNA and γPNA backbone (Supplementary Fig. 1)^39^. The structure of DNA-JBγPNA-DNA was created using the NAB module of Ambertools^40^ (Fig. 2b and Supplementary Data 1) and that of JBγPNA-JBγPNA was adopted from an existing NMR structure (Fig. 2f, g, and Supplementary Data 2)^41^. The result showed that the W-P portion of the triplex was stable after 500 ns (Fig. 2c and Supplementary Movie 1), remained intact throughout the simulations (Fig. 2d), while that of the P-C segment displayed a significant structural distortion (Fig. 2e). This is reflected in the number of hydrogen-bonds and in the inter-strand interaction energy (Supplementary Figs. 2–4). The weaker interaction of P-C, as compared to that of W-P, is attributed to the number of hydrogen-bonds being fewer and to the fact that hybridization occurs in a less favorable parallel orientation^42^. In contrast, as the result of steric clash in the backbone, the structure of JBγPNA-JBγPNA unraveled upon restraint release (Fig. 2h, Supplementary Fig. 5 and Supplementary Movie 2). Self-hybridization was a major concern in the design of JBγPNA, or any bifacial nucleic acid recognition system for targeting the canonical base-pairs of DNA or RNA for that matter, due to the complementarity of the two faces of JBs. However, we conjectured that such an event is less likely to occur with an asymmetrical, right-handed helically-induced chiral γPNA than with the achiral counterpart^39,43^. Indeed, the simulations revealed that not only can JBγPNA hybridize to both strands of the DNA double helix, but that it is unable to hybridize to each other—a prerequisite for a successful design of the bifacial nucleic acid system.

### Chemical synthesis and probe design.

**Fig. 3:**
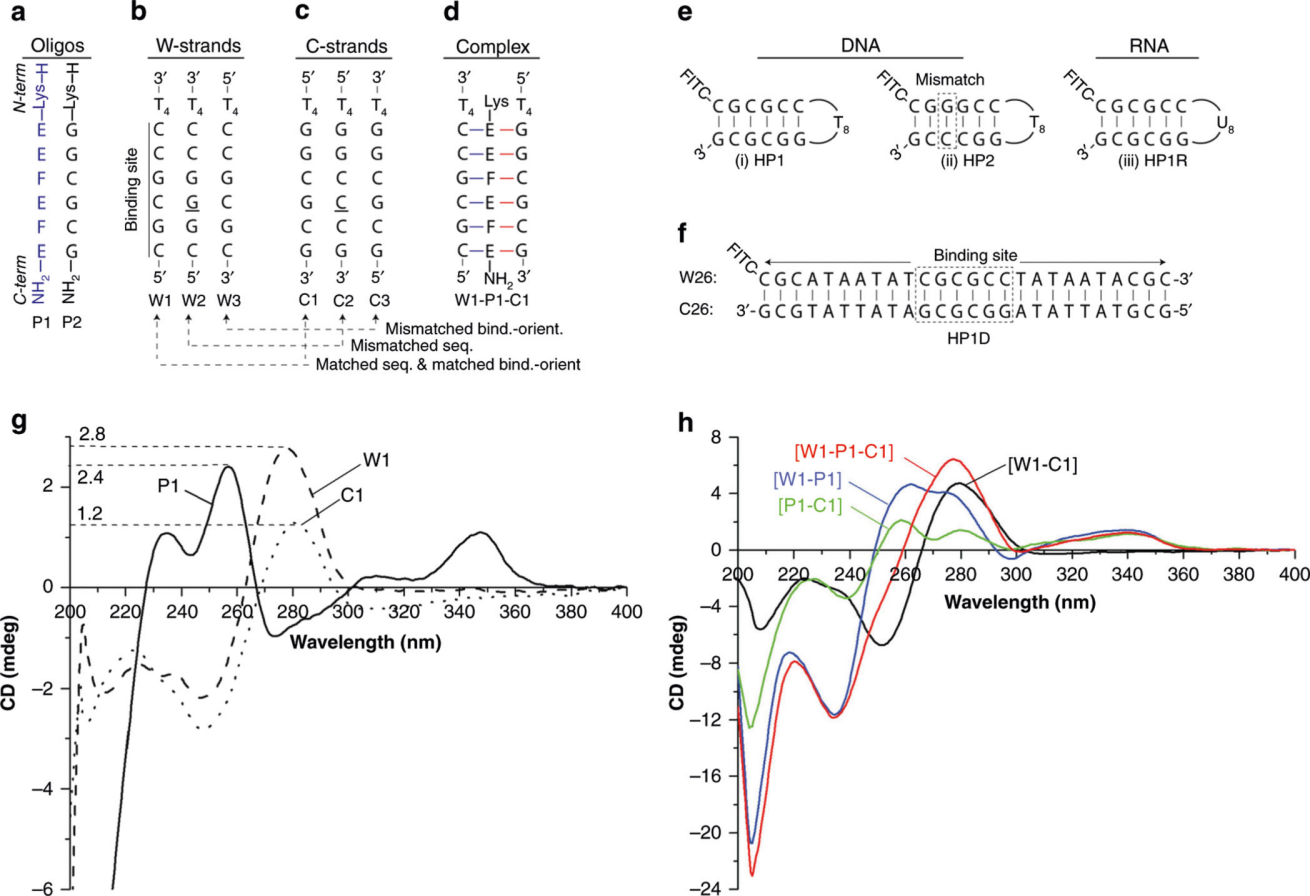
Probes and nucleic acid targets employed in this study along with their CD spectra. **a** Sequence of P1 (blue) and P2 (black), **(b)** Watson strands (W1, W2, W3), **(c)** Crick strands (C1, C2, C3), **(d)** depiction of a bound W1-P1-C1 triplex, **(e)** intramolecular DNA (HP1 and HP2) and RNA (HP1R) targets, **(f)**intermolecular DNA target (HP1D), **(g)** CD spectra of the individual strands (P1: solid line, W1: dashed line, C1: dotted line), and **(h)** CD spectra of W1-P1-C1 (red line), W1-P1 (blue line), W1-C1 (black line), and P1-C1 (green line) complexes. In **g** and **h** the concentration of each strand was 2.5 μM, prepared in a 1× PBS buffer (137 mM NaCl, 2.7 mM KCl, 10 mM NaPi, pH 7.4) and recorded at 25 °C

Encouraged by the results of MD simulations, we synthesized nucleobases E and F^44^, along with the corresponding γPNA monomers (Supplementary Fig. 6) and oligomer, for the initial testing (Fig. 3a and Supplementary Figs. 7 and 8). They were chosen based on the presumption that, if JBγPNA containing such recognition elements is able to invade B-DNA with the corresponding sequence, then those that contain B and D, along with their mixture with E and F, should be able to accomplish the same task but more effectively, since G-C and C-G pairs are thermodynamically more stable and, thus, more difficult to invade than A-T and T-A pairs. We selected a relatively short JBγPNA (P1), six units in length, for the initial proof-of-concept study because MD simulations suggest that this length is sufficient for binding DNA (or RNA) (Fig. 3a). P2, a homolog of P1 containing natural nucleobases, was also prepared for comparison. Binding studies were conducted with model DNA targets containing matching sequence and binding-orientation (W1 and C1, Fig. 3b, c), as well as those with mismatched sequence (W2 and C2) and mismatched binding orientation (W3 and C3). Figure 3d shows the preferential binding orientation of the triplex. Double-stranded DNA and RNA were also prepared (Fig. 3e, f), and their bindings with P1 and P2 were assessed using electrophoretic mobility-shift assays (EMSA). The difference between the two perfectly-matched DNA targets, HP1 (Fig. 3e, i) and HP1D (Fig. 3f), is that the former is an intramolecular hairpin and the latter is an intermolecular duplex—designed to assess the recognition generality of P1.

### Conformational analysis.

Circular dichroism (CD) was employed to assess the conformation of P1, along with those of the bound complexes. Consistent with the previous finding^43^, P1 adopted a right-handed helical motif, as evident by the biphasic exciton coupling pattern with maxima at 235, 255, and 340 nm and minima at 210 and 275 nm (Fig. 3g). We ruled out the possibility for self-hybridization on the basis of concentration-dependent measurements (Supplementary Fig. 9), along with other supporting evidence as discussed in the sections below. The W1-C1 mixture showed modest differential in CD signals. On the other hand, notable differences in the signal strengths and spectral patterns were observed with W1-P1, P1-C1, and W1-P1-C1, in comparison to that of the sum of individual strands (Fig. 3h and Supplementary Fig. 10)—suggesting that their interactions are more favorable than that of W1-C1. Such drastic changes in the CD signals, however, were not observed with the mismatched sequence (W2 and C2) or the mismatched binding-orientation (W3 and C3) (Supplementary Fig. 11). Collectively, these results provide supporting evidence for the binding of P1 with the Watson strand, as well as the Crick strand of DNA double helix in a sequence- and orientation-specific manner.

### Thermal and thermodynamic analyses.

**Fig. 4:**
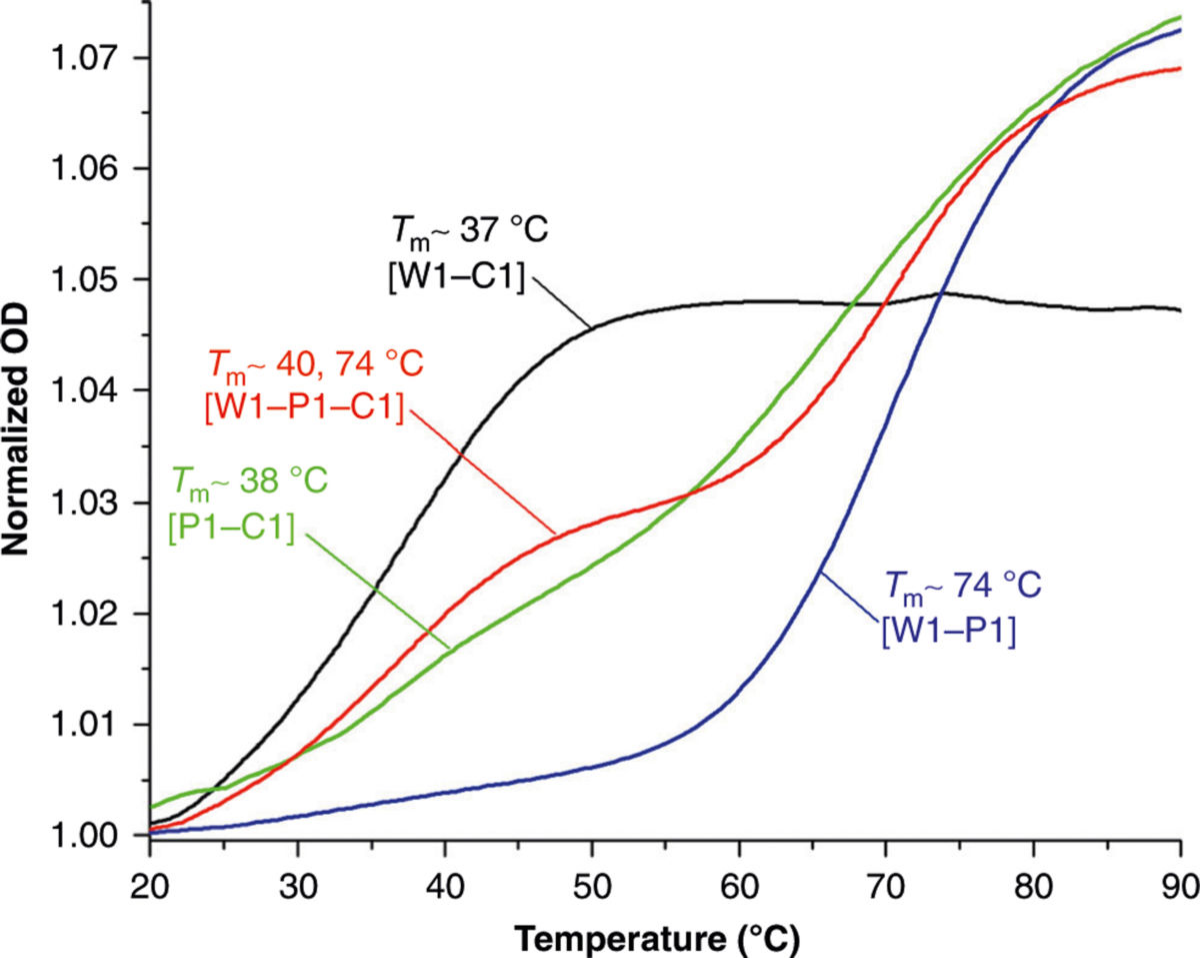
Thermal stability of the bound complexes. UV-melting profiles of W1-P1-C1 (red line), W1-P1 (blue line), W1-C1 (black line), and P1-C1 (green line). The concentration of each strand was 2.5 μM, prepared in a 1× PBS buffer

UV-melting experiment confirmed the formation of a W1-C1 duplex, although relatively weak, with a melting transition (T_m_) of ~37 °C (Fig. 4). However, under identical conditions, no discernible T_m_ was observed for P1-C1, suggesting that either the duplex did not form or that it did, but its melting profile merged with that of P1, which exhibited a broad melting pattern with T_m_ in the 35–80 °C range (Supplementary Fig. 12). The former scenario is unlikely based on the CD finding. The unusually high and broad T_m_ of P1 could be attributed to the increase in base-stacking interactions of E and F, in comparison to that of the natural nucleobases. A mixture of W1, P1, and C1 at an equimolar ratio displayed two well-defined melting transitions at 40 and 74 °C (Fig. 4). We assigned the first transition to the melting of P1-C1 and the second to that of W1-P1. W1-P1 and W1-P2 showed similar T_m_s, 74 °C and 72 °C, respectively (Supplementary Fig. 13). This result was expected since they both possess the same number of hydrogen-bonds. The thermodynamic parameters for W1-C1, W1-P1, and W1-P2 duplexes, as determined by van’t Hoff analyses, are shown in Table 1. In contrast to the observations made with W1 and C1, samples containing P1 and mismatched sequence (W2 and C2, Supplementary Fig. 14) or mismatched binding-orientation DNA (W3 and C3, Supplementary Fig. 15) did not yield well-defined melting patterns. These results are consistent with the CD findings, indicating that P1 has strong binding sequence and orientation preferences.

### Confirmation of binding by NMR.

**Fig. 5:**
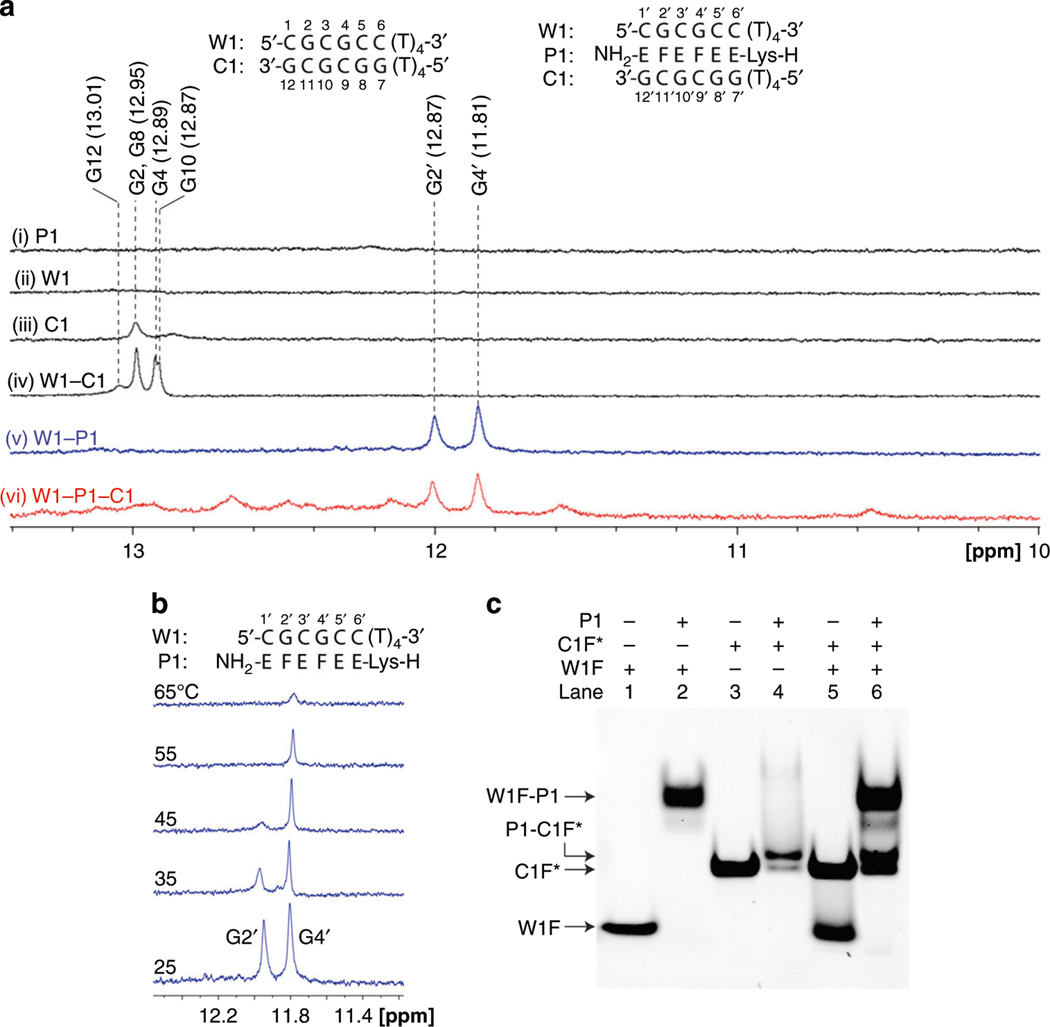
Imino proton signals of the bound complexes. **a** ^1^H-NMR spectra of the indicated samples in the 10.0–13.4 ppm range (no signals were observed beyond 13.4 ppm). Note P1, W1, C1, W1-C1: black line; W1-P1: blue line; W1-C1-P1: red line. **b** Imino proton signals of a W1-P1 duplex as a function of temperature. **c** Result of an EMSA showing the binding of various partners. Experimental conditions: pre-annealed DNA (W1F or C1F*) was mixed with P1 in a PR buffer (137 mM NaCl, 2.7 mM KCl, 10 mM NaPi, 2 mM MgCl_2_, pH 7.4) and incubated at 37 °C for 4 h; the resulting mixtures were separated by 10% non-denaturing PAGE. The sequences of FITC-attached DNA are as followed: W1F, 5’-CGCGCC-3’; and C1F*, 5’-(T)_12_GGCGCG-3’. The poly-(T)_12_ tail in C1F* was employed to provide separation between the two target strands. The strand concentrations of W1F, C1F*, and P1 were 1 μM each

To further substantiate the binding of P1 with W1 and C1, we carried out 1D and 2D NMR experiments at variable temperature and concentration. Figure 5a shows ^1^H-NMR spectra of the various combinations, prepared in a PBS buffer containing H_2_O:D_2_O at a 9:1 ratio; and those of the individual strands are shown in Supplementary Figs. 16–18. Several key observations were noted. First, the NMR spectrum of P1 was devoid of any imino proton signals in the 10.0–20.0 ppm region (Fig. 5a, i), indicating that self-hybridization did not take place even at such a high concentration (500 μM strand concentration). Second, C1 showed two imino proton signals at 12.95 and 12.80 ppm (Fig. 5a, iii, and Supplementary Fig. 19), as a result of the formation of a partial C1-C1 duplex stabilized by a terminal GG-diad. Self-hybridization did not occur with W1 because it lacked the terminal purine-cap (Fig. 5a, ii). Third, the titration of P1 with W1-C1 resulted in a gradual disappearance of the imino proton signals of G12, G2/G8, G10, and G4 (Fig. 5a, compare vi to iii and iv), concomitant with the formation of that of G2’ and G4’ of W1-P1 and other imino protons of P1-C1 (Fig. 5a, compare vi to v, and Supplementary Figs. 20 and 21; the other controls are shown in Supplementary Figs. 22–27). COSY and NOESY experiments enabled the assignment of imino protons of W1-C1 (Supplementary Figs. 28–33 and Supplementary Table 2) and W1-P1 (Supplementary Figs. 34–39). Fourth, W1-P1 complex appeared to be highly stable, as judging from the line sharpness and the persistence of imino proton signals at a temperature as high as 65 °C (Fig. 5b and Supplementary Figs. 40 and 41). While it is not currently feasible to assign the imino proton signals of E and F due to significant spectral overlaps with that of DNA nucleobases, a cursory inspection of W1-P1-C1 complex (Supplementary Fig. 42) confirms the binding of P1 with W1 and C1, as noted from the emergence of the aromatic W1-P1 proton signals (asterisk) and from the disappearance of that of C1 (filled circle). This suggestion was further corroborated by findings from EMSA (Fig. 5c) and MALDI-TOF MS (Supplementary Fig. 43) experiments, showing W1-P1 duplex being the most stable (compare lane 2 to lane 1), followed by P1-C1 (compare lane 4 to lane 3) and W1-C1 (compare lane 5 to lanes 1 and 3).

### Assessment of DNA strand invasion.

**Fig. 6:**
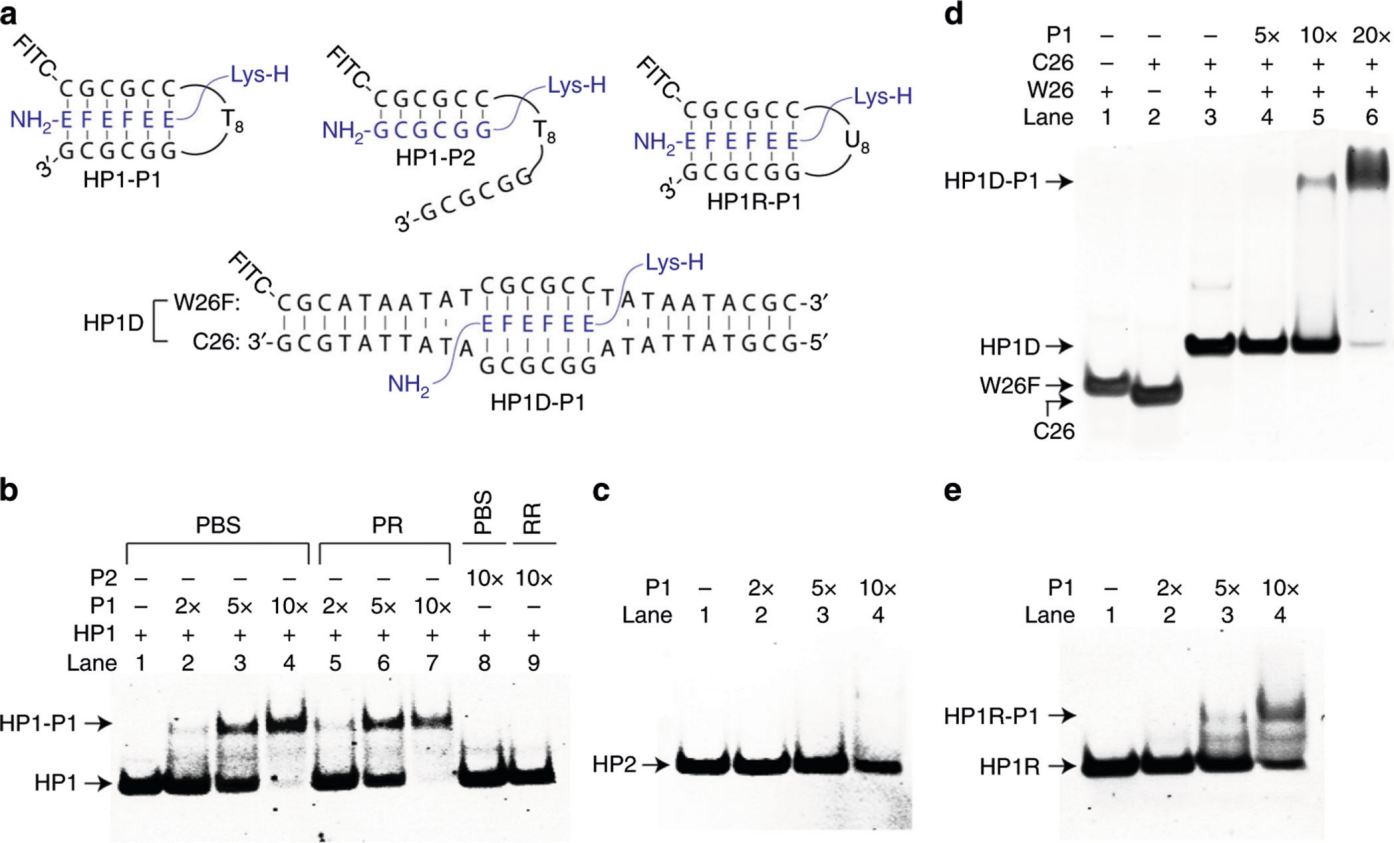
Invasion of double-stranded DNA and RNA by P1 and P2. **a** Compositions of the various complexes (HP1-P1, HP1-P2, HP1R-P1, HP1D-P1). **b** Comparison of binding of P1 (lanes 2–7) and P2 (lanes 8 and 9) with the perfectly-matched DNA hairpin (HP1) at a moderate (1× PBS) (lanes 1–4, and 8) and at a physiologically relevant (PR) ionic strength (lanes 5–7, and 9). The concentration of HP1 was 1 μM and that of P1 and P2 are as shown. The samples were prepared in the indicated buffers and incubated at 37 °C for 4 h prior to separation by non-denaturing PAGE. **c** Result of EMSA of P1 with mismatched HP2, **(d)** with HP1D, and **(e)** with HP1R RNA. In **c–e,** the samples were prepared in a PR buffer and incubated at the same temperature and duration, and separated under identical conditions as that in **b.** PR buffer: 137 mM NaCl, 2.7 mM KCl, 10 mM NaPi, 2 mM MgCl_2_, pH 7.4

To determine whether JBγPNA can invade a double helical B-DNA, we carried out EMSA comparing the binding of P1 and P2 with model DNA targets containing a perfectly-matched HP1 (Fig. 3e, i) and a base-pair inversion HP2 (Fig. 3e, ii) at a moderate ionic strength (1xPBS buffer), as well as at a physiologically relevant (PR) condition^45^. Figure 6a depicts compositions of the various binding complexes. The result demonstrated that not only can P1 invade a highly stable HP1 in PBS (Fig. 6b, lanes 2–4), but also in a PR buffer (lanes 5–7). Formation of the HP1-P1 invasion complex was confirmed by MALDI-TOF MS (Supplementary Fig. 44). The UV-melting profiles of the individual duplexes are shown in Supplementary Fig. 45. Binding occurred in a concentration-dependent manner, resulting in a complex that remained intact throughout the electrophoresis—as inferred from the sharpness of the shifted bands. Binding was relatively fast, complete within 10 min at 37 °C (Supplementary Fig. 46). In contrast, no evidence of binding was observed with P2, not even in a PBS buffer (compare lanes 8 and 9 to lanes 4 and 7, respectively). The fact that P1 and P2 have similar binding energy with the Watson strand (Table 1), but that only P1 was able to invade HP1 in PBS, as well as in a PR buffer, highlights the significant contribution of hydrogen-bonding interactions and, perhaps, steric clashes with the Crick strand of DNA double helix in stabilization of the invasion complex. No binding was observed with the mismatched HP2 (Fig. 6c). In addition to the hairpin structure, P1 was also able to target an internal binding site embedded within a highly stable double helix (Supplementary Fig. 45), as demonstrated with HP1D (Fig. 3f and Fig. 6d). The result showed that being able to simultaneously target both strands of the DNA double helix is essential for the invasion of B-DNA at a physiologically relevant ionic strength. A similar observation was made by Nielsen^20^ in the invasion of DNA by pseudo-complementary PNAs, *albeit* strand invasion requires simultaneous binding of two separate strands of PNA. Such a design, however, has practical limitations due to the slow hybridization kinetics and low invasion efficiency^21^.

### Strand invasion of RNA.

Interestingly, we noticed that P1 was also able to invade RNA double helix, HP1R (Fig. 3e, iii), in a PR buffer (Fig. 6e), where P2 was not. This result is intriguing, suggesting the possibility for targeting the secondary and tertiary structures of RNA. Such RNA structural motifs are highly abundant in cells and are known to play key roles in many biological processes^46^. Generally, they are challenging to target with conventional antisense oligonucleotides^47,48^ or with smallmolecule ligands with high selectivity^49,50^, although some progress has been made with the development and approval of nusinersen^51^ and patisiran drugs^52^, and in the application of oligonucleotides for targeting RNA-repeated expansions^53^.

## Discussion

The A–T (or A–U) and C–G base-pairing interactions are employed by Nature in the storage and transmission of genetic information, and by researchers in the recognition of genetic materials because of their exquisite recognition sequence-specificity. However, such principles have rarely been applied in the recognition of double helical DNA or RNA because most oligonucleotide molecules developed to date do not have sufficient binding energy to invade such a canonical duplex structure. The present study is focused on determining whether a bifacial JBγPNA could be designed, and, if so, whether it could invade a double helical DNA (or RNA) at a physiologically relevant ionic strength. A critical requirement for the successful development of such a dual-recognition nucleic acid system is its preferential binding with DNA (or RNA) but not with itself. We showed that not only can JBγPNA be prepared, but that it can form hydrogen-bonds with nucleobases in both strands of the DNA double helix without any evidence for self-hybridization. The helically-induced chirality in the backbone prevents JBγPNA from approaching and properly hybridizing to each other. In addition to its tight binding with the Watson strand, and to a lesser extent with the Crick strand, JBγPNA can invade double helical B-DNA, as well as RNA, under a simulated physiological condition.

A significant structural perturbation in the P-C segment of triplex, as revealed by MD simulations, was surprising but not unexpected. This is because the number of H-bonds formed in P-C (12 H-bonds) is fewer than that in W-P (18 H-bonds), and binding occurs in an unfavorable parallel orientation. This result was substantiated by experimental findings, as demonstrated by CD, UV-melting, NMR, and EMSA measurements. The fact that both P1 and P2 form the same number of H-bond with the Watson strand and that the resulting duplexes exhibited virtually identical thermodynamic stability, but P1 was able to invade B-DNA whereas P2 was not, indicates the importance of being able to engage the Crick strand in stabilization of the invasion complex. It is well established that DNA double helix is relatively dynamic, with the deoxyribose phosphate backbones and the nucleobases in constant flux with continual bending and twisting motions^54^. The reason that most oligonucleotide molecules are unable to productively invade B-DNA is not due to the lack of base-pair accessibility, but rather due to their inability to compete with the complementary DNA strand, especially in the context of a relatively long double helix^27^. In addition to the binding energy gained in the formation of P-C, although relatively small compared to the overall contribution, their interactions may provide a physical barrier in preventing the complementary DNA strand from re-hybridizing with its partner. Such a phenomenon has been illustrated by Nielsen and coworkers^20^.

While this proof-of-concept study is focused on the synthesis and evaluation of the binding property of a relatively short probe, the binding energy that would be required to invade a biologically relevant genomic DNA target, estimated to be ~18-nt in length, could be attained by expanding the probe length accordingly. This is because a γPNA-DNA duplex is thermodynamically more stable than DNA-DNA on a per-unit basis, as demonstrated in a previous study.^55^ We expected the thermodynamic stability of JBγPNA-DNA to be even greater than that of γPNA-DNA. If necessary, additional binding energy could be attained by employing second-generation JBs, such as those shown in Supplementary Fig. 47a, with improved hydrogen-bonding and basestacking interactions. For instance, E* and F** are capable of forming six hydrogen-bonds each with the respective C–G and G–C base-pairs, as compared to five each for E and F. Although F** is not the most stable isomer, previous studies showed that tautomers interconvert on a relatively fast time scale, and that rare occurrences can occur if they aid in the folding and stabilization of nucleic acid structures^56^.

Another potential benefit of a bifacial nucleic system is that it could be designed to bind more favorably with a double-stranded over a single-stranded DNA (or RNA) by furnishing it with a protective companion (Supplementary Fig. 47b). The application of toehold and strand displacement reaction to tune the binding kinetics and thermodynamics of nucleic acids—for the purpose of controlling their binding selectivity—has already been demonstrated^57^. Since binding occurs on both sides, a relatively short companion strand would be suffice to provide protection against single-stranded DNA (or RNA) binding, and maintain the required kinetics and thermodynamics for a successful invasion of double helical DNA (or RNA)^58^. Besides its protective role, the companion strand could be functionalized with a specific chemical group, such as guanidine, for improving cellular uptake^59^, and possibly for enhancing the rate of DNA (or RNA) strand invasion^60^. A distinctive advantage of such a molecular feature is that once it is displaced, upon a successful invasion of DNA (or RNA) by the designer probe, the companion strand is quenched through self-hybridization. Such a probe design may provide a safer and more effective means for targeting double-stranded DNA (or RNA) than the conventional antisense or antigene approach for biomedical applications.

In addition to the four JBs shown in Fig. 1f, which are designed to bind to the canonical base-pairs, the remaining twelve JBs within this class that are designed to bind to non-canonical base-pairs^44^ could be employed in combination to target the secondary and tertiary structures of RNA, in attempts to elucidate their physiological functions and to develop therapeutic interventions for treating genetic disorders. However, this does not imply that conventional antisense molecules, those containing natural nucleobases, cannot be effectively used to manipulate the structures and functions of RNA. In fact, there are a number of diseases that are considered ‘undrugable’ by small molecules that can be corrected by targeting single-stranded RNA^61^—case in point are the recent development and approval of nusinersen (Spinraza) by Ionic Pharmaceuticals and patisiran (Onpattro) by Alnylam Pharmaceuticals for treatment of the respective spinal muscular atrophy and hereditary transthyretin-mediated amyloidosis. The present work provides another dimension in the design of ‘millamolecular’ oligonucleotide molecules for targeting nucleic acid biopolymers, especially those structured regions that may be difficult to access by conventional reagents.

In summary, we have shown that γPNA containing a selected set of JBs could be developed and could hybridize to both strands of the DNA double helix without undergoing self-hybridization. JBγPNA is able to invade a highly stable double helical B-DNA at a physiologically relevant ionic strength, where a homolog containing natural nucleobases is not. Overall, the bifacial recognition mode is general, applicable to targeting not only double helical DNA, but also RNA. Due to their tight binding, significantly shorter probes could potentially be used to target the secondary and tertiary structures of RNA in our efforts to decipher their physiological roles and to develop molecular therapies for treating genetic disorders.

## Methods

### Molecular dynamics simulations.

See Supplementary Methods.

### UV-melting analysis.

All UV-melting samples were prepared by mixing probes with DNA targets at the indicated concentrations and in the appropriate buffers, and annealed by incubation at 90 °C for 5 min followed by gradual cooling to room temperature. UV melting curves were collected using Agilent Cary UV-Vis 300 spectrometer equipped with a thermoelectrically controlled multi-cell holder. UV-melting spectra were collected by monitoring UV-absorption at 260 nm from 20 to 95 °C in the heating runs, and from 95 to 20 °C in the cooling runs, both at the rate of 1 °C per min. The heating and cooling curves were nearly identical, indicating that the hybridization process is reversible. The recorded spectra were smoothed using a 10-point adjacent averaging algorithm. The first-order derivatives of the melting curves were taken to determine the melting temperatures of the duplex and single strand.

### Circular dichroism analysis.

The samples were prepared in a 1× PBS buffer. All circular dichroism (CD) spectra represent an average of at least 15 scans collected at a rate of 100 nm/min between 200–400 nm, in a 1-cm path-length cuvette at 25 ° C. The CD spectrum from buffer solution was subtracted from the sample spectra, which were then smoothed via a 10-point adjacent averaging algorithm.

### Nuclear magnetic resonance analysis.

All 1D nuclear magnetic resonance (NMR) spectra for DNA and DNA:PNA complex were recorded on a 500 MHz spectrometer using Watergate p3919gp in 90:10% H_2_O:D_2_O and in D_2_O solvent. All 2D NOESY spectra for DNA and DNA:PNA complex were recorded on a 500 MHz spectrometer using Watergate NOESY or noesygpph19 as a pulse program with 100, 200, and 300 ms time scale. All COSY spectra were recorded using cosygpprqf as the pulse program.

### Mass spectrometric analysis.

For PNA, a solution of α-cyano-4-hydroxycinnamic acid (10 mg of α-cyano-4-hydroxycinnamic acid in 500 μL of water with 0.1% TFA and 500 μL of acetonitrile with 0.1% TFA) was used as the matrix for MALDI-TOF analysis. The PNA samples were prepared by mixing with 2 μL of matrix and 1 μL PNA (1–5μM) at 37 °C. MALDI-TOF analysis was performed about 10 min after spotting. For DNA and DNA:PNA complex, a solution of 6-aza-2-thiothymine (ATT, 10 mg/mL) in 1:1 (v:v) CH_3_CN/ammonium citrate [20 mM] were prepared. The DNA and DNA:PNA complex were prepared by mixing 2 μL of matrix and 1 μL of DNA or DNA:PNA complex (either in RNAse-free water or in a 1× PBS buffer). The MALDI-TOF plate was dried in vacuum desiccator for 10 min after spotting the sample and analyzed by MALDI-TOF MS.

### Electrophoretic mobility-shift assay.

All the DNA samples were prepared in the indicated buffers, either in 1× PBS (137 mM NaCl, 2.7 mM KCl, 10 mM NaPi, pH 7.4) or in a PR buffer (137 mM NaCl, 2.7 mM KCl, 10 mM NaPi, 2 mM MgCl_2_, pH 7.4), and annealed by heating to 90 °C for 5 min followed by gradual cooling to room temperature. PNA and DNA targets were mixed at the indicated concentrations and incubated at 37 °C for 4 h. The samples were then loaded onto 10% non-denaturing PAGE with 1× Tris-borate buffer and electrophoretically separated at 120 V for 60 min. The fluorescent probe attached to DNA was visualized by UV-Transilluminator. The gels without fluorescent probe were stained with SYBR-Gold and visualized by UV-Transilluminator.

## Supplementary Material

Supplemental Material

Movie1

Movie2

Data1

Data2

## Figures and Tables

**Table 1:** Thermodynamic parameters

Complex	T_m_ (°C)	−Δ*H*° (kJ/mol)	−TΔ*S*° (kJ/mol)	−Δ*G*° (kJ/mol)

W1-C1	37	244 ± 22	202 ± 24	42 ± 3
W1-P1	74	288 ±8	213 ± 6	75 ±1
P1-C1	40	ND	ND	ND
W1-P2	72	325 ± 17	250 ± 13	75 ± 3

*ND* not determined

## Data Availability

All data are available from the corresponding authors upon reasonable request.

## References

[1] Watson JD & Crick FHC A structure for deoxyribose nucleic acid. Nature 171, 737–738 (1953). 13054692 10.1038/171737a0

[2] Kopka ML, Yoon C, Goodsell D, Pjura P. & Dickerson RE The molecular origin of DNA-drug specificity in netropsin and distamycin. Proc. Natl Acad. Sci. USA 82, 1376–1380 (1985). 2983343 10.1073/pnas.82.5.1376 PMC397264

[3] Brown DD The role of stable complexes that repress and activate eucaryotic genes. Cell 37, 359–365 (1984). 6722879 10.1016/0092-8674(84)90366-0

[4] Kielkopf CL A structural basis for recognition of A·T and T·A base pairs in the minor groove of B-DNA. Science 282, 111–115 (1998). 9756473 10.1126/science.282.5386.111

[5] Sun JS & Helene C. Oligonucleotide-directed triple-helix formation. Curr. Opin. Struct. Biol 3, 345–356 (1993). 10.1016/s0959-440x(96)80051-0 8804836

[6] Greisman HA & Pabo CO A general strategy for selecting high-affinity zinc finger proteins for diverse DNA target sites. Science 275, 657–661 (1997). 9005850 10.1126/science.275.5300.657

[7] Jantz D, Amann BT, Gatto GJ & Berg JM The design of functional DNA-binding proteins based on zinc finger domains. Chem. Rev 104, 789–800 (2004). 14871141 10.1021/cr020603o

[8] Moscou MJ & Bogdanove AJ A simple cipher governs DNA recognition by TAL effectors. Science 326, 1501 (2009). 19933106 10.1126/science.1178817

[9] Mak AN-S, Bradley P, Cernadas RA, Bogdanove AJ & Stoddard BL The crystal structure of TAL effector PthXo1 bound to its DNA target. Science 335, 716–719 (2012). 22223736 10.1126/science.1216211 PMC3427646

[10] Jinek M. A programmable dual-RNA-guided DNA endonuclease in adaptive bacterial immunity. Science 337, 816–821 (2012). 22745249 10.1126/science.1225829 PMC6286148

[11] Lander ES The heroes of CRISPR. Cell 164, 18–28 (2016). 26771483 10.1016/j.cell.2015.12.041

[12] Tsai SQ GUIDE-seq enables genome-wide profiling of off-target cleavage by CRISPR-Cas nucleases. Nat. Biotech 33, 187–197 (2015). 10.1038/nbt.3117 PMC4320685 25513782

[13] Wang X. Unbiased detection of off-target cleavage by CRISPR-Cas9 and TALENs using integrase-defective lentiviral vectors. Nat. Biotech 33, 175–178 (2015). 10.1038/nbt.3127 25599175

[14] Nielsen PE, Egholm M, Berg RH & Buchardt O. Sequence-selective recognition of DNA by strand displacement with a thymine-substituted polyamide. Science 254, 1497–1500 (1991). 1962210 10.1126/science.1962210

[15] Nielsen PE Peptide nucleic acid. A molecule with two identities. Acc. Chem. Res 32, 624–630 (1999).

[16] Nielsen PE & Christensen L. Strand displacement binding of a duplexforming homopurine PNA to a homopyrimidine duplex DNA target. J. Am. Chem. Soc 118, 2287–2288 (1996).

[17] Egholm M. PNA hybridizes to complementary oligonucleotides obeying the Watson-Crick hydrogen-bonding rules. Nature 365, 566–568 (1993). 7692304 10.1038/365566a0

[18] Kaihatsu K, Shah RH, Zhao X. & Corey DR Extending recognition by peptide nucleic acids (PNAs): binding to duplex DNA and inhibition of transcription by tail-clamp PNA-peptide conjugate. Biochemistry 42, 13996–14003 (2003). 14636068 10.1021/bi035194k

[19] Bentin T, Larsen HJ & Nielsen PE Combined triplex/duplex invasion of double-stranded DNA by “tail-clamp” peptide nucleic acid. Biochemistry 42, 13987–13995 (2003). 14636067 10.1021/bi0351918

[20] Lohse J, Dahl O. & Nielsen PE Double duplex invasion by peptide nucleic acid: A general principle for sequence-specific targeting of double-stranded DNA. Proc. Natl Acad. Sci. USA 96, 11804–11808 (1999). 10518531 10.1073/pnas.96.21.11804 PMC18367

[21] Demidov VV Kinetics and mechanism of the DNA double helix invasion by pseudocomplementary peptide nucleic acids. Proc. Natl Acad. Sci. USA 99, 5953–5958 (2002). 11972051 10.1073/pnas.092127999 PMC122883

[22] Bahal R, Sahu B, Rapireddy S, Lee C-M & Ly DH Sequence-unrestricted, Watson-Crick recognition of double helical B-DNA by (R)MiniPEG-gPNAs. Chembiochem 13, 56–60 (2012). 22135012 10.1002/cbic.201100646 PMC10206777

[23] Singer A, Rapireddy S, Ly DH & Meller A. Electronic barcoding of a viral gene at the single-molecule level. Nano. Lett 12, 1722–1728 (2012). 22352964 10.1021/nl300372a PMC3572535

[24] Nolling J. Duplex DNA-invading y-modified peptide nucleic acids enable rapid identification of bloodstream infections in whole blood. mBio 7, e00345–00316 (2016). 10.1128/mBio.00345-16 PMC4850259 27094328

[25] Morin TJ Nanopore-based target sequence detection. PLoS One 11, e0154426 (2016). 10.1371/journal.pone.0154426 PMC4858282 27149679

[26] Bahal R. In vivo correction of anemia in b-thalassemic mice by yPNA-mediated gene editing with nanoparticle delivery. Nat. Commun 7, 13304 (2016). 27782131 10.1038/ncomms13304 PMC5095181

[27] Rapireddy S, Bahal R. & Ly DH Strand invasion of mixed-sequence, double-helical B-DNA by g-peptide nucleic acis containing G-clamp nucleobases under physiological conditions. Biochemistry 50, 3913–3918 (2011). 21476606 10.1021/bi2002554 PMC3092786

[28] Haaima G, Hansen HF, Christensen L, Dahl O. & Nielsen PE Increased DNA binding and sequence discrimination of PNA oligomers containing 2,6-diaminopurine. Nucleic Acids Res. 25, 4639–4643 (1997). 9358176 10.1093/nar/25.22.4639 PMC147079

[29] Chenna V. A simple cytosine-to-G-clamp nucleobase substitution enables chiral gamma-PNAs to invade mixed-sequence double helical B-form DNA. Chembiochem 9, 2388–2391 (2008). 18816545 10.1002/cbic.200800441 PMC9716473

[30] Branda N. & Lehn J-M JANUS WEDGES: A new approach towards nucleobase-pair recognition. Chem. Commun 0, 2443–2444 (1996).

[31] Chen D, Meena, Sharma, S. K. & McLaughlin, L. W. Formation and stability of a janus-wedge type of DNA triplex. J. Am. Chem. Soc 126, 70–71 (2004). 14709064 10.1021/ja038081x

[32] Chen H, McLaughlin M. & McLaughlin LM A janus-wedge DNA triplex with A-W1-T and G-W2-C base triplets. J. Am. Chem. Soc 130, 13190–13191 (2008). 18783217 10.1021/ja804607v

[33] Arambula JF, Ramisetty SR, Baranger AM & Zimmerman SC A simple ligand that selectively targets CUG trinucleotide repeats and inhibits MBNL protein binding. Proc. Natl Acad. Sci. USA 106, 16068–16073 (2009). 19805260 10.1073/pnas.0901824106 PMC2752522

[34] Zhao H. Synthesis of a complete Janus-type guanosine-cytosine base and its 2’-deoxyribonucleoside. Chem. Lett 40, 684–686 (2011).

[35] Shin D. & Tor Y. Bifacial nucleoside as a surrogate for both T and A in duplex DNA. J. Am. Chem. Soc 133, 6926–6929 (2011). 21495708 10.1021/ja201397e PMC3093761

[36] Zeng Y, Pratumyot Y, Piao X. & Bong D. Discrete assembly of synthetic peptide-DNA triplex structures from polyvalent melamine-thymine bifacial recognition. J. Am. Chem. Soc 134, 832–835 (2012). 22201288 10.1021/ja2099326

[37] Artigas G. & Marchan V. Synthesis of janus compounds for the recognition of G-U mismatched nucleobase pairs. J. Org. Chem 78, 10666–10677 (2013). 24087986 10.1021/jo401684j

[38] Robinson CJ Modular riboswitch toolsets for synthetic genetic control in diverse bacterial species. J. Am. Chem. Soc 136, 10615–10624 (2014). 24971878 10.1021/ja502873j

[39] Yeh JI Crystal structure of chiral gammaPNA with complementary DNA strand: Insights into the stability and specificity of recognition and conformational preorganization. J. Am. Chem. Soc 132, 10717–10727 (2010). 20681704 10.1021/ja907225d PMC2929025

[40] Macke TJ & Case DA Modeling unusual nucleic acid structures. Molecular Modeling of Nucleic Acids. 379–393 (1997).

[41] He W. The structure of a gamma-modified peptide nucleic acid duplex. Mol. Biosyst 6, 1619–1629 (2010). 20386807 10.1039/c002254c

[42] Uhlmann E, Peyman A, Breipohl G. & Will DW PNA: synthetic polyamide nucleic acids with unusual binding properties. Angew. Chem., Int. Ed 37, 2796–2823 (1998). 10.1002/(SICI)1521-3773(19981102)37:20<2796::AID-ANIE2796>3.0.CO;2-K 29711102

[43] Dragulescu-Andrasi A. A simple gamma-backbone modification preorganizes peptide nucleic acid into a helical structure. J. Am. Chem. Soc 128, 10258–10267 (2006). 16881656 10.1021/ja0625576

[44] Thadke SA Design of bivalent nucleic acid ligands for recognition of RNA-repeated expansion associated with Huntington’s disease. Biochemistry 57, 2094–2108 (2018). 29562132 10.1021/acs.biochem.8b00062 PMC6091552

[45] Santoro SW & Joyce GF A general purpose RNA-cleaving DNA enzyme. Proc. Natl Acad. Sci. USA 94, 4262–4266 (1997). 9113977 10.1073/pnas.94.9.4262 PMC20710

[46] Mortimer SA, Kidwell MA & Doudna JA Insights into RNA structure and function from genome-wide studies. Nat. Rev. Gen 15, 469–479 (2014). 10.1038/nrg3681 24821474

[47] Smith LM, Andersen KB, Hovgaard L. & Jaroszewski JW Rational selection of antisense ogligonucleotide sequences. Eur. J. Pharm. Sci 11, 191–198 (2000). 10.1016/s0928-0987(00)00100-7 11042224

[48] Tu G.-c, Cao Q.-n, Zhou F. & Israel Y. Tetranucleotide GGGA motif in primary RNA transcripts. J. Biol. Chem 273, 25125–25131 (1998). 9737971 10.1074/jbc.273.39.25125

[49] Thomas JR & Hergenrother PJ Targeting RNA with small molecules. Chem. Rev 108, 1171–1224 (2008). 18361529 10.1021/cr0681546

[50] Guan L. & Disney MD Recent advances in developing small molecules targeting RNA. Acs. Chem. Biol 7, 73–86 (2012). 22185671 10.1021/cb200447r

[51] Corey DR Nusinersen, an antisense oligonucleotide drug for spinal muscular atrophy. Nat. Neurosci 20, 497–499 (2017). 28192393 10.1038/nn.4508

[52] Garber K. Alnylam launches era of RNAi drugs. Nat. Biotech 36, 777–778 (2018). 10.1038/nbt0918-777 30188543

[53] Wheeler TM Targeting nuclear RNA for in vivo correction of myotonic dystrophy. Nature 488, 111–115 (2012). 22859208 10.1038/nature11362 PMC4221572

[54] Brauns EB, Murphy CJ & Berg MA Local dynamics in DNA by temperature-dependent Stokes Shift of an intercalated dye. J. Am. Chem. Soc 120, 2449–2456 (1998).

[55] He G, Rapireddy S, Bahal R, Sahu B. & Ly DH Strand invasion of extended, mixed sequence B-DNA by gammaPNAs. J. Am. Chem. Soc 131, 12088–12090 (2009). 19663424 10.1021/ja900228j PMC2742410

[56] Singh V, Fedeles BI & Essignmann JM Role of tautomerism in RNA biochemistry. RNA 21, 1–13 (2015). 25516996 10.1261/rna.048371.114 PMC4274630

[57] Zhang DY & Seelig G. Dynamic DNA nanotechnology using strand-displacement reactions. Nat. Chem 3, 103–113 (2011). 21258382 10.1038/nchem.957

[58] Zhang DY & Winfree E. Control of DNA strand displacement kinetics using toeold exchange. J. Am. Chem. Soc 131, 17303–17314 (2009). 19894722 10.1021/ja906987s

[59] Stanzl EG, Trantow BM, Vargas JR & Wender PA Fifteen years of cell-penetrating, guanidinium-rich molecular transporters: Basic science, research tools, and clinical applications. Acc. Chem. Res 46, 2944–2954 (2013). 23697862 10.1021/ar4000554 PMC3796152

[60] Corey DR 48,000-fold acceleration of hybridization of chemically modified oligonucleotides. J. Am. Chem. Soc 117, 9373–9374 (1995).

[61] Rinaldi C. & Wood MJA Antisense oligonucleotides: the next frontier for treatment of neurological disorders. Nat. Rev. Neurol 14, 9–21 (2018). 29192260 10.1038/nrneurol.2017.148

