# Publisher Correction: Shape selective bifacial recognition of double helical DNA

**DOI:** 10.1038/s42004-020-0274-5

**Published:** 2020-02-19

**Authors:** Shivaji A. Thadke, V. M. Hridya, J. Dinithi R. Perera, Roberto R. Gil, Arnab Mukherjee, Danith H. Ly

**Affiliations:** 1 Department of Chemistry and Institute for Biomolecular Design and Discovery (IBD), Carnegie Mellon University, 4400 Fifth Avenue, Pittsburgh, PA 15213 USA; 2 Department of Chemistry, Indian Institute of Science Education and Research (IISER), Pune, Maharashtra 411008 India

Correction to: *Communications Chemistry* 10.1038/s42004-018-0080-5, published online 7 November 2018.

The original version of this Article incorrectly omitted the received/accepted dates of Received: 31 August 2018; Accepted: 15 October 2018. This has now been corrected in the PDF and HTML versions of the Article.

