# Supplementary material for: Shape selective bifacial recognition of double helical DNA
Source: Commun Chem. Author manuscript; Available in PMC 2023 Feb 13. (PMC9923363; doi:10.1038/s42004-018-0080-5)
Supplement: Supplemental Material [file NIHMS1849764-supplement-Supplemental_Material.pdf]

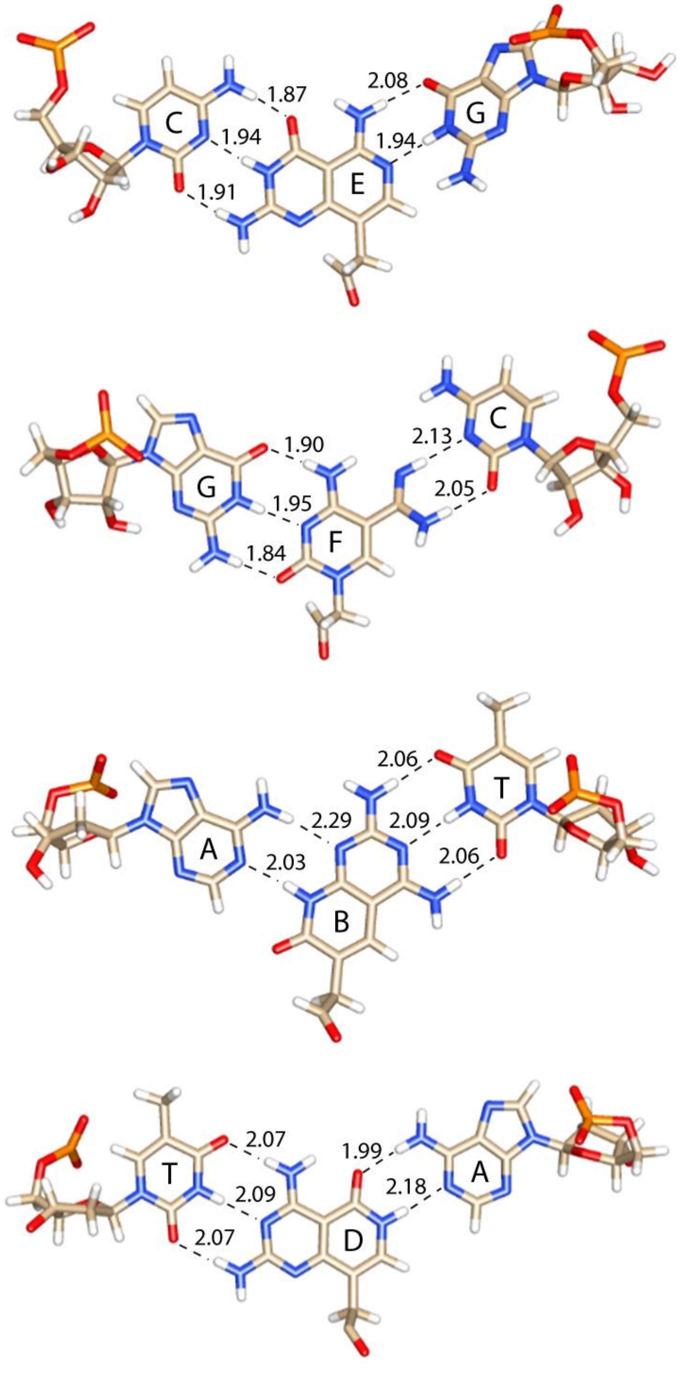

Supplementary Figure 1: Hydrogen-bonding interactions of JB-triads (C-E-G, G-F-C, A-B-T, and T-D-A). Note: A, G, C, and T are natural nucleobases, and E, F, B, and D are Janus bases. The backbone was omitted for clarity, and the hydrogen-bonding distances are in Å.

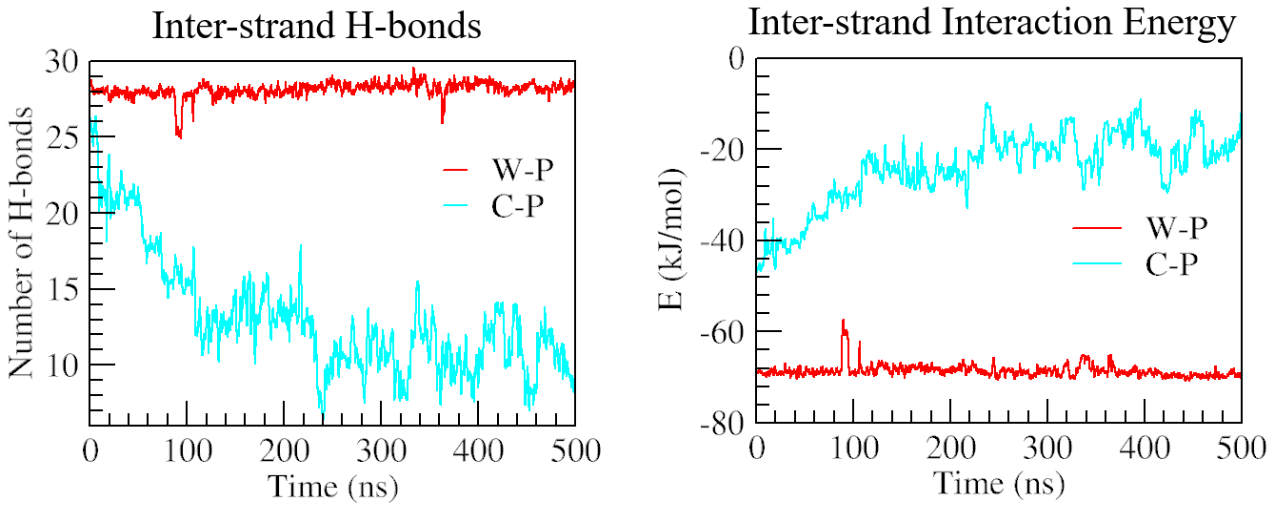

Supplementary Figure 2: H-bonding and inter-strand energy of W-P and C-P per base-pair (excluding terminal base-pair).

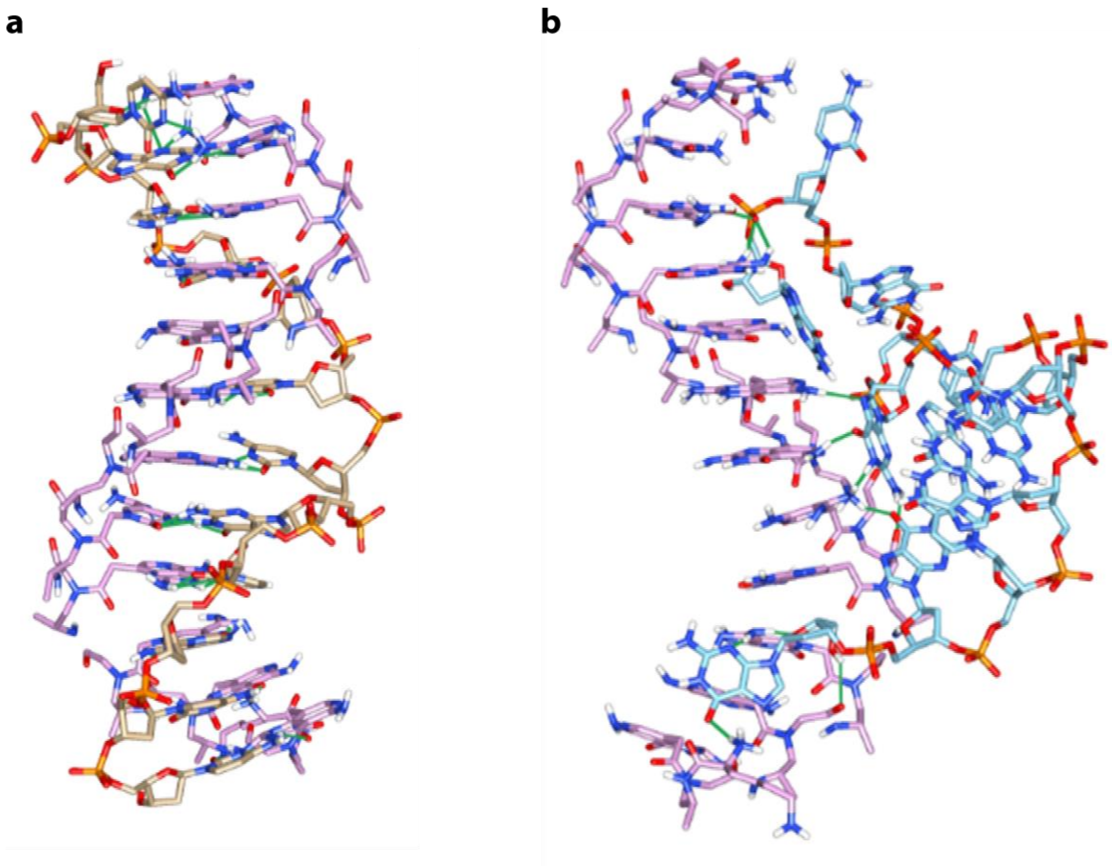

Supplementary Figure 3: MD simulated structure of (**a**) W1-P1 and (**b**) P1-C1 after 500 ns.

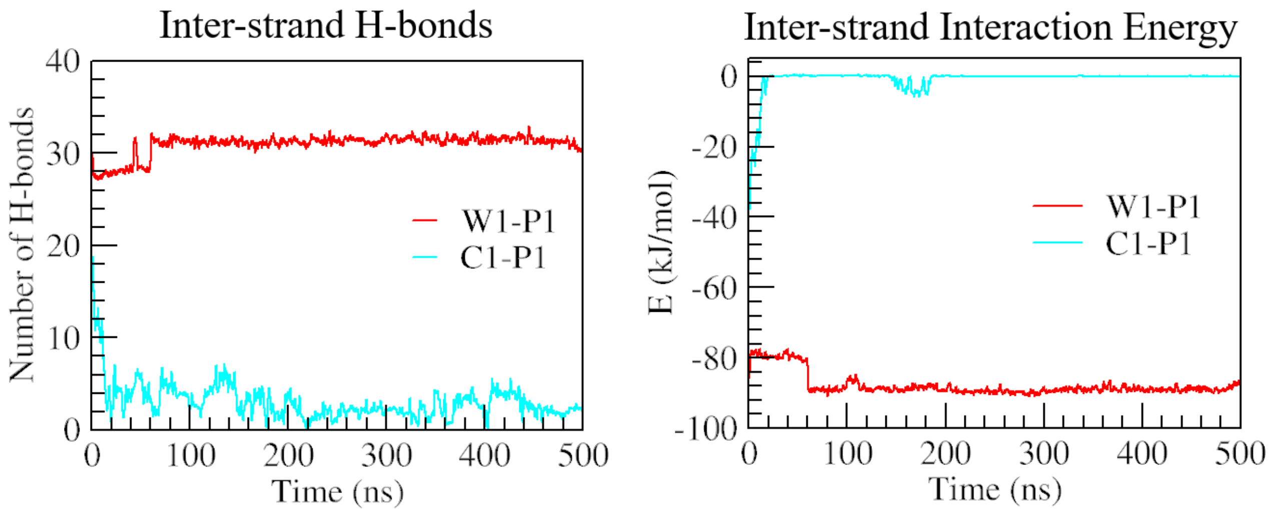

Supplementary Figure 4: H-bonding and inter-strand energy of W1-P1 and P1-C1 per base-pair (excluding terminal base pair).

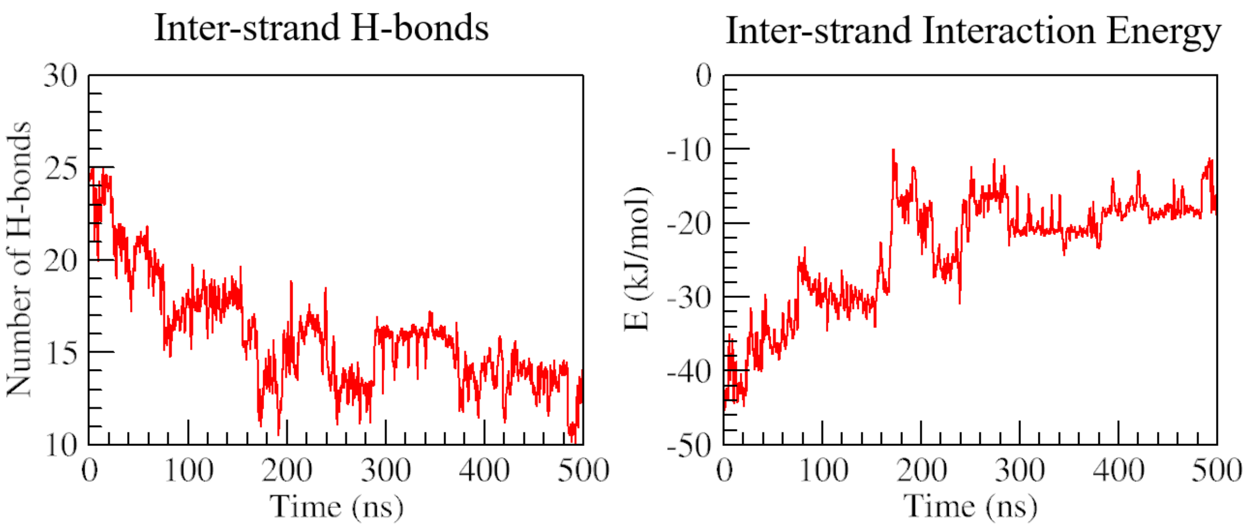

Supplementary Figure 5: H-bonding and inter-strand energy of P1-P1 per base-pair (excluding terminal base pair).

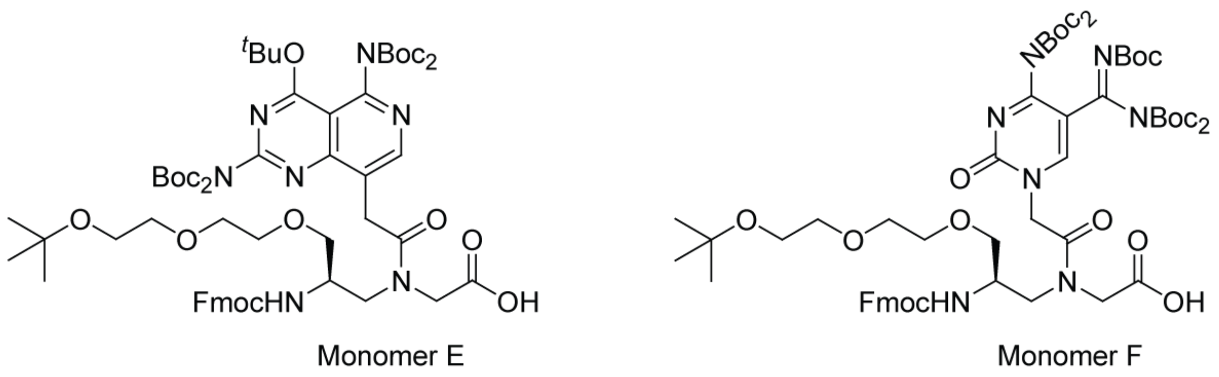

Supplementary Figure 6: Chemical structures of E- and F-γPNA monomers.

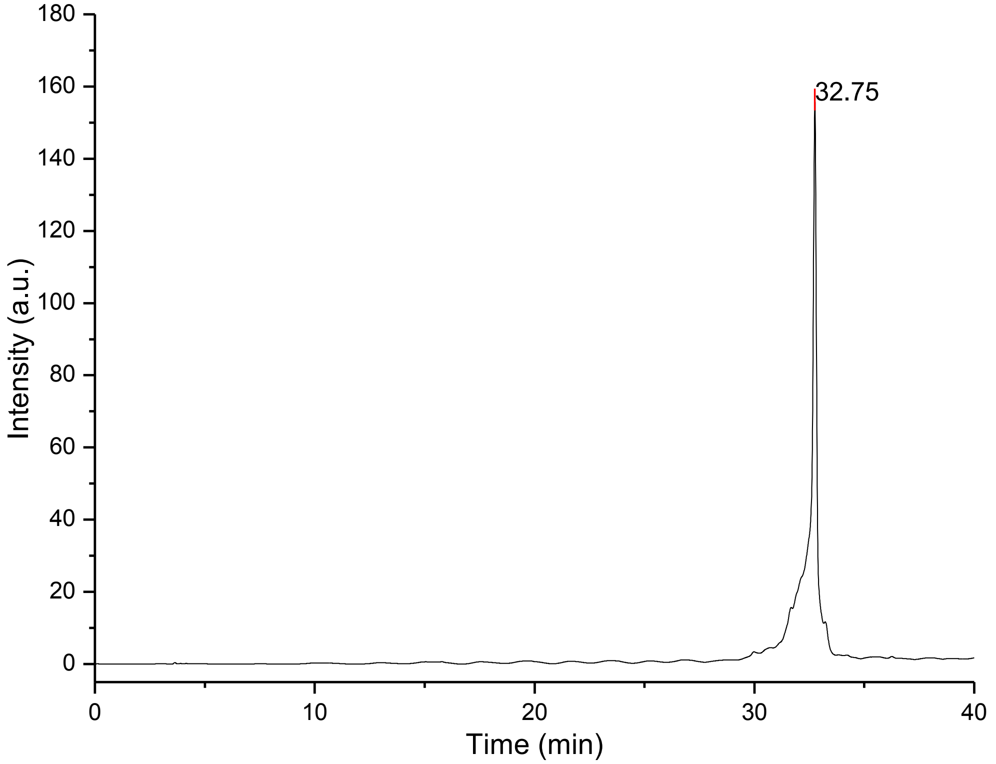

Supplementary Figure 7a: HPLC spectrum of P1.

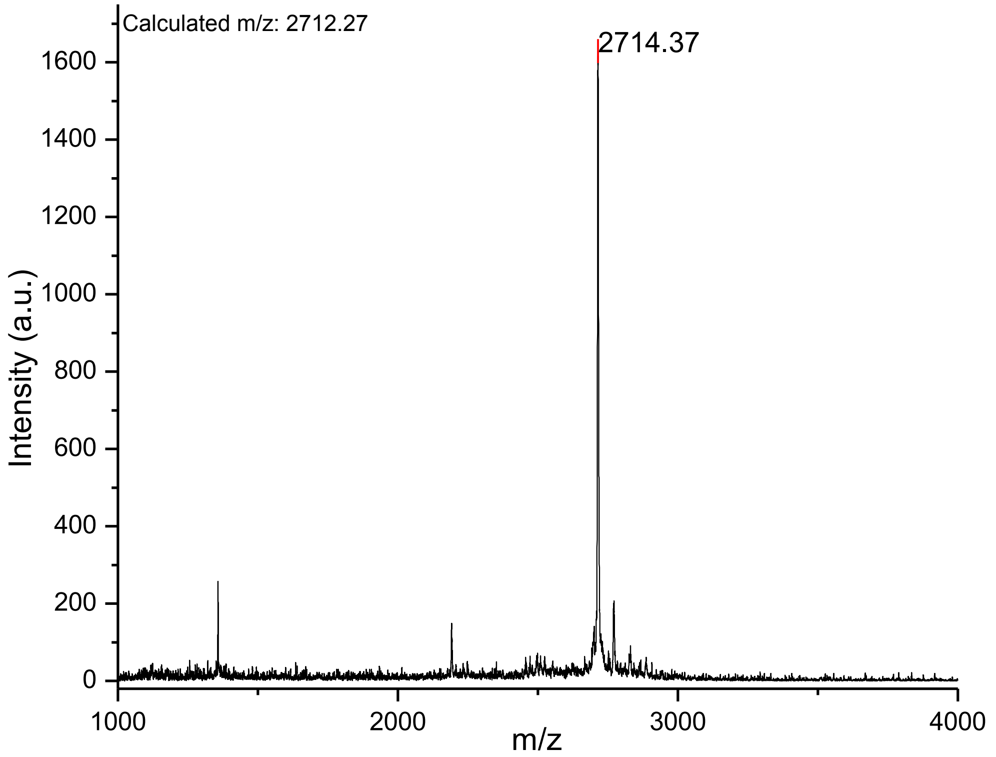

Supplementary Figure 7b: MALDI-TOF spectrum of P1.

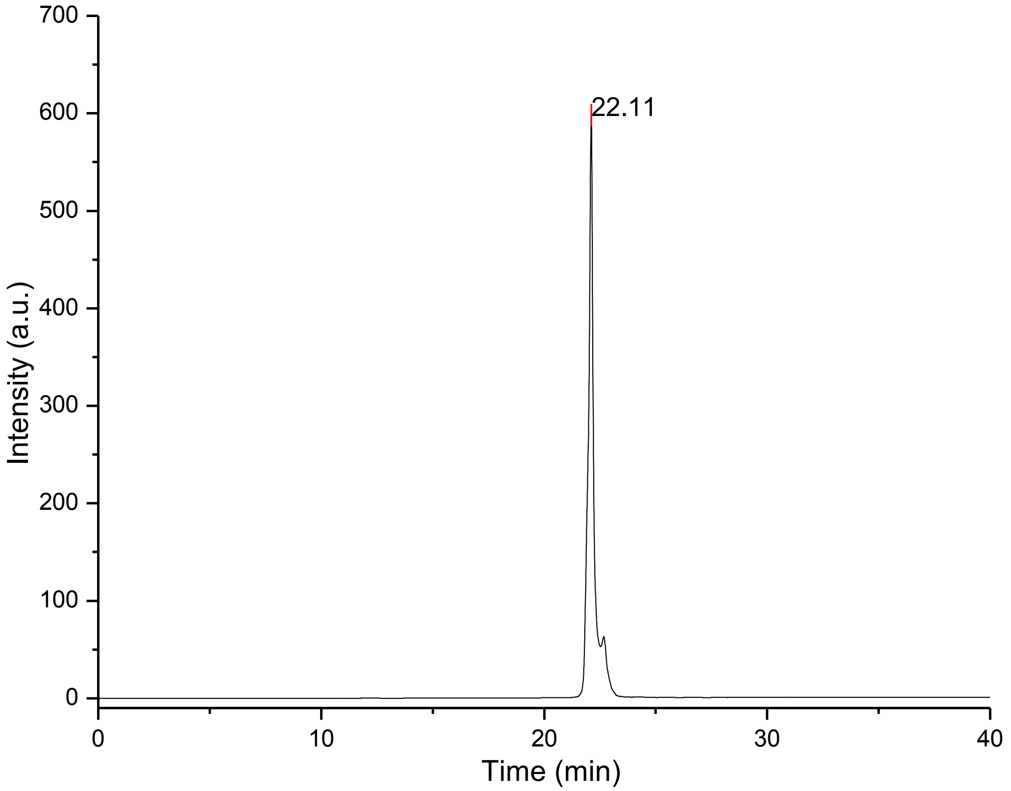

Supplementary Figure 8a: HPLC spectrum of P2.

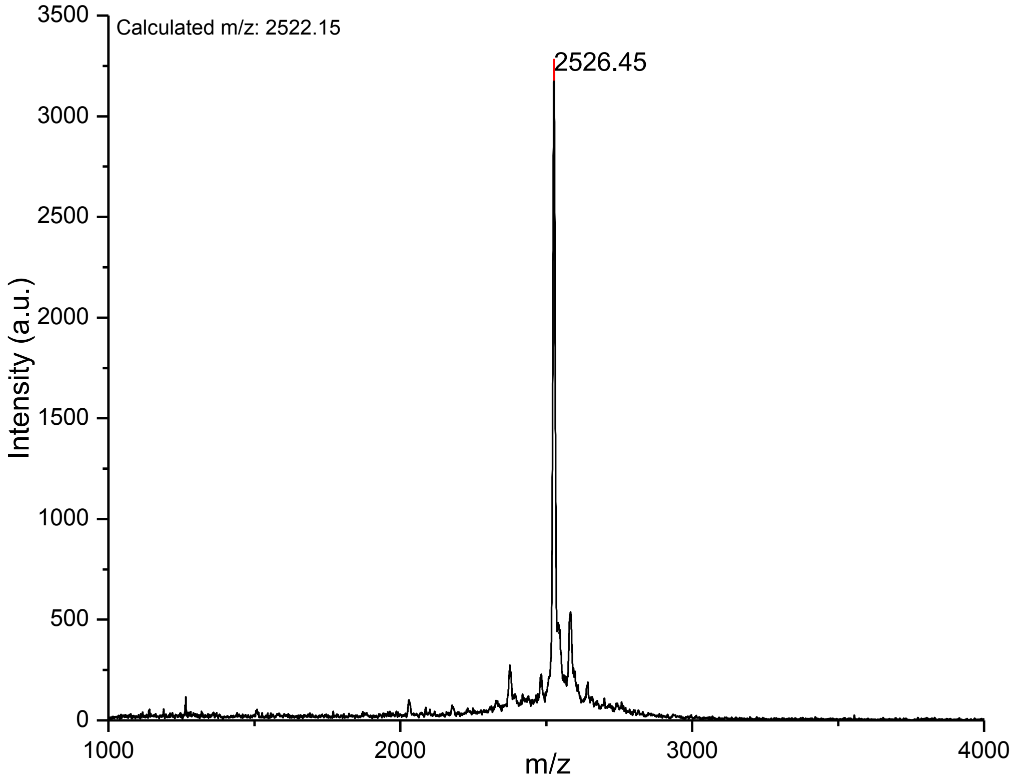

Supplementary Figure 8b: MALDI-TOF spectrum of P2.

a

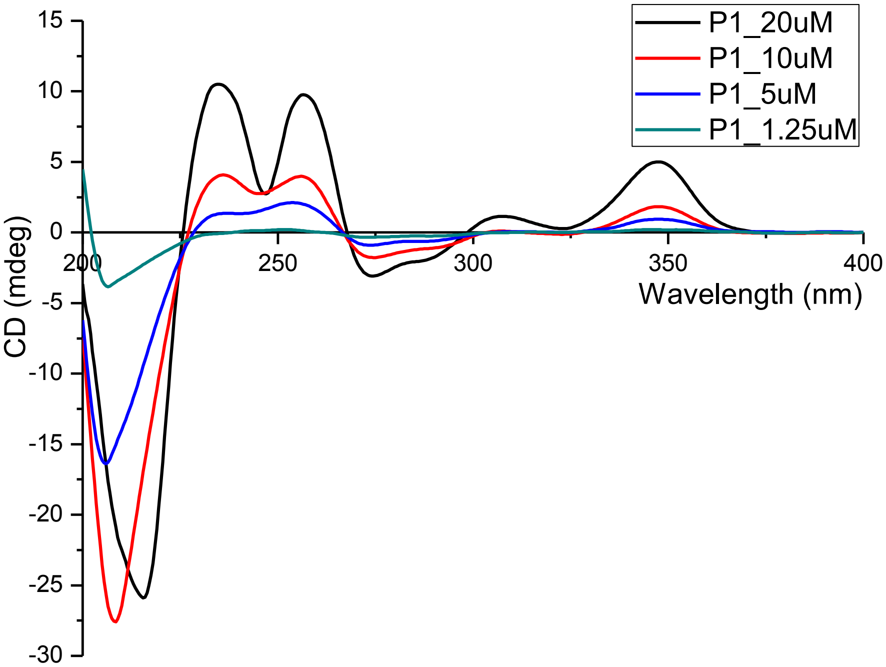

b

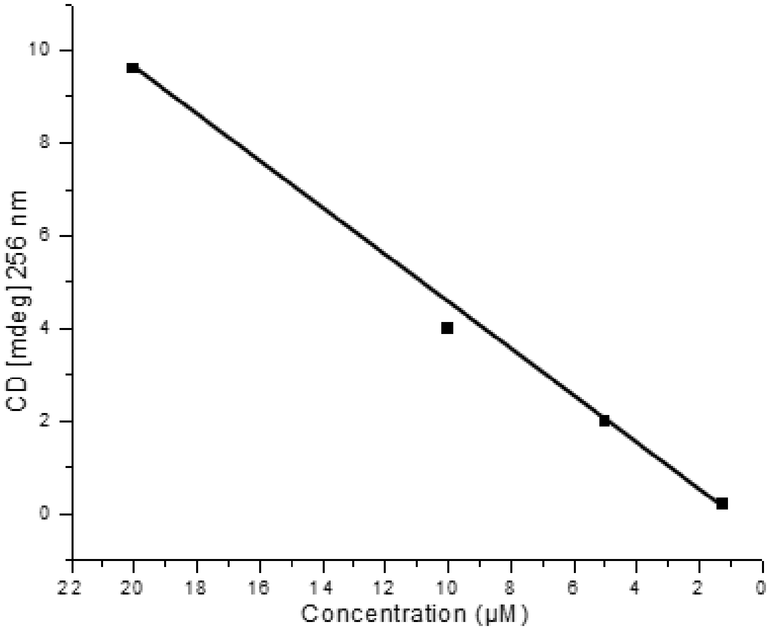

Supplementary Figure 9: Effects of concentration on CD signals. (**a**) Concentration-dependent CD spectra of P1 prepared in 1xPBS buffer. (**b**) The CD signal at 256 nm as a function of P1 concentration.

a

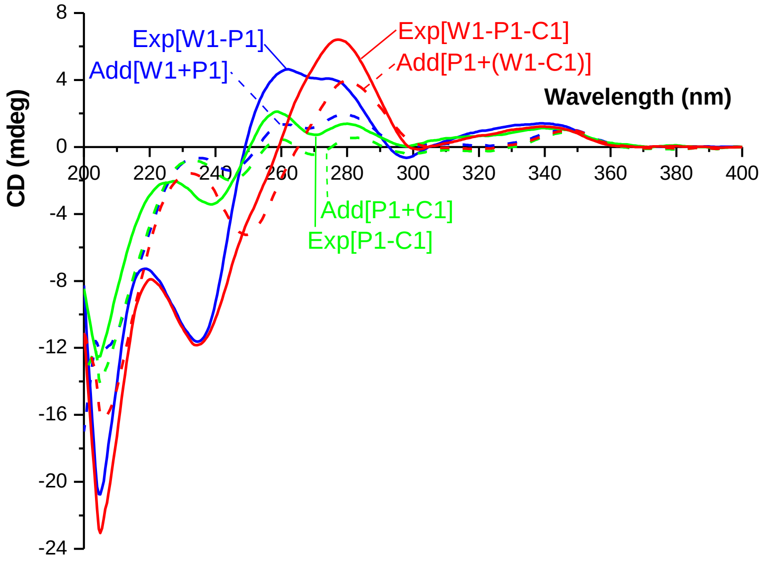

b

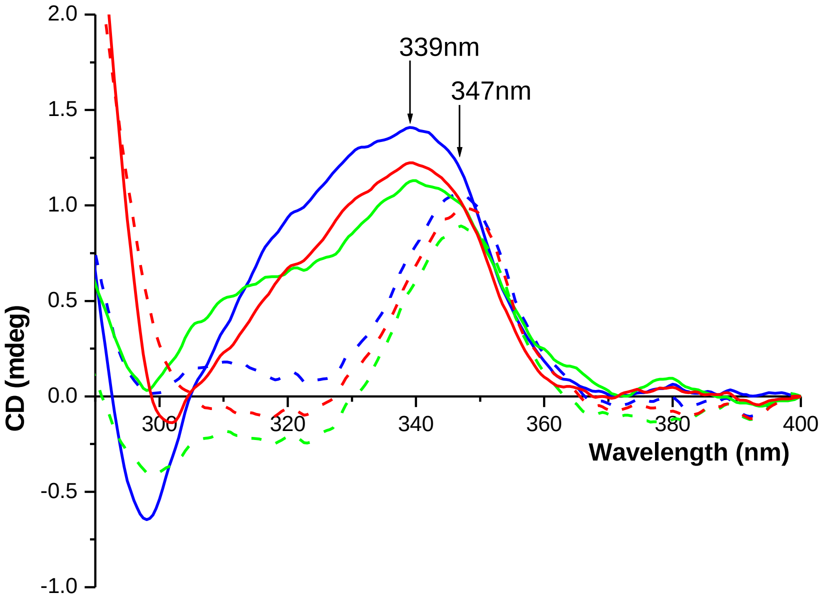

Supplementary Figure 10: CD spectra of the perfectly-matched complexes, along with additives of the individual strands. (**a**) The complexes: W1-P1 (blue line), P1-C1 (green line), and W1-P1-C1 (red line). Solid line: actual samples, dashed line: additives. (**b**) The same CD spectrum as in (**a**) but focused on the JB absorption regions (290-400 nm). The concentration of each strand was 2.5 µM, prepared in 1xPBS buffer. The samples were prepared by mixing pre-annealed DNA with P1 and incubated at 37 °C for 2 h prior to recording the CD spectra.

a

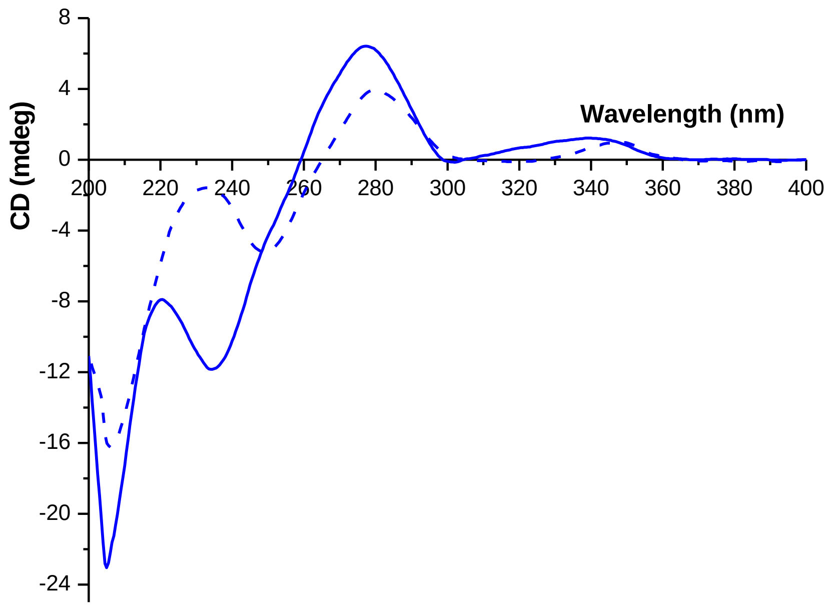

b

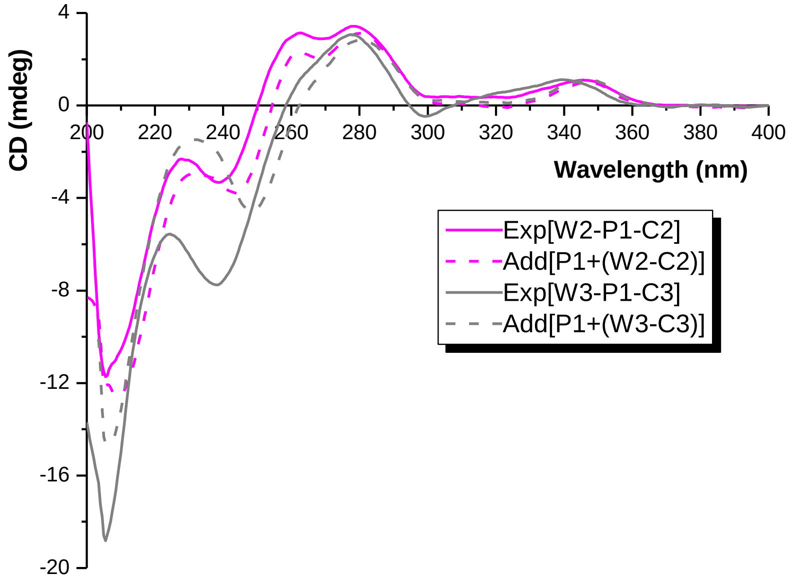

Supplementary Figure 11: CD spectra of the mismatched sequence and binding orientation. (**a**) CD spectra of W2-C2 (solid line) and W3-C3 (dashed line) duplexes. (b) CD spectra of the mismatched sequence W2-P1-C2 (magenta line) and binding orientation W3-P1-C3 (grey line), along with their additives (dashed lines). The samples were prepared in the same way as that described in **Supplementary Figure 10** caption.

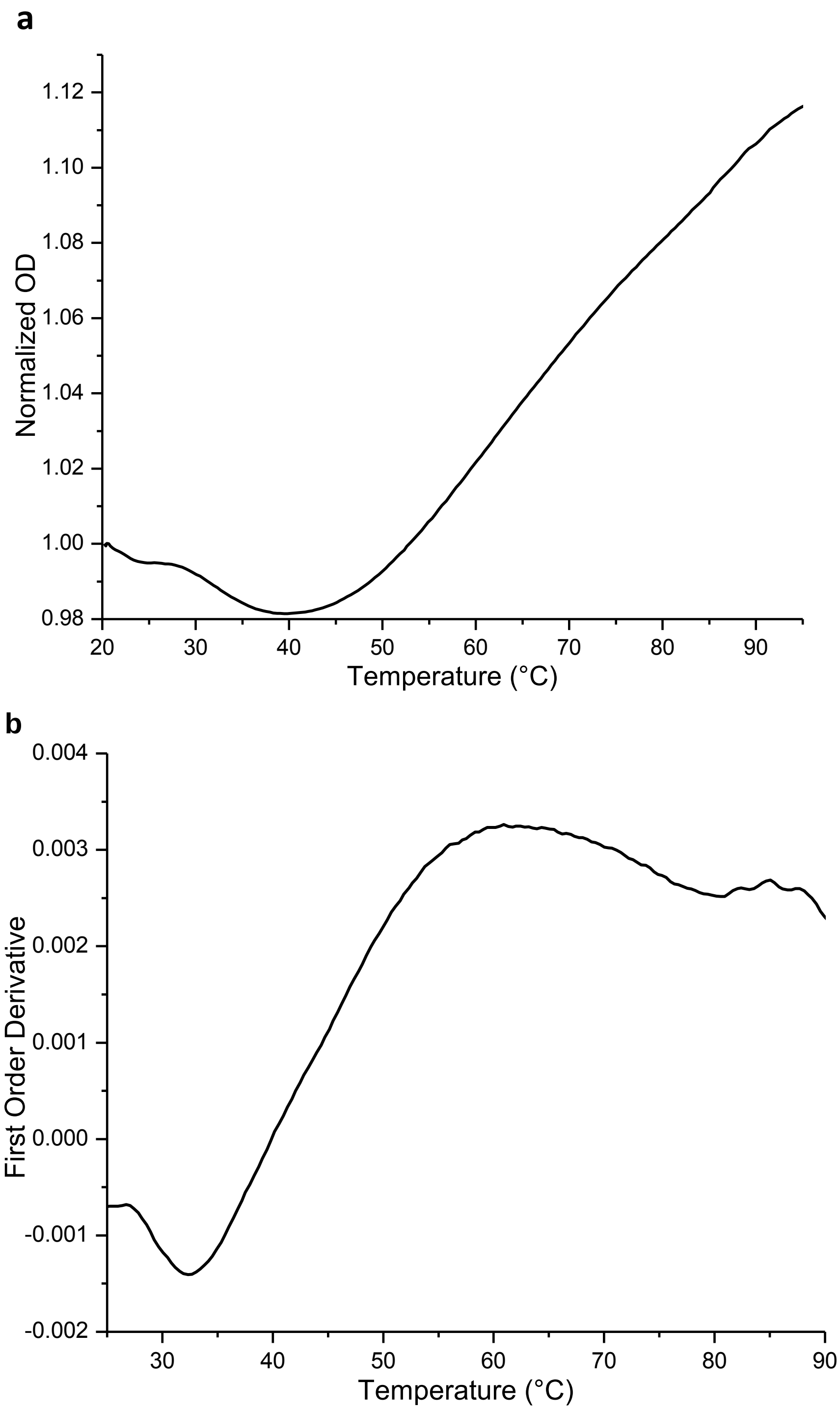

Supplementary Figure 12: Thermal stability of P1. (**a**) UV-melting profile of P1. (**b**) First-order derivative of P1 melting profile, T_m ~ 35-80 °C. The concentration of P1 was 2.5 µM, prepared in 1xPBS buffer.

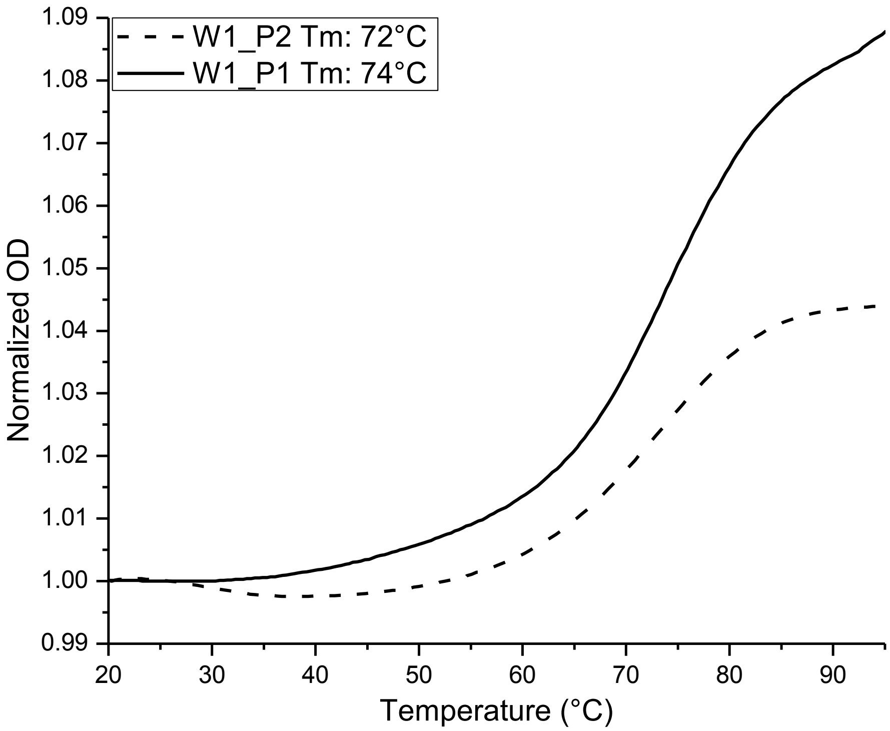

Supplementary Figure 13: UV-melting profiles of W1-P1 (solid line) and W1-P2 (dashed line). The concentration of each strand was 2.5 µM. The samples were prepared by mixing the indicated strands in 1xPBS buffer and annealed at 95 °C for 5 min, followed by a gradual cooling to room temperature before recording the UV-melting data.

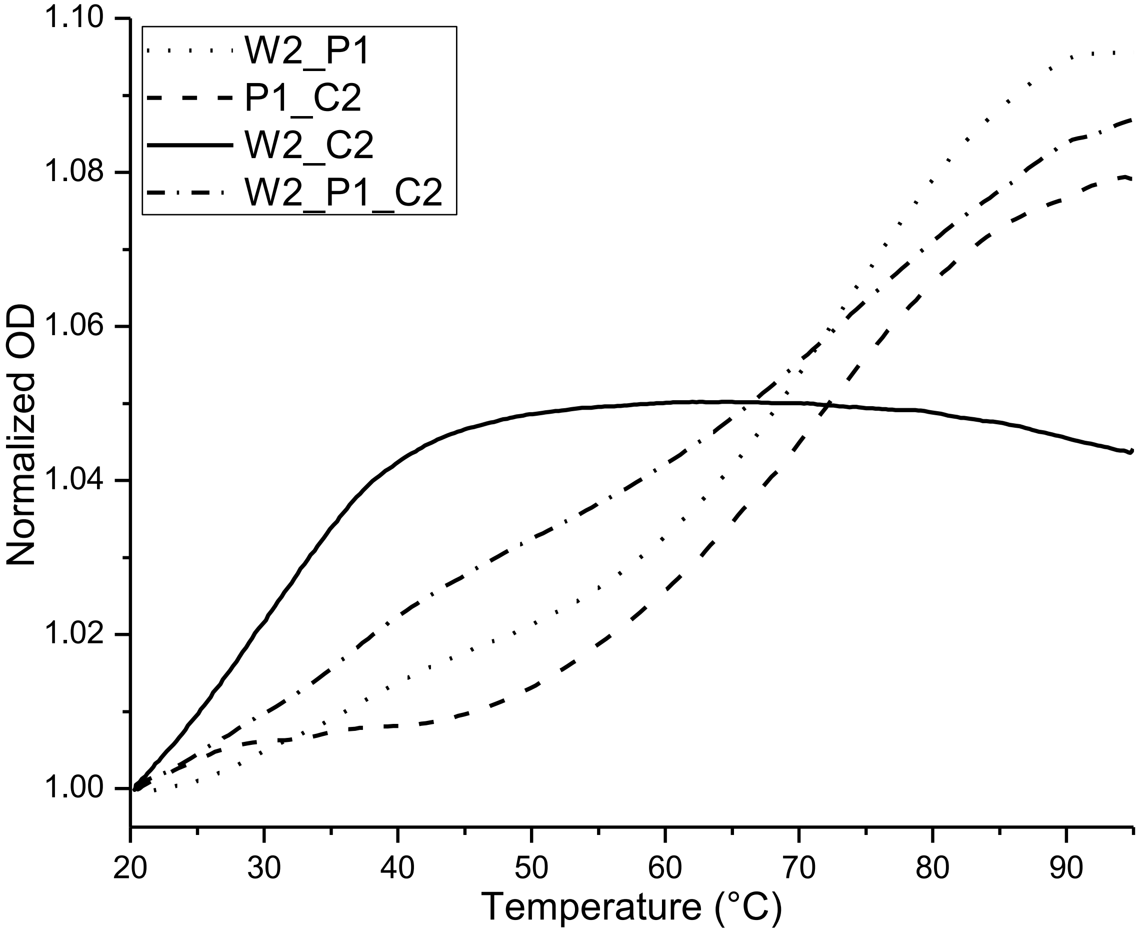

Supplementary Figure 14: UV-melting profiles of P1 with the mismatched sequence W2-C2 (solid line), W2-P1 (dotted line), P1-C2 (dashed line), and W2-P1-C2 (dashed dotted line). The concentrations and the experimental conditions were the same was those detailed in the **Fig.S13** caption.

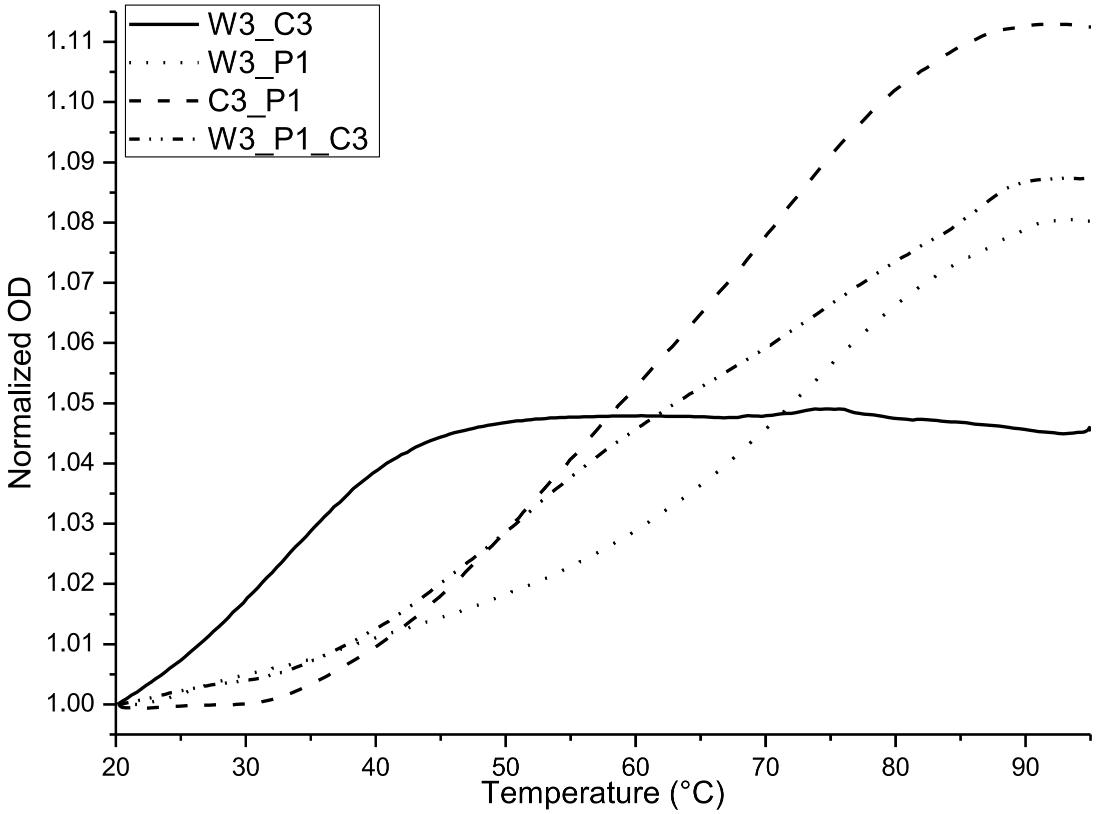

Supplementary Figure 15: UV-melting profiles of P1 with the mismatched binding-orientation W3-C3 (solid line), W3-P1 (dotted line), C3-P1 (dashed line), and W3-P1-C3 (dashed dotted line). The concentrations and the experimental conditions were the same was those detailed in the **Supplementary Figure 13** caption.

1D and 2D NMR Spectra[1-6]

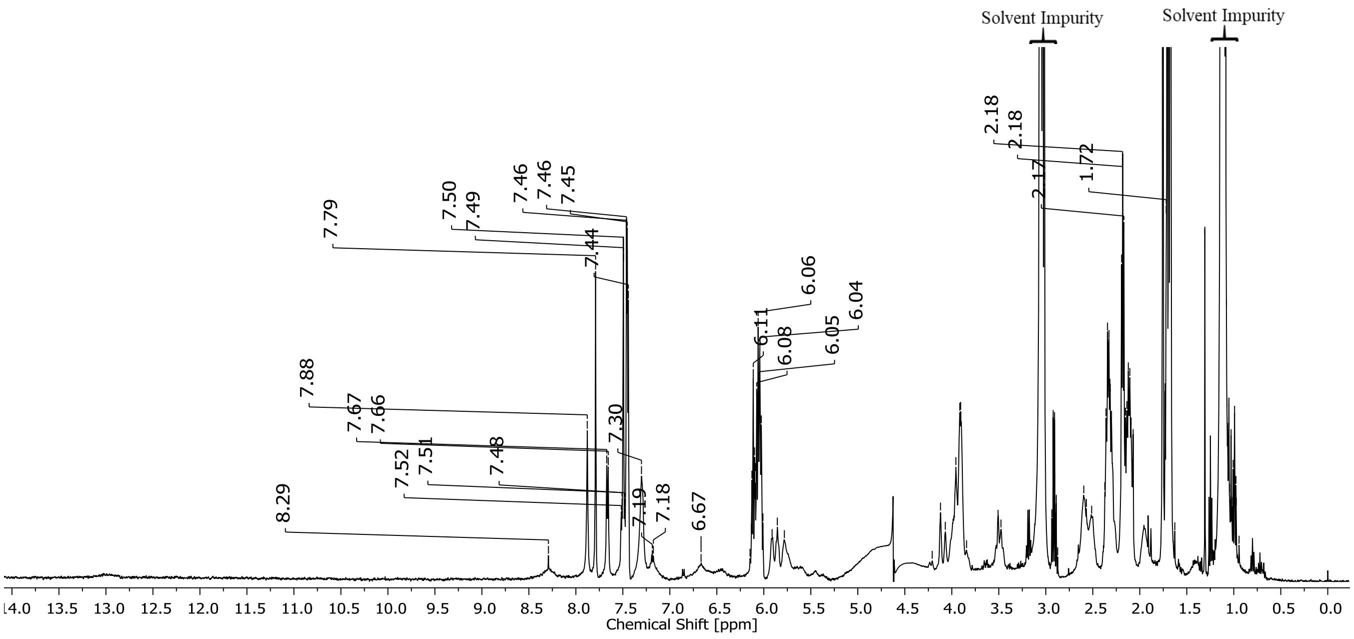

Supplementary Figure 16: ^{1}H-NMR (Watergate p3919gp Exp., 500.13 MHz, 90%H_2O:10%D_2O, 1K scans) spectrum of W1 at 25 °C. The concentration of W1 was 0.5 mM, prepared in 1xPBS buffer containing H_2O/D_2O at a 9:1 volume-ratio. The sample was annealed at 95 °C and gradually cooled to room temperature before acquisition of the ^{1}H-NMR data.

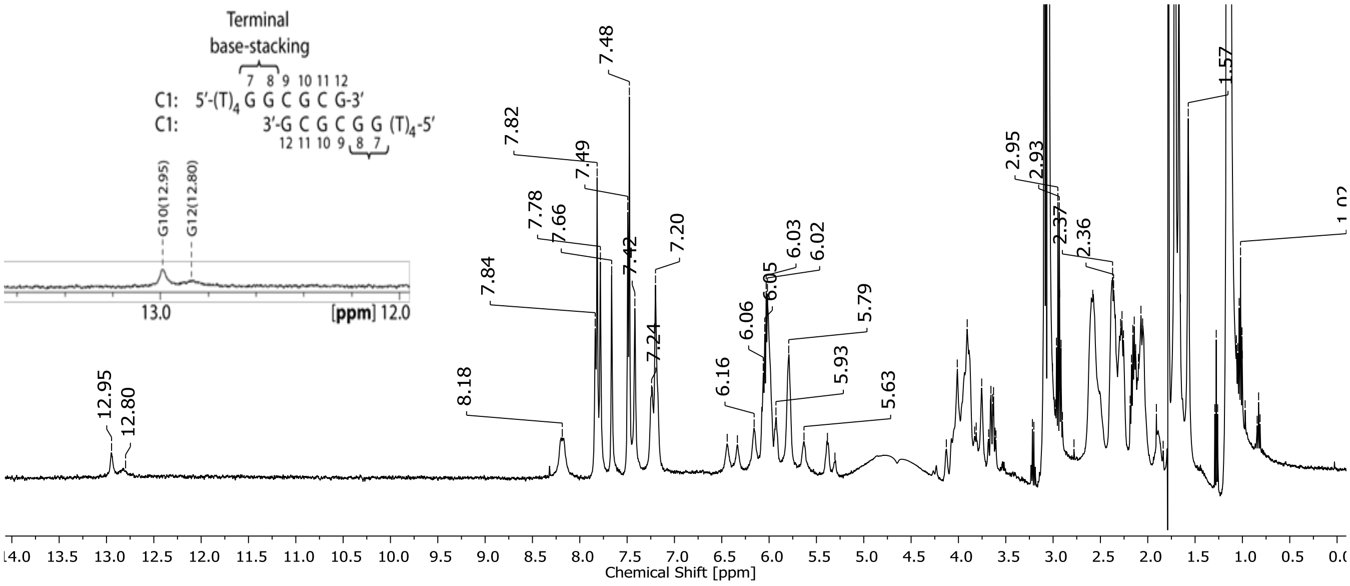

Supplementary Figure 17: ^{1}H-NMR (Watergate p3919gp Exp., 500.13 MHz, 90%H_2O:10%D_2O, 1K scans) spectrum of C1 at 25 °C. The concentration of C1 was 0.5 mM, prepared in 1X PBS buffer containing H_2O/D_2O at a 9:1 volume-ratio. The sample was annealed at 95 °C and gradually cooled to room temperature before acquisition of the ^{1}H-NMR data.

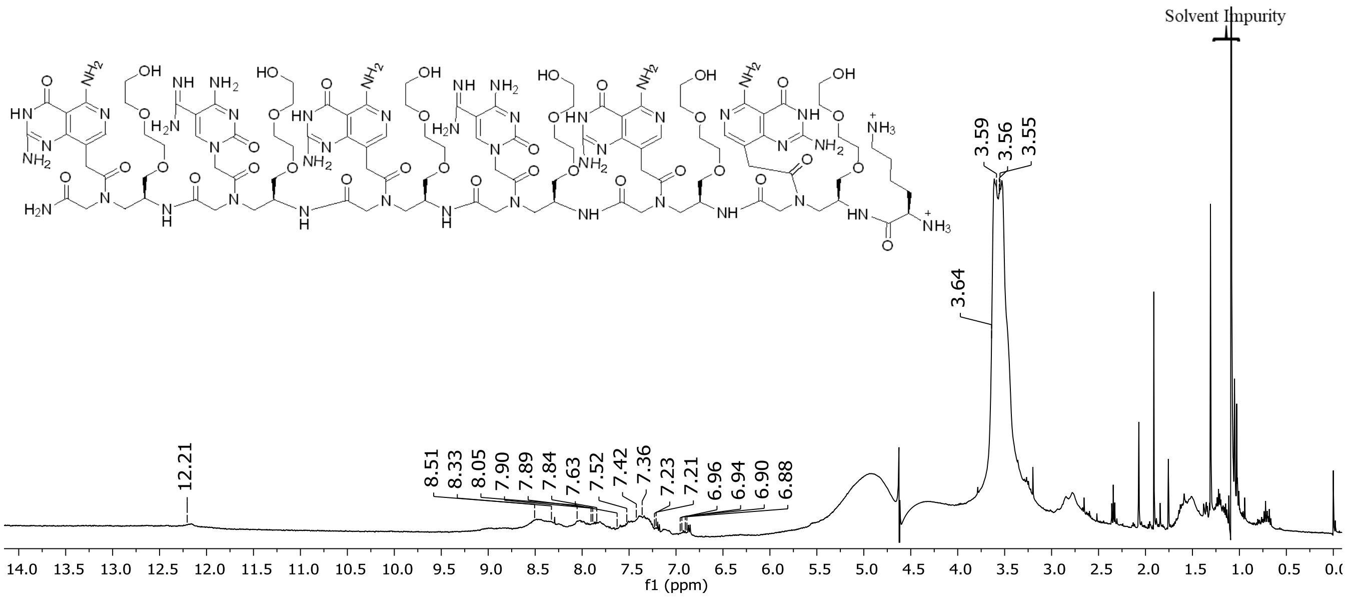

Supplementary Figure 18: ^{1}H-NMR (Watergate p3919gp Exp., 500.13 MHz, 90%H_2O:10%D_2O, 9K scans) spectrum of P1 at 25 °C. The concentration of P1 was 0.5 mM, prepared in 1xPBS buffer containing H_2O/D_2O at a 9:1 volume-ratio. The sample was annealed at 95 °C and gradually cooled down to room temperature before acquisition of the ^{1}H-NMR data. The signal strengths were low at 0.5 mM concentration due to the presence of several rotameric structures (36 possible rotamers) of P1. In the ^{1}H NMR spectrum of P1, the weak intensity with broadness in signal at 12.21 ppm indicated that the P1-P1 duplex was formed but very weak even at such a high concentration (500 µM). The weak broad signal observed in the ^{1}H NMR could be due to the P1-P1 duplex formed by base-base stacking of Janus base E and F or the presence of a number of intra hydrogen bonding centers of P1, which may have resulted in a broad signal between 10.0-15.0 ppm. The signal intensity for each proton in P1 was very weak and broad due to the presence of several (approximate 36) rotamers in the solution form. However, all rotamers were converted to the single isomer when they hybridized with DNA (Sharp signals in ^{1}H NMR).

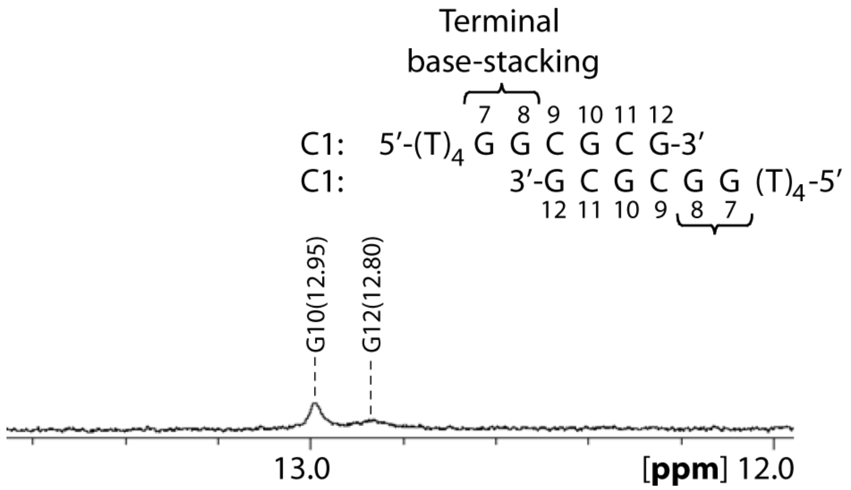

Supplementary Figure 19: Imino proton signals of a partial C1-C1 duplex.

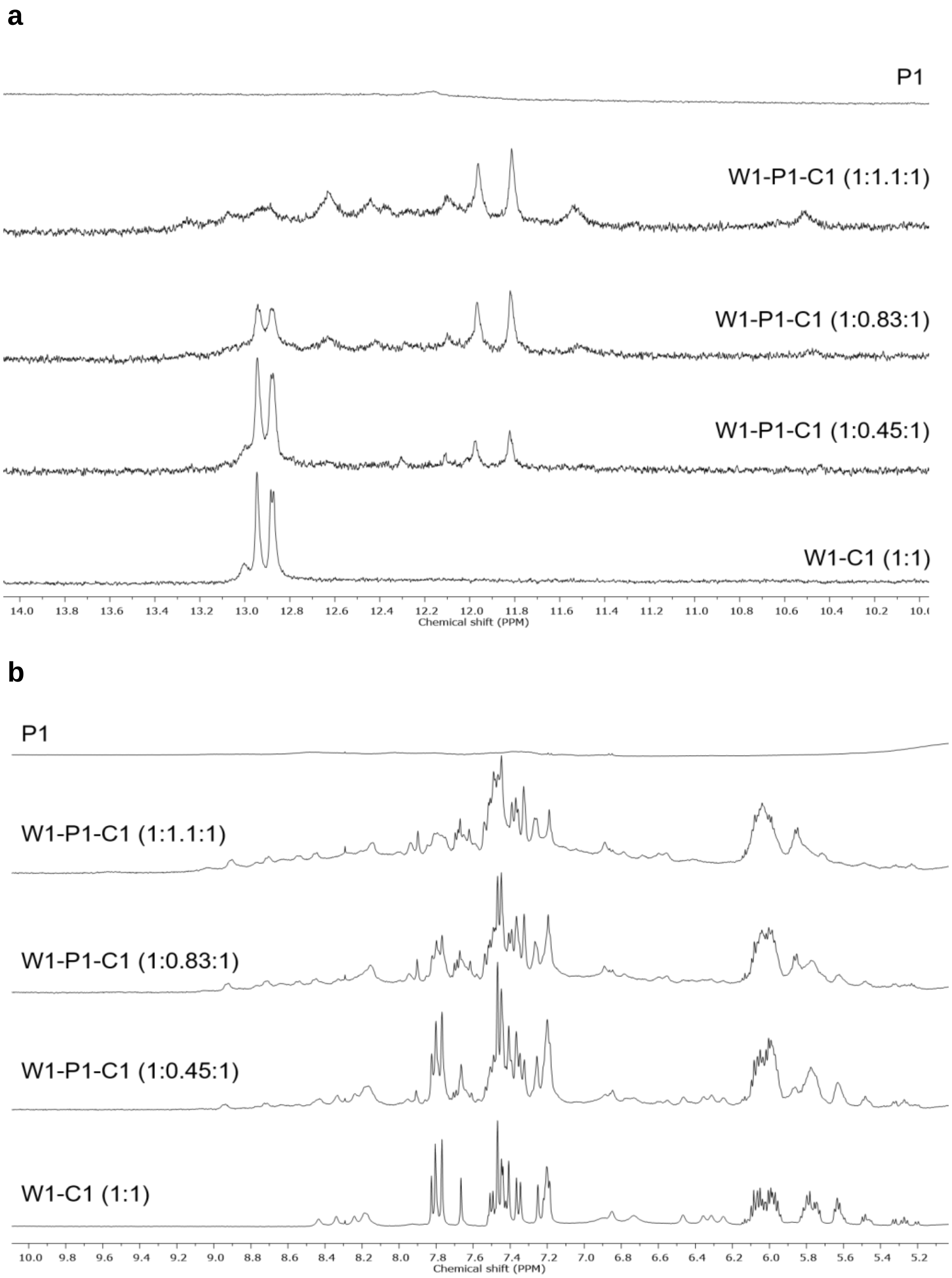

Supplementary Figure 20: ^{1}H-NMR (Watergate p3919gp Exp., 500.13 MHz, 90%H_2O:10%D_2O, 1K scans) spectrum of W1-P1-C1 at 25 °C and with different concentrations of P1. (**a**) and (**b**) are the imino and aromatic regions of triplex W1-P1-C1, respectively. The concentrations of W1 and C1 were 0.5 mM each, prepared in 1xPBS buffer containing H_2O/D_2O at a 9:1 volume-ratio. The concentration of P1 was 2.2 mM prepared in 40 µL of 1xPBS buffer containing H_2O/D_2O at a 9:1 volume-ratio and after each addition (15 (1:0.45:1), 15 (1:0.83:1), and 10 µL (1:1.1:1)) the samples were incubated for 30 min at 37 °C. The final concentration of W1, C1, and P1 were 0.40, 0.40, and 0.44 mM respectively. (NMR Region 5.0-14.0 ppm). Several new peaks were observed in 10.0-14.0 ppm which confirmed the formation of a triplex W1-P1-C1. However, the peak broadening indicated the interaction between P1 and C1 is weak compared to that of P1 with W1.

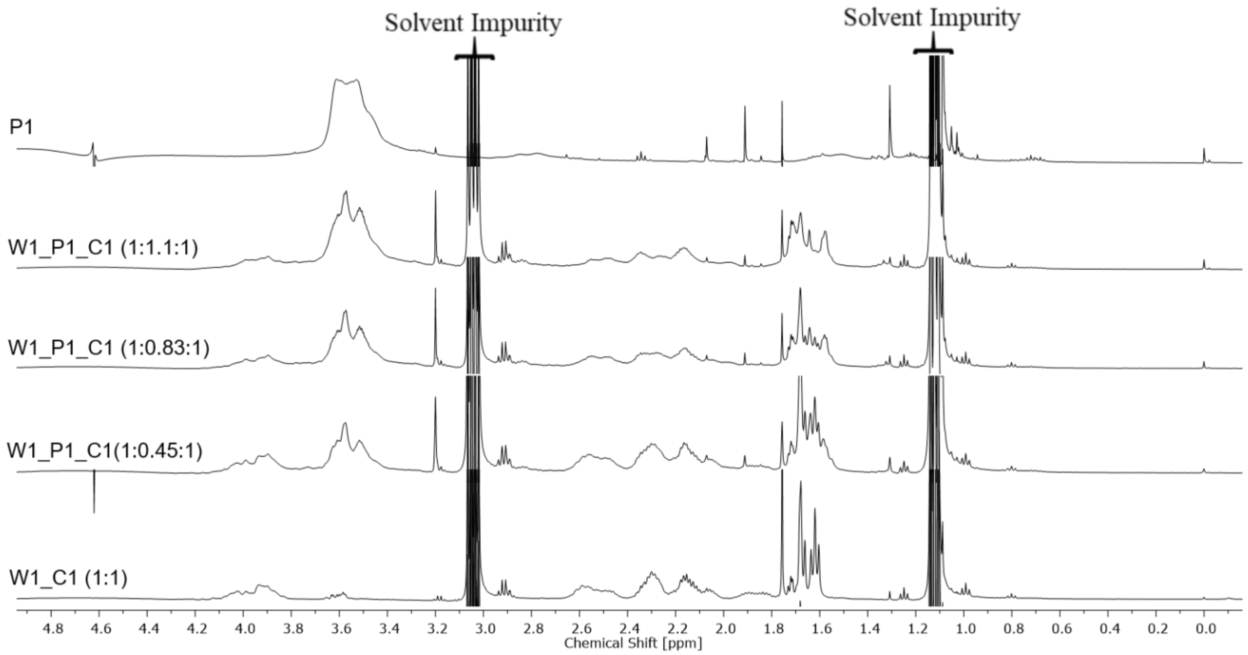

Supplementary Figure 21: The same dataset as that shown in **Fig.S20**, with the 0.0-5.0 ppm regions expanded.

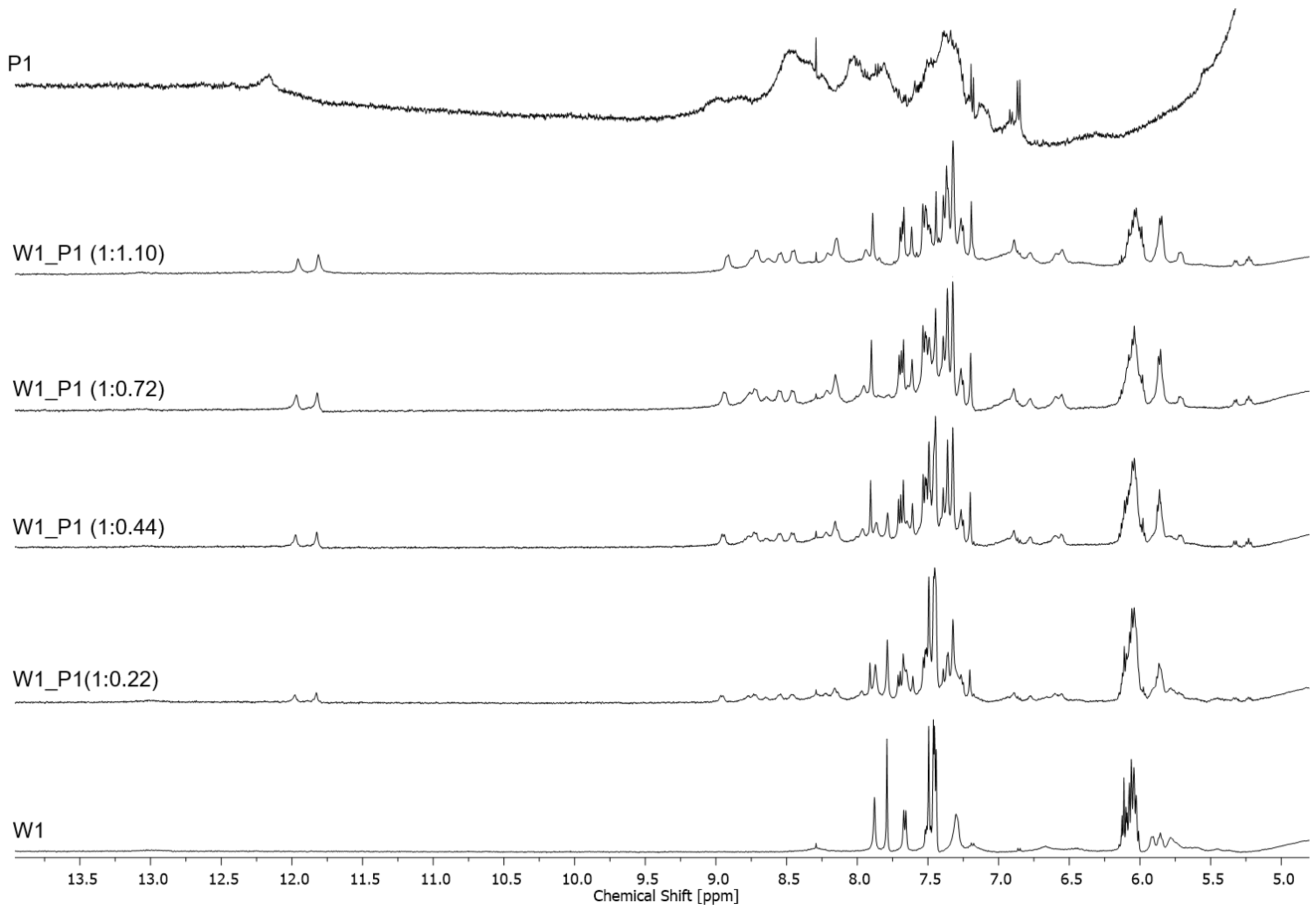

Supplementary Figure 22: ^{1}H-NMR (Watergate p3919gp Exp., 500.13 MHz, 90%H_2O:10%D_2O, 1K scans) spectrum of W1-P1 with different concentrations of P1. The initial concentration of W1 was 0.5 mM, prepared in 1xPBS buffer containing H_2O/D_2O at a 9:1 volume-ratio. The concentration of P1 was 2.2 mM in 40 µL prepared in 1xPBS buffer containing H_2O/D_2O at a 9:1 volume ratio and after each addition (8 (1:0.22), 8 (1:0.44), 10 (1:0.72), 14 (1:1.10) µL) the samples were incubated for 30 min at 37 °C. After last addition of P1, the new peaks were identified in aromatic and amine region which could be possibly from P1 due to P1 occupied helical structure with W1 instead of several rotamers. The final concentrations of W1 and P1 were 0.40 and 0.44 mM, respectively. (NMR region: 5.0-14.0 ppm).

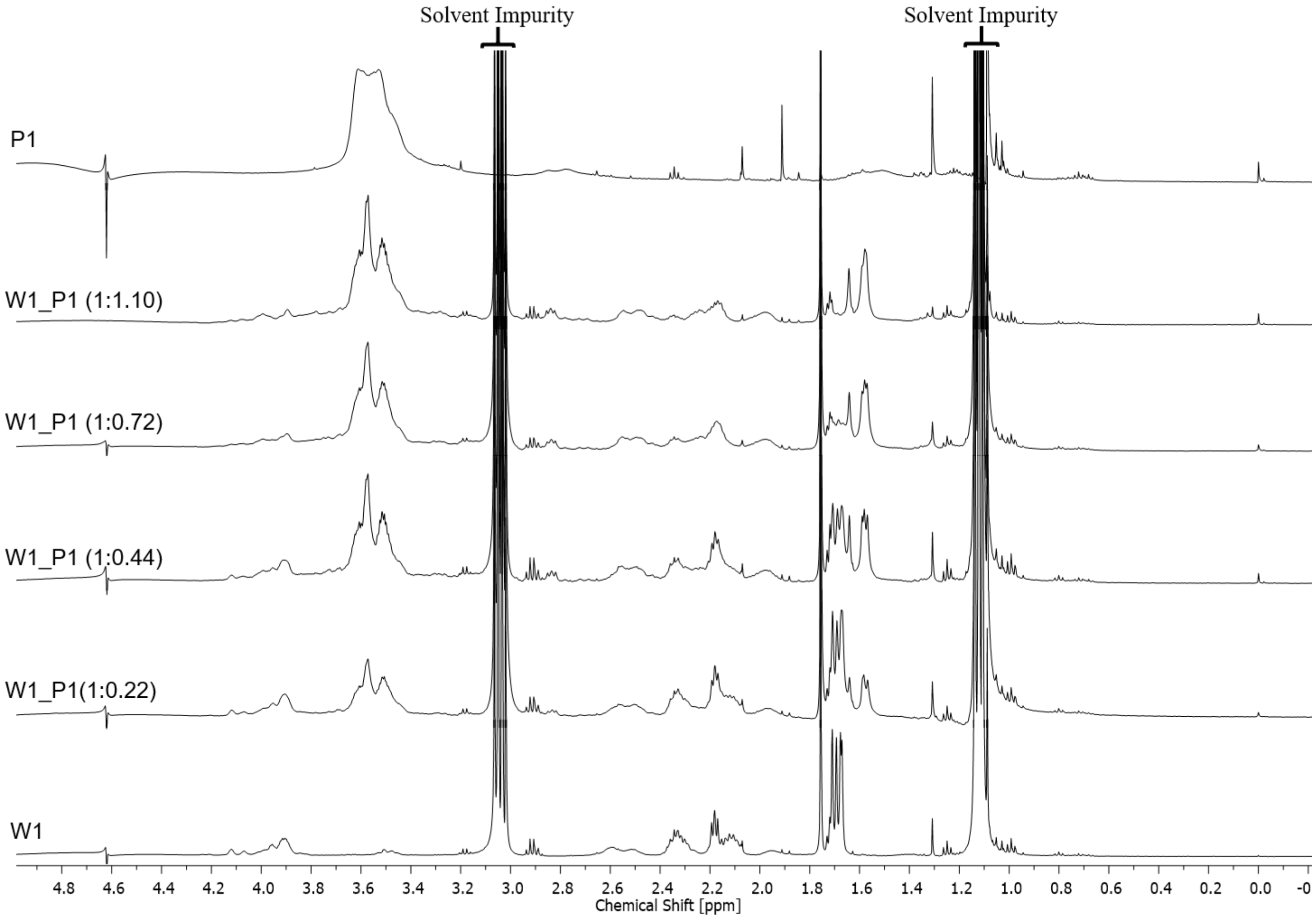

Supplementary Figure 23: The same dataset as that shown in **Supplementary Figure 22**, with the 0.0-5.0 ppm regions expanded.

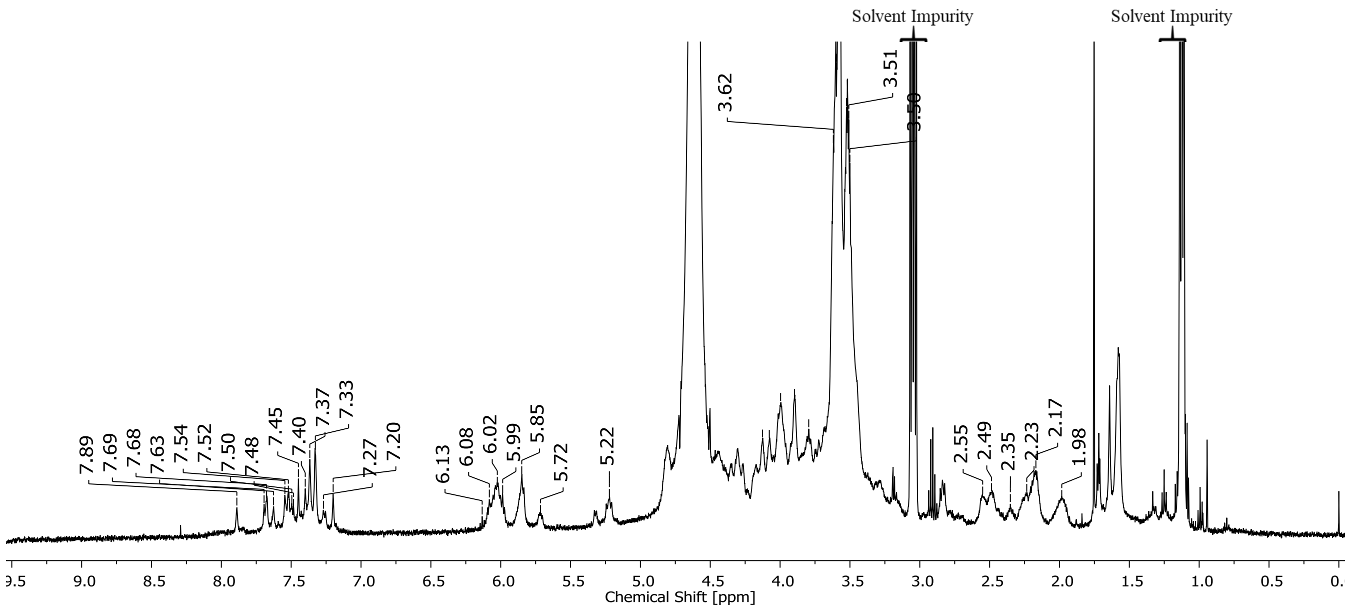

Supplementary Figure 24: ^{1}H-NMR (500.13 MHz, 100%D_2O, 512 scans) spectrum of W1-P1 at 25 °C. The concentrations of W1 and P1 were 0.4 and 0.44 mM, respectively, prepared in 1xPBS buffer containing 100% D_2O.

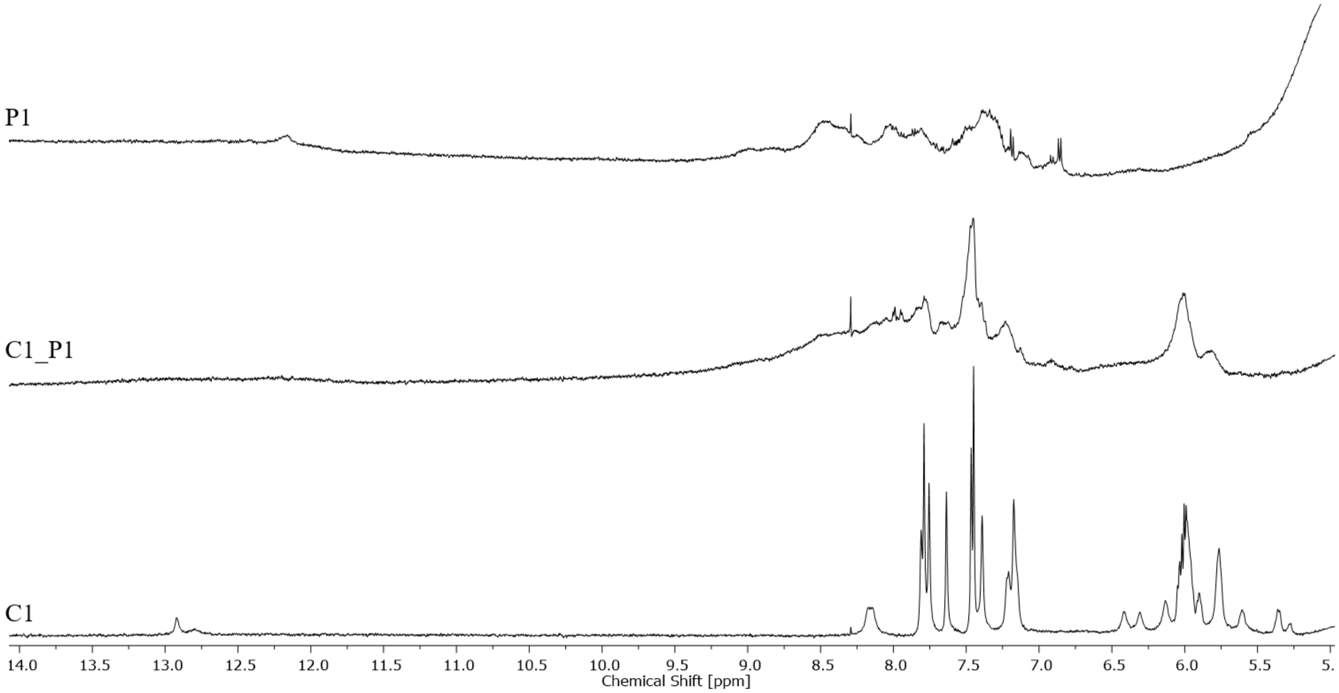

Supplementary Figure 25: ^{1}H-NMR (Watergate p3919gp Exp., 500.13 MHz, 90%H_2O:10%D_2O, 1K scans) spectrum of C1-P1 at 25 °C, prepared in 1xPBS buffer containing H_2O/D_2O at a 9:1 volume-ratio. The broaden peak in imino region as well as in aromatic and amine region indicating P1-C1 has very weak interaction. However, with this weak interaction the complex remains stable in duplex form (See EMSA result in the manuscript).

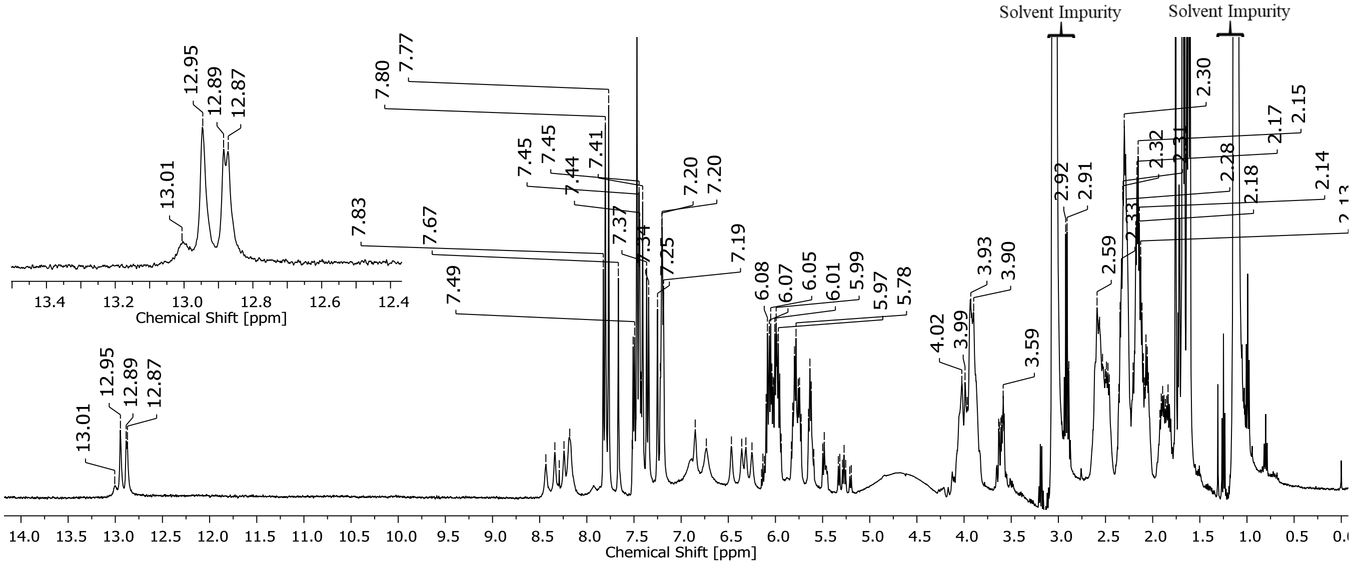

Supplementary Figure 26: ^{1}H-NMR (Watergate p3919gp Exp., 500.13 MHz, 90%H_2O:10%D_2O, 1K scans) spectrum of W1-C1 at 25 °C. The final concentrations of W1 and C1 were 0.5 mM each, prepared in 1xPBS buffer containing H_2O/D_2O at a 9:1 volume-ratio. From this NMR, we observed four different types imino peaks, and approximately 12-15 different types of amino proton peaks from the six-nucleobase pairs.

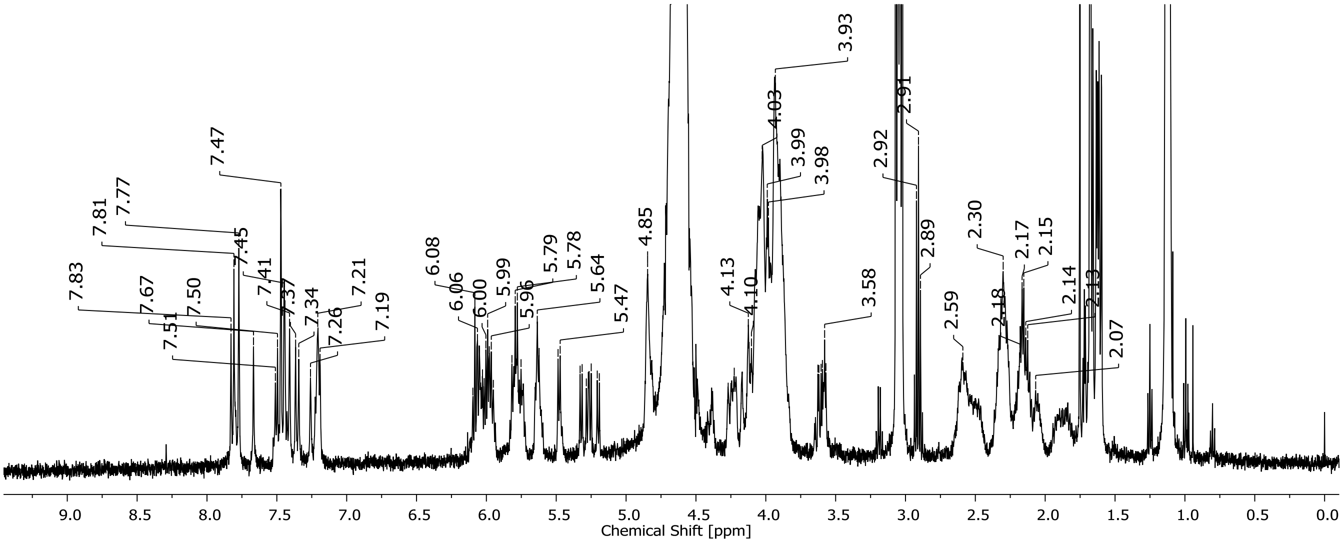

Supplementary Figure 27: ^{1}H-NMR (500.13 MHz, 100%D_2O, 128 scans) spectrum of W1-C1 at 25 °C. The final concentration of W1 and C1 were 0.5 mM each, prepared in 1xPBS buffer containing 100% D_2O. All the exchangeable protons disappeared in 100% D_2O.

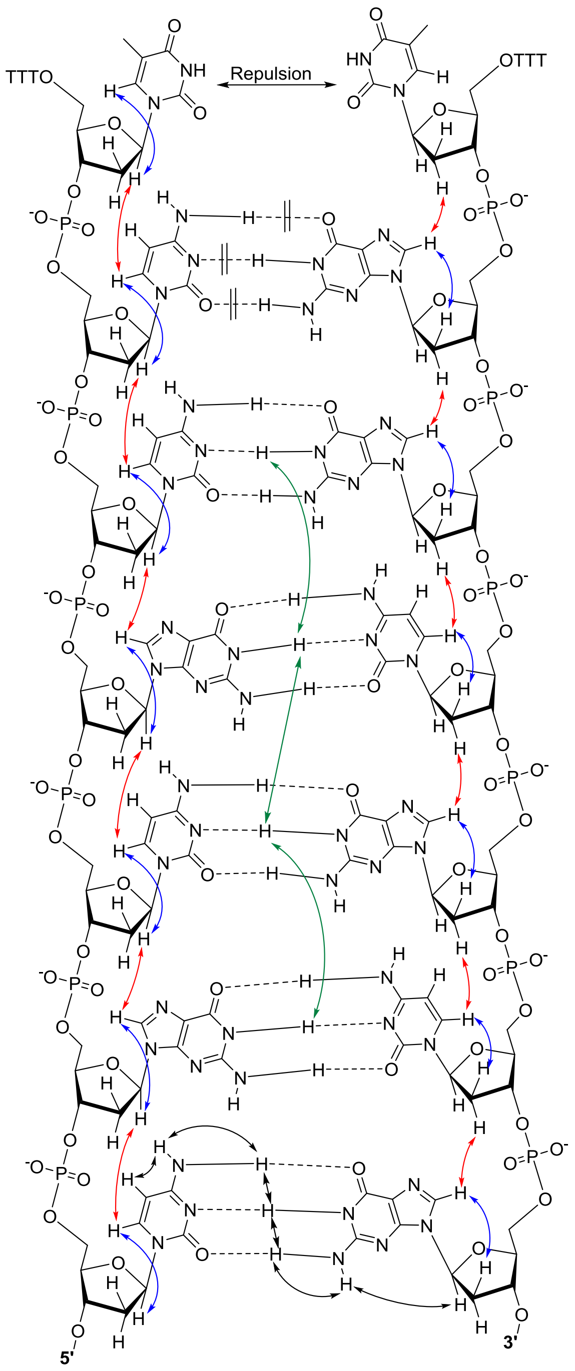

Supplementary Figure 28: 2D structure of W1-C1. Only H1', H2', and H2" protons of the deoxyribose are shown. Possible NOE space correlation for [H1'-B-(H1'+1), Direction (5'→3')], [H2', H2"-B-(H2', H2"+1), Direction (5'→3')], [B-B, Direction: 3'→5')], [Imino-Imino], [Imino-Amino-Aromatic/Anomeric protons]. C6 and G7 were breathing due to TT mismatch base-pair next to it. Red, blue, black, and green colors with arrow heads represent inter, intra, intra/inter, and inter correlation of aromatic base protons to H1', imino to amino to and imino to imino protons, respectively. All possible assignments were made from the COSY and NOESY spectra of W1-C1.

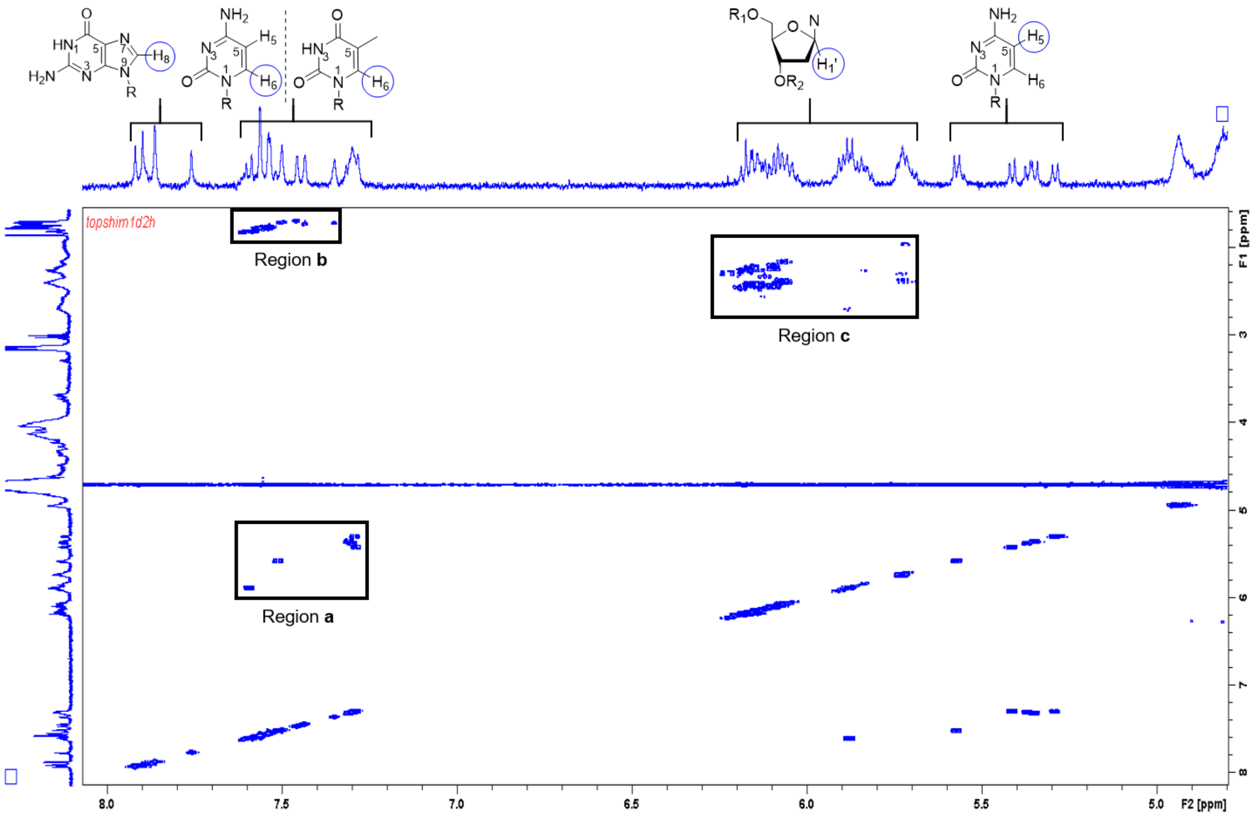

Supplementary Figure 29: COSY spectrum of W1-C1. W1-C1 duplex was prepared in 1xPBS buffer containing 100% D_2O and annealed at 90 °C for 5 min, followed by a gradual cooling to room temperature before recording the NMR data. The COSY spectrum was recorded using cosygpprqf as a pulse program at 25 °C on 500 MHz NMR instrument. This COSY spectrum was used to measure the *J*-coupled protons within each nucleotide and deoxyribose. This is a first stage assignment for the cytosine H5 and H6 protons (*J*= ~ 7.00 Hz), thymine H6/5-Me (*J*= ~ 1-2 Hz), and anomeric H1' to H2', H2" which showed cross peaks individually in region **a**, **b**, and **c** respectively. Using this information, the scalar (*J*) coupling correlation network should be identified based on the sequence of W1-C1. However, identification of H3', H4', H5', and H5" were difficult due to resonance overlap, merging with water signal, and single intensities of W1-C1.

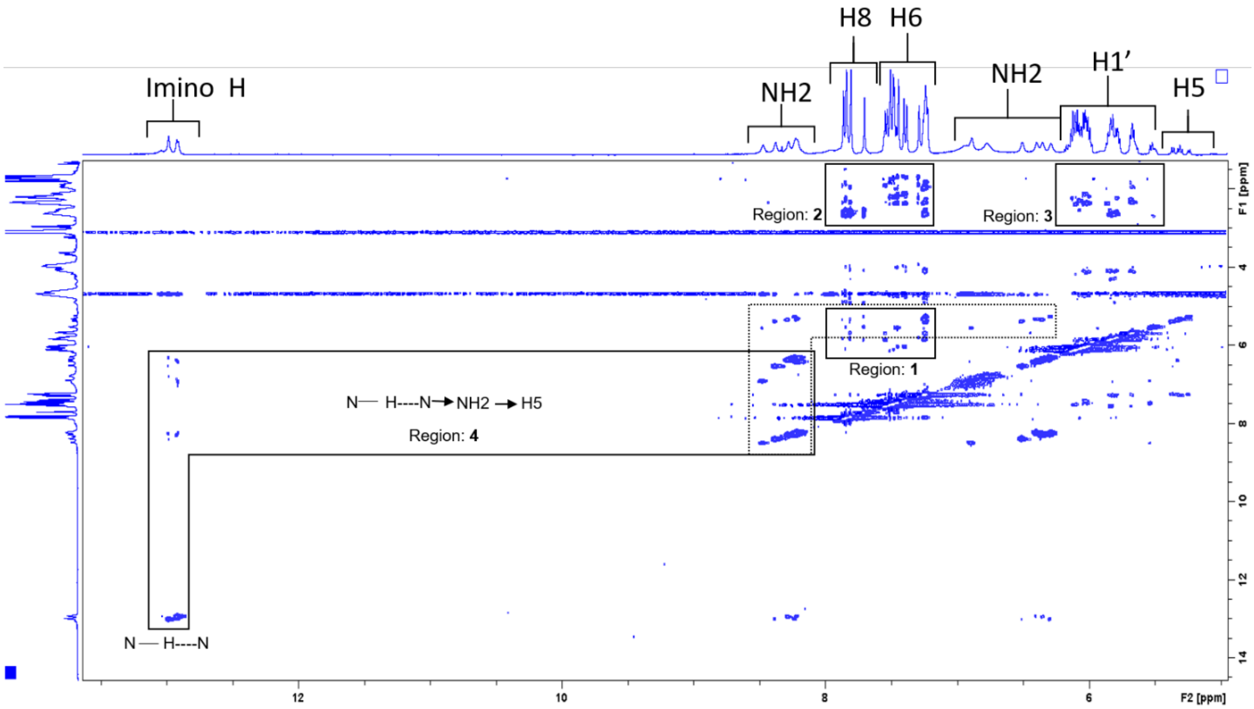

Supplementary Figure 30: 2D NOESY spectrum of W1-C1. W1-C1 duplex were prepared in 1xPBS containing 90% H_2O:10% D_2O and annealed at 90 °C for 5 min, followed by a gradual cooling to room temperature before recording the NMR data. The NOESY spectrum was recorded using noesygpph19 (Watergate NOESY) as a pulse program and 200 ms as mixing time at 25 °C on a 500 MHz NMR instrument. This NOESY spectrum was used to measure the through space correlation between aromatic base to deoxyribose protons, base to base protons, imino to complementary strand amine protons and imino to imino protons for W1-C1. The different types of regions in the NOESY spectrum indicated the space correlation between base to inter and intra H1', H2', H2" (region 1&2), H1' to H2', H2" (region 3) and imine to imine and imino to amine to aromatic protons (region 4). The through bond and through space proton-proton correlation network was identified based on the sequence of W1-C1 using COSY and NOESY, respectively.

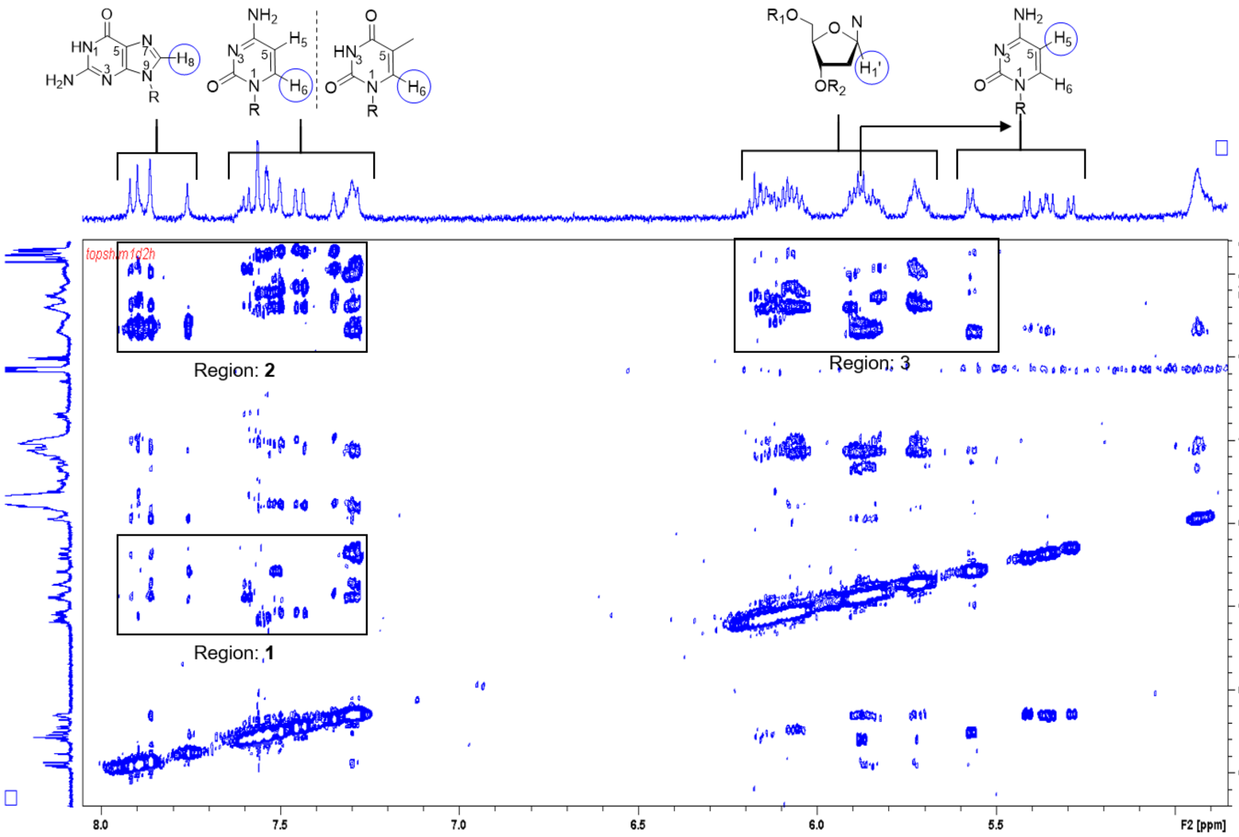

Supplementary Figure 31: 2D NOESY spectrum of W1-C1. W1-C1 duplex was prepared in 1xPBS containing 100% D_2O and annealed at 90 °C for 5 min, followed by a gradual cooling to room temperature before recording the NMR data. The NOESY spectrum was recorded using noesygpph19 (Watergate NOESY) as a pulse program 200 ms as a mixing time at 25 °C on 500 MHz NMR instrument. Since the spectrum was collected in 100% D_2O, all exchangeable protons disappeared in this spectrum and only aromatic and deoxyribose protons showed the NOE cross-peaks. Region 1 showed the base to H1' connectivity's in both strands of DNA. Aromatic base protons to H2' and H2'' correlations were identified in Region 2. In Region 3, the 2-deoxyribose H1' to H2 and H2'' connectives were identified.

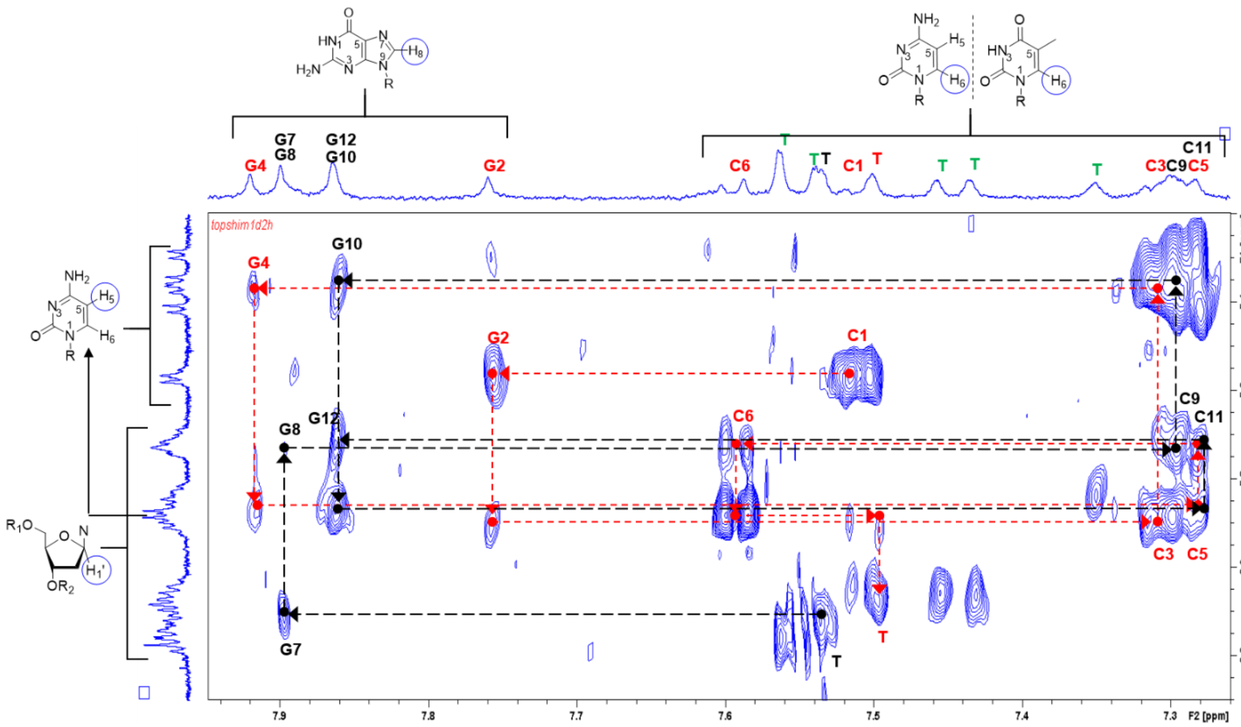

Supplementary Figure 32: Expanded Region 1 of 2D NOESY spectrum to assign the non-exchangeable protons for base and deoxyribose. The NOESY spectrum showed the base to deoxyribose through anomeric proton correlation for both strands of W1-C1. Red dashed line represents the correlation between aromatic base protons to H1' to aromatic base protons of Watson strand in W1-C1. Black dashed line presents the correlation between aromatic base protons to H1' to aromatic base protons for Crick strand in W1-C1.

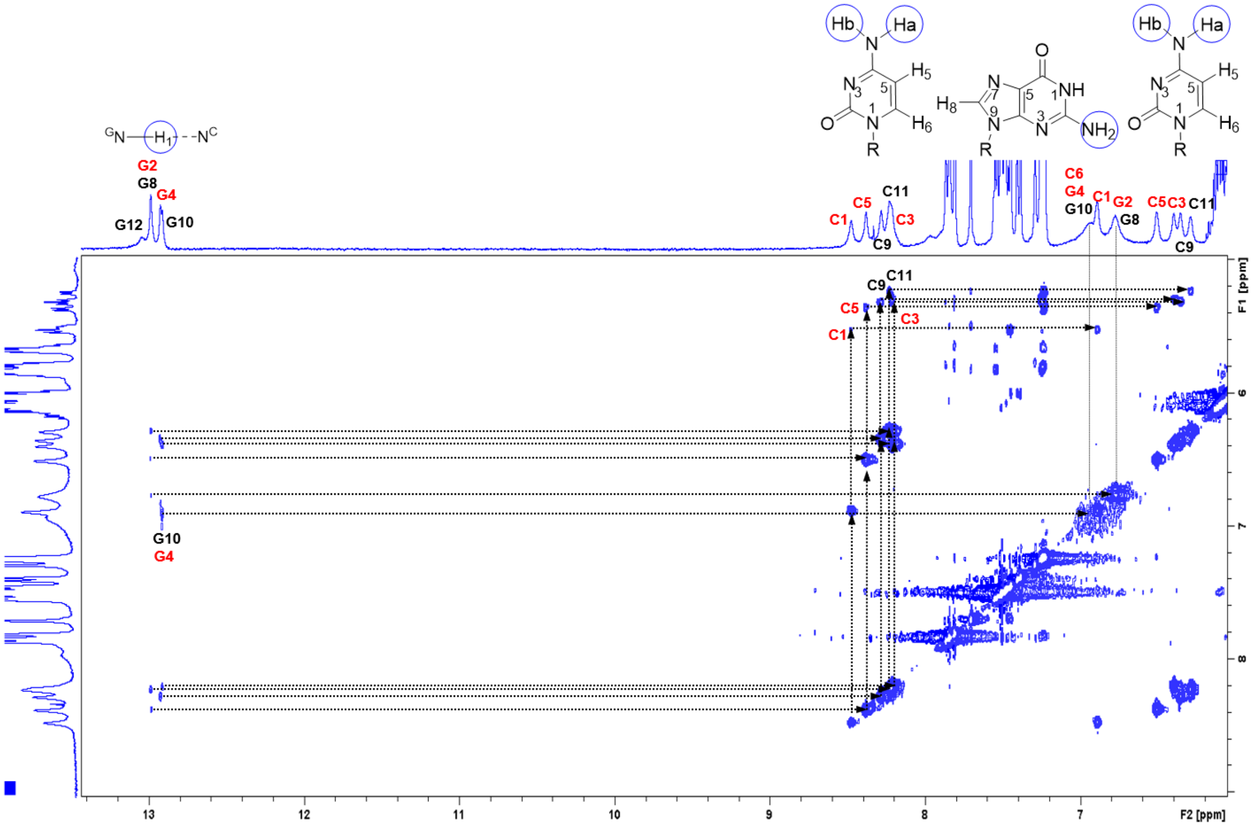

Supplementary Figure 33: Expanded Region 4 of the 2D NOESY spectrum to assign the exchangeable protons of base. The NOESY spectrum showed the imino to amino to aromatic protons correlation for both strands of W1-C1

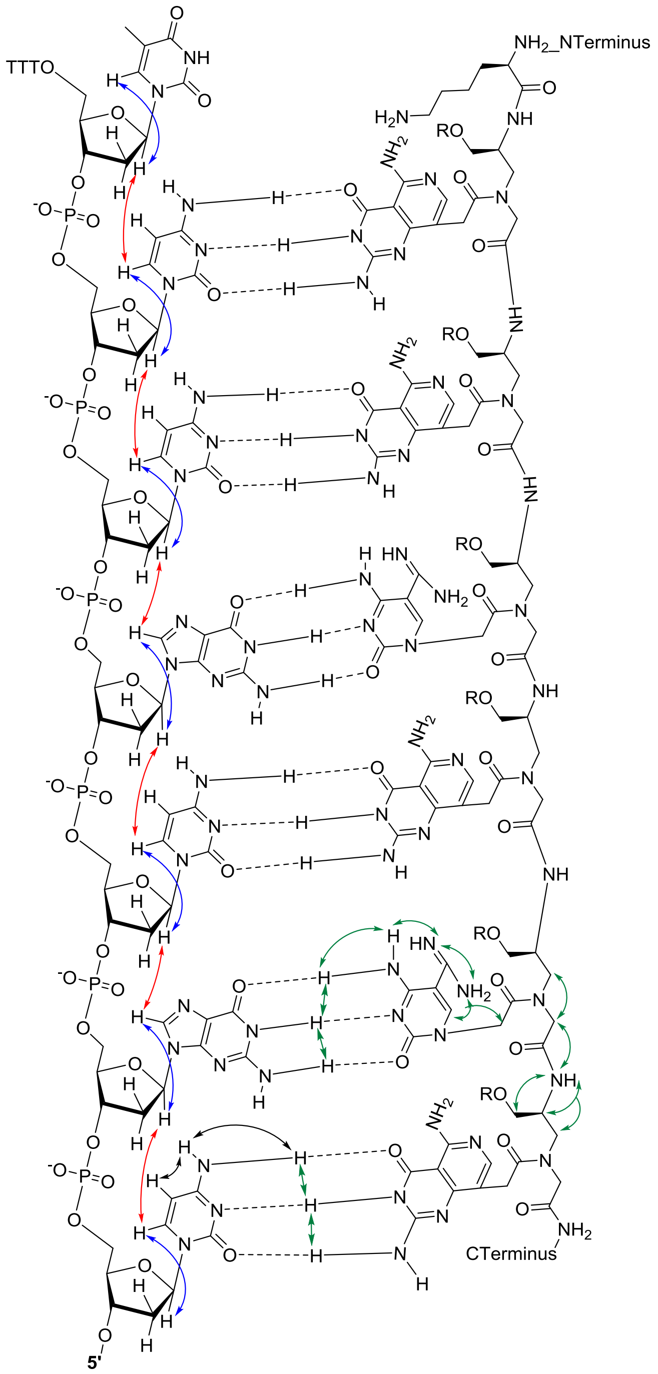

Supplementary Figure 34: 2D structure of W1-P1. Possible NOE space correlations for [H1'-B-(H1'+1), Direction (5'→3')], [H2', H2"-B-(H2', H2"+1), Direction (5'→3')], [Imino-Imino], [Imino-Amino-Aromatic/Anomeric protons]. Red, blue, and black colors with arrow head represent correlation of aromatic base to H1', Imino to amine, amine to aromatic protons were identified from the COSY and NOESY spectra. However, the green colored correlation is not yet assigned due to overlapping and weak signals of Janus base amine with aromatic protons and amide protons.

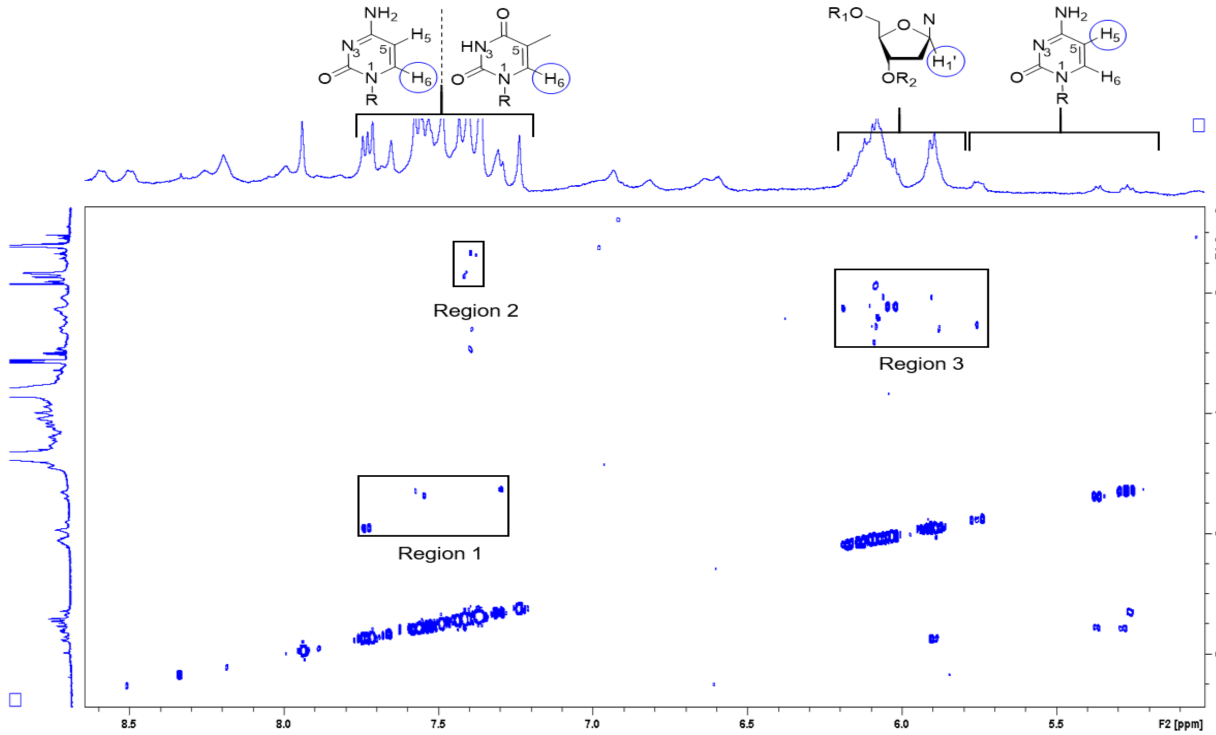

Supplementary Figure 35: COSY spectrum of the W1-P1. W1-P1 duplex was prepared in 1xPBS containing 90% H_2O:10% D_2O. P1 was added to the pre-annealed W1 and incubated at 37 °C for 4 h prior to recording the NMR data. The COSY spectrum was recorded using cosygpprqf (COSY Presat) as a pulse program at 25 °C on 500 MHz NMR instrument. This COSY spectrum was used to measure the *J*-coupled protons within each nucleotide, backbone, and deoxyribose. This is the first stage assignment for the cytosine H5 and H6 protons (*J* = ~ 7.00 Hz), thymine H6/5-Me (*J* = ~ 1-2 Hz), and Lysine, and anomeric H1' to H2', H2" which showed the cross peaks individually in Region **1**, **2**, and **3** respectively. The mini-PEG backbone CH and CH2 were shown as a broad single peak in NMR between 3.25-3.75 ppm. Using this information, the scalar (*J*) coupling correlation network could be identified based on sequence of W1-P1. However, identification of H3', H4', H5' and H5" and mini-PEG backbone were difficult due to resonance overlap, merging with water signal, and low concentration of W1-P1.

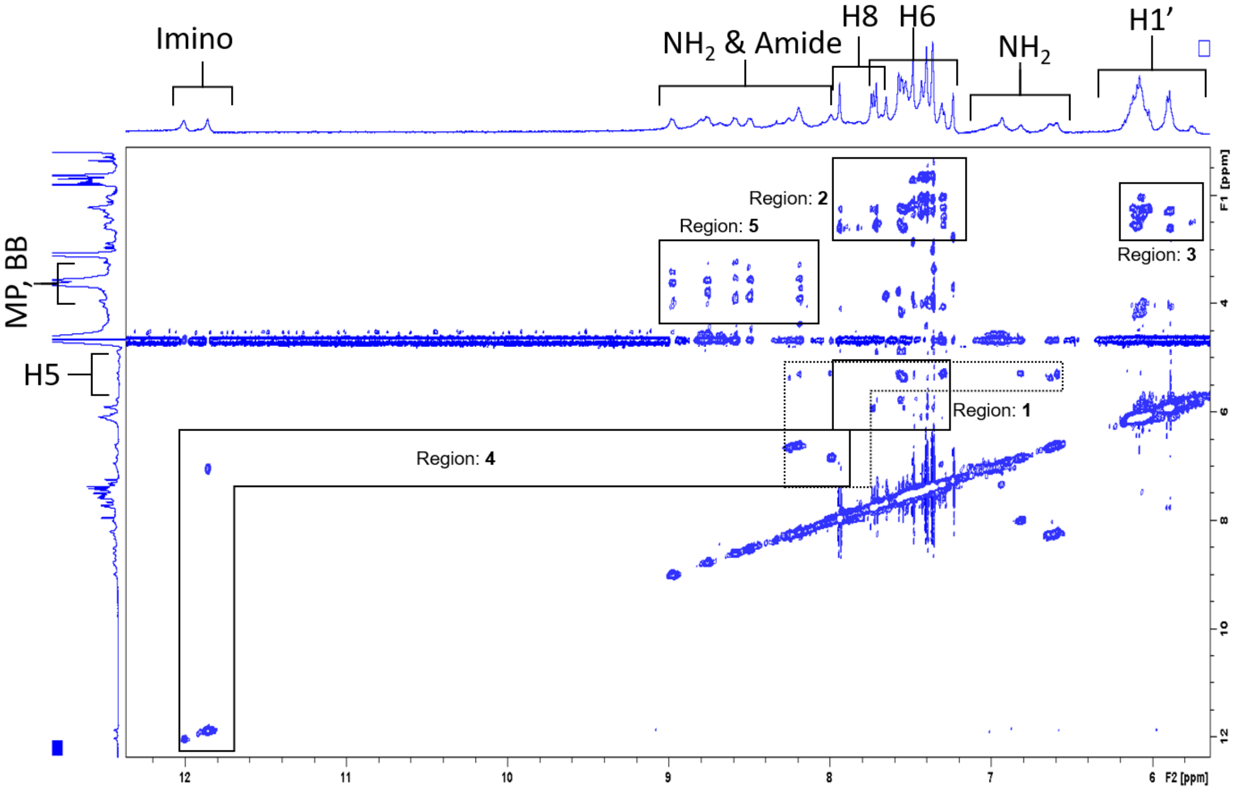

Supplementary Figure 36: 2D NOESY spectrum of W1-P1. W1-P1 duplex was prepared in 1xPBS containing 90% H_2O:10% D_2O. P1 was added to the pre-annealed W1 and incubated at 37 °C for 4 h prior to recording the NMR data. The NOESY spectrum was recorded using noesygpph19 (Watergate NOESY) as a pulse program and 200 ms as a mixing time at 25 °C on 500 MHz NMR instrument. The different types of regions in NOE indicated the space correlation between aromatic base proton to inter and intra H1', H2', H2" (region 1&2), H1' to H2', H2" (region 3), imine to amine proton (region 4) and amide protons to MP side chain and CH and CH2 from PNA backbone (Region 5).

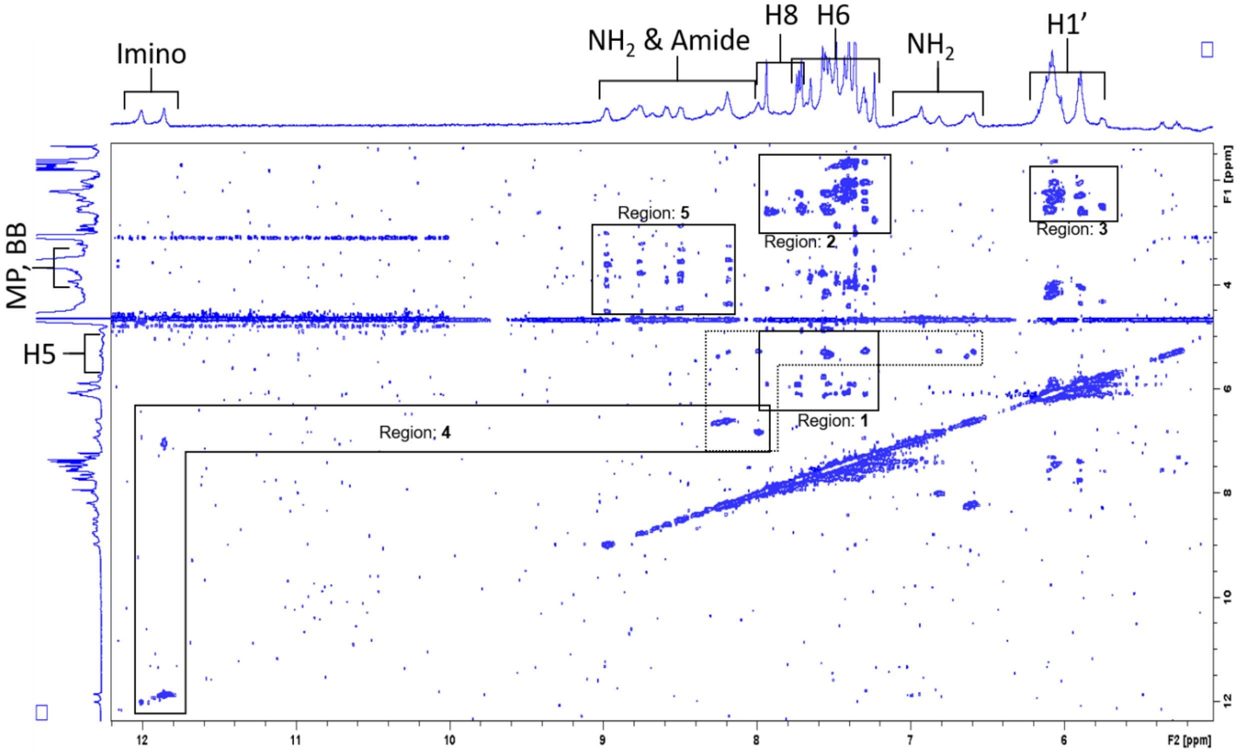

Supplementary Figure 37: 2D NOESY spectrum of W1-P1. W1-P1 duplex was prepared in 1X PBS containing 90% H_2O:10% D_2O. P1 was added to the pre-annealed W1 and incubated at 37 °C for 4 h prior to recording the NMR data. The NOESY spectrum was recorded using noesygpph19 as a pulse program and 300 ms as a mixing time at 25 °C on 500 MHz NMR instrument. This NOESY spectrum was used to measure the space correlation between aromatic base to deoxyribose, Aromatic base to aromatic base, imino to complementary strand amine and imino to imino protons for W1-P1. The different types of regions in NOESY spectrum indicated the space correlation between aromatic base protons to inter and intra H1', H2', H2" (region 1&2), H1' to H2', H2" (region 3), imine to amine (region 4) and amide to mini-PEG side chain and CH and CH2 from PNA backbone (Region 5).

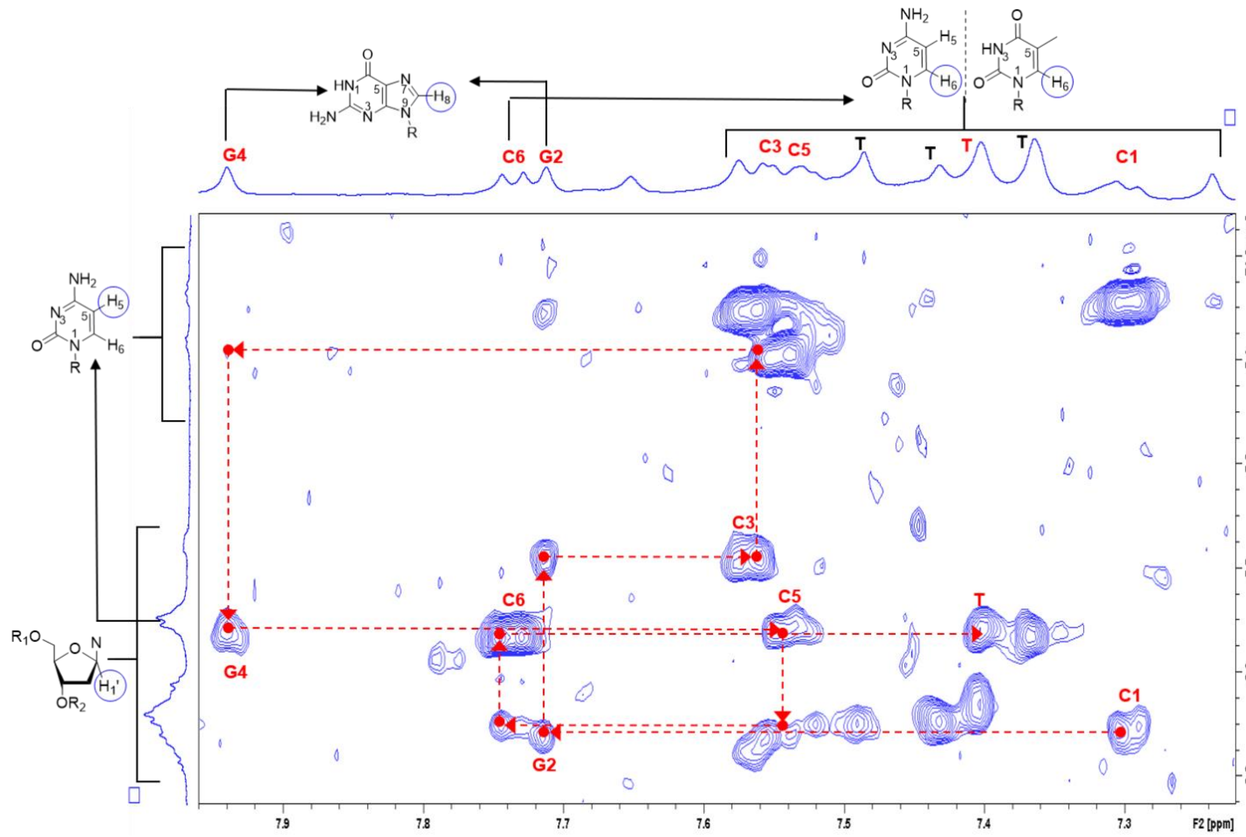

Supplementary Figure 38: Expanded Region 1 of NOESY spectrum to assign the non-exchangeable protons for nucleobase and deoxyribose. The NOESY spectrum showed the nucleobase to deoxyribose through anomeric proton correlation for W1-P1.

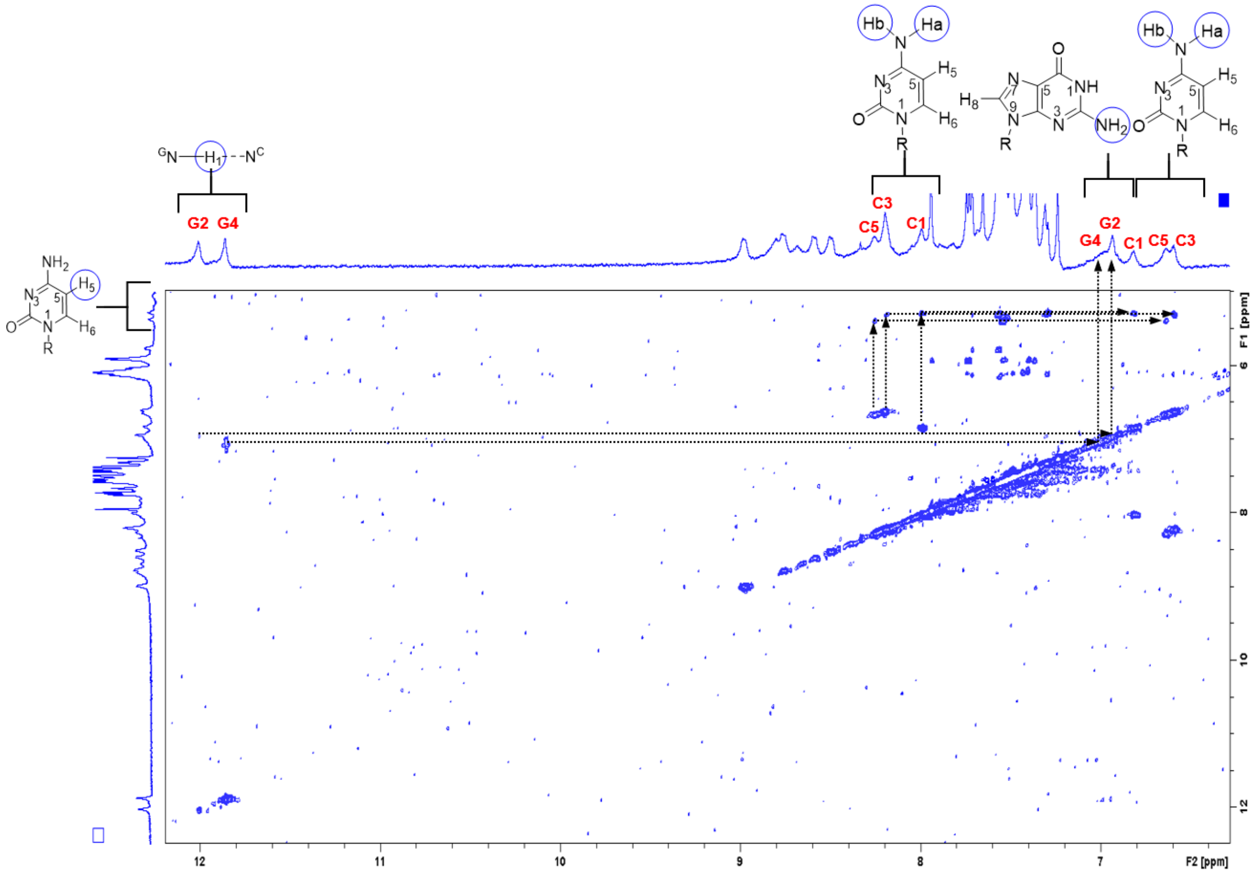

Supplementary Figure 39: Expanded Region 4 of 2D NOESY spectrum (W1-P1) to assign the exchangeable protons of base. The NOESY spectrum showed the imino to amino protons correlation for guanine and amino to aromatic protons for cytosine for W1. However, at this stage, assignment from imino to amino or aromatic protons to P1 was not possible due to weak signal and Janus base amines were overlapped with aromatic as well as amide protons from P1.

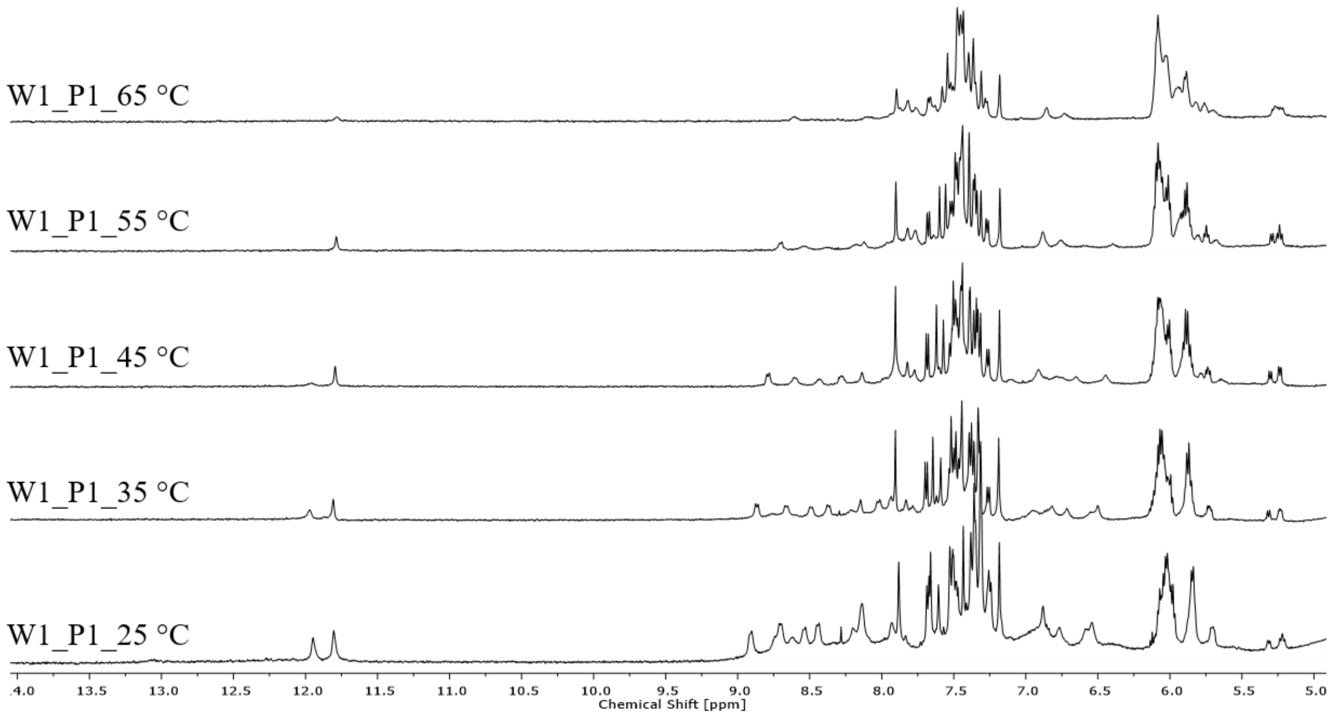

Supplementary Figure 40: ^{1}H-NMR (Watergate p3919gp Exp., 500.13 MHz, 90%H_2O:10%D_2O, 1K scans) spectrum of W1-P1 at different temperatures. The final concentrations of W1 and P1 were 0.40 and 0.44 mM, respectively, prepared in 1xPBS buffer containing H_2O/D_2O at a 9:1 volume-ratio. The imino protons at 12.95 disappeared first compared to imino protons at 12.88 ppm. Furthermore, from NOESY it confirmed that, the imino at 12.95 belongs to terminal nucleobase in DNA-PNA and 12.88 to middle portion of the DNA-PNA. Also, peaks between 8.0-9.0 and 6.5-7.1 ppm disappeared with increasing temperature and appearance of new peaks in 7.5-8.0 ppm indicated that the amino groups of both DNA and PNA has connectives through hydrogen bond with carbonyl or tertiary nitrogen in the ring.

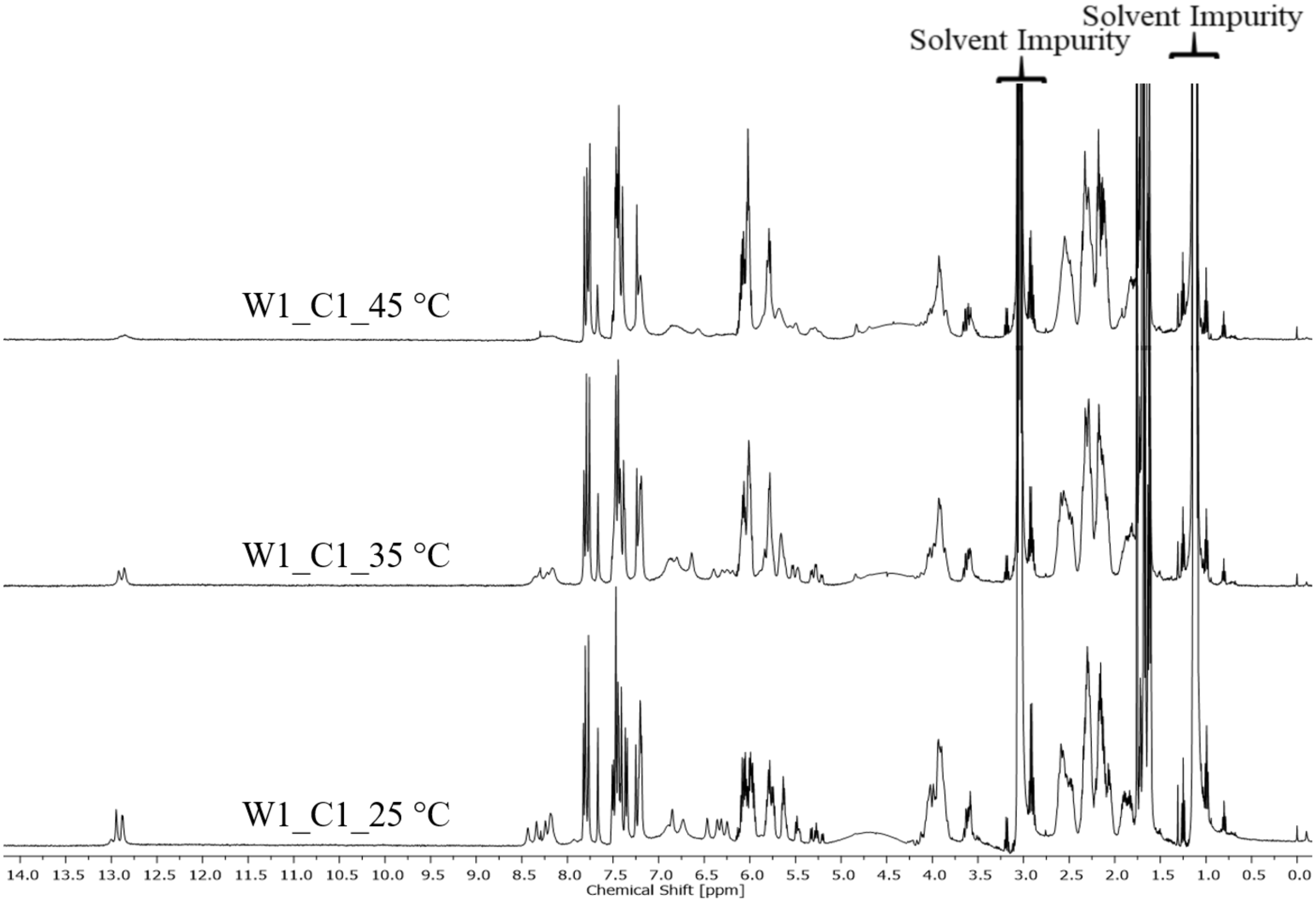

Supplementary Figure 41: ^{1}H-NMR (Watergate p3919gp Exp., 500.13 MHz, 90%H_2O:10%D_2O, 1K scans) spectrum of W1-C1 at different temperatures. The final concentrations of W1 and C1 were 0.5 mM each, prepared in 1xPBS buffer containing H_2O/D_2O at a 9:1 volume-ratio. The imino protons at 12.95 and 13.00 ppm disappeared first, followed by that at 12.88 ppm. This result further confirmed from the NOESY and COSY spectrum that, the imino proton signal at δ 13.00 belonged to G12, δ 12.95 to G2 and G8, and δ 12.89, 12.87 to G4 and G10 nucleobase respectively.

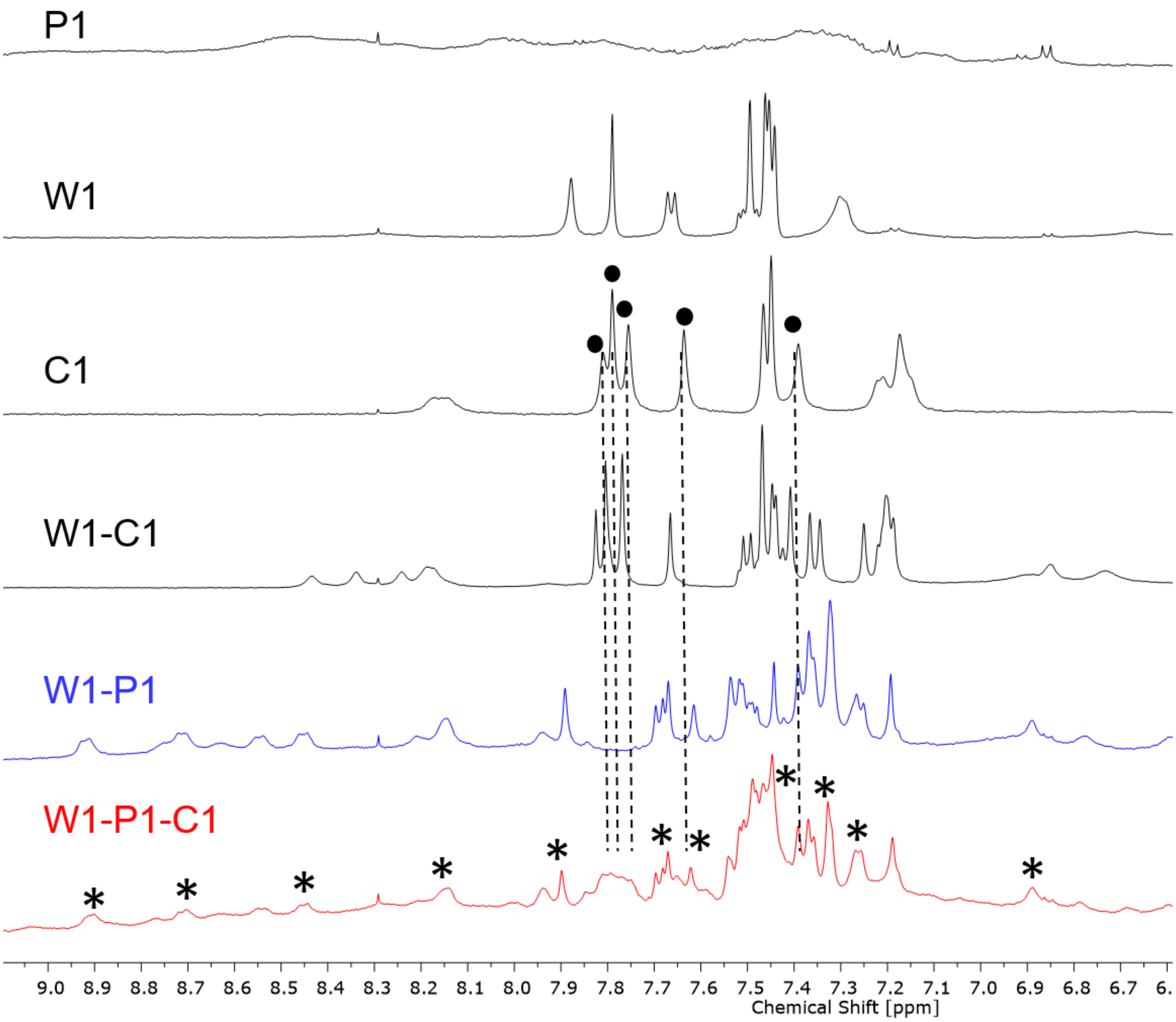

Supplementary Figure 42: ^{1}H-NMR spectra of the indicated samples (P1, W1, C1, and W1-C1 are in black solid line, W1-P1 (blue line) and W1-P1-C1 (red line)) at room temperature, where the imino proton signals of E and F and the aromatic proton signals of nucleobases (asterisks) appeared and W1 and C1 amino protons (dark circles) disappeared.

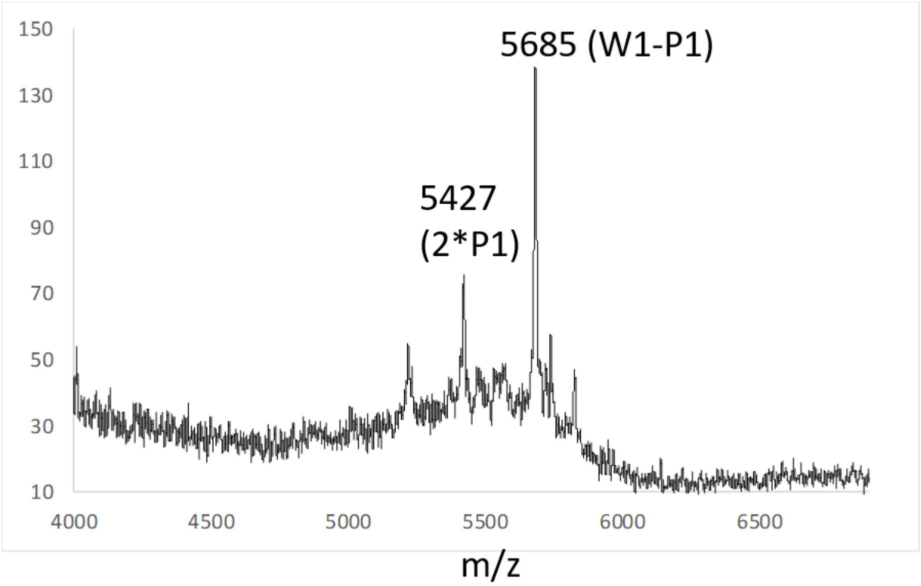

Supplementary Figure 43: MALDI-TOF MS spectrum of a W1-P1 duplex.

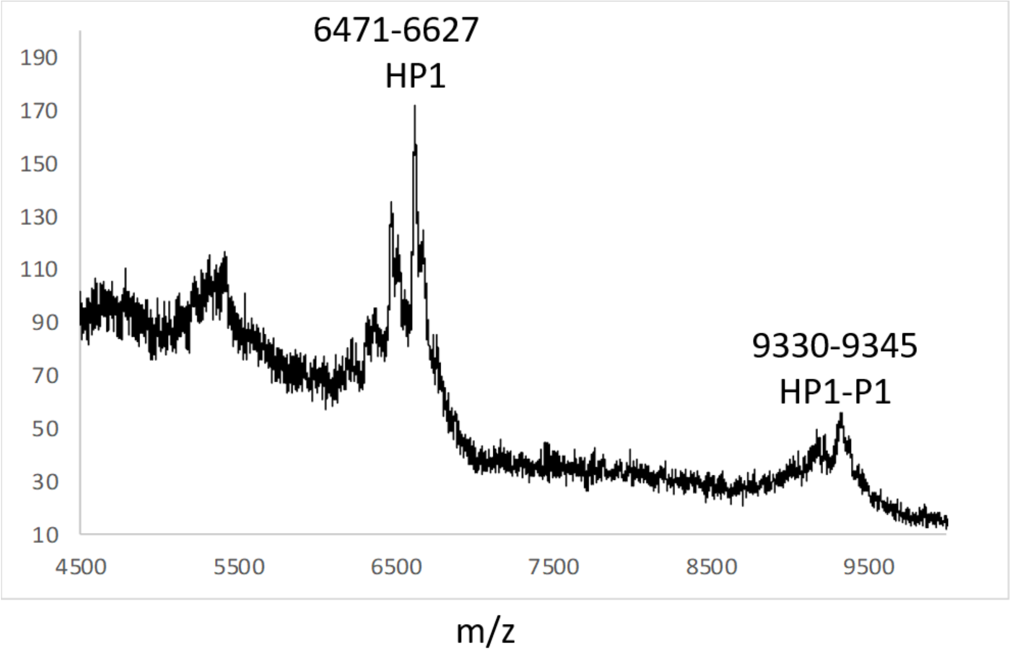

Supplementary Figure 44: MALDI-TOF MS spectrum of an HP1-P1 invasion complex.

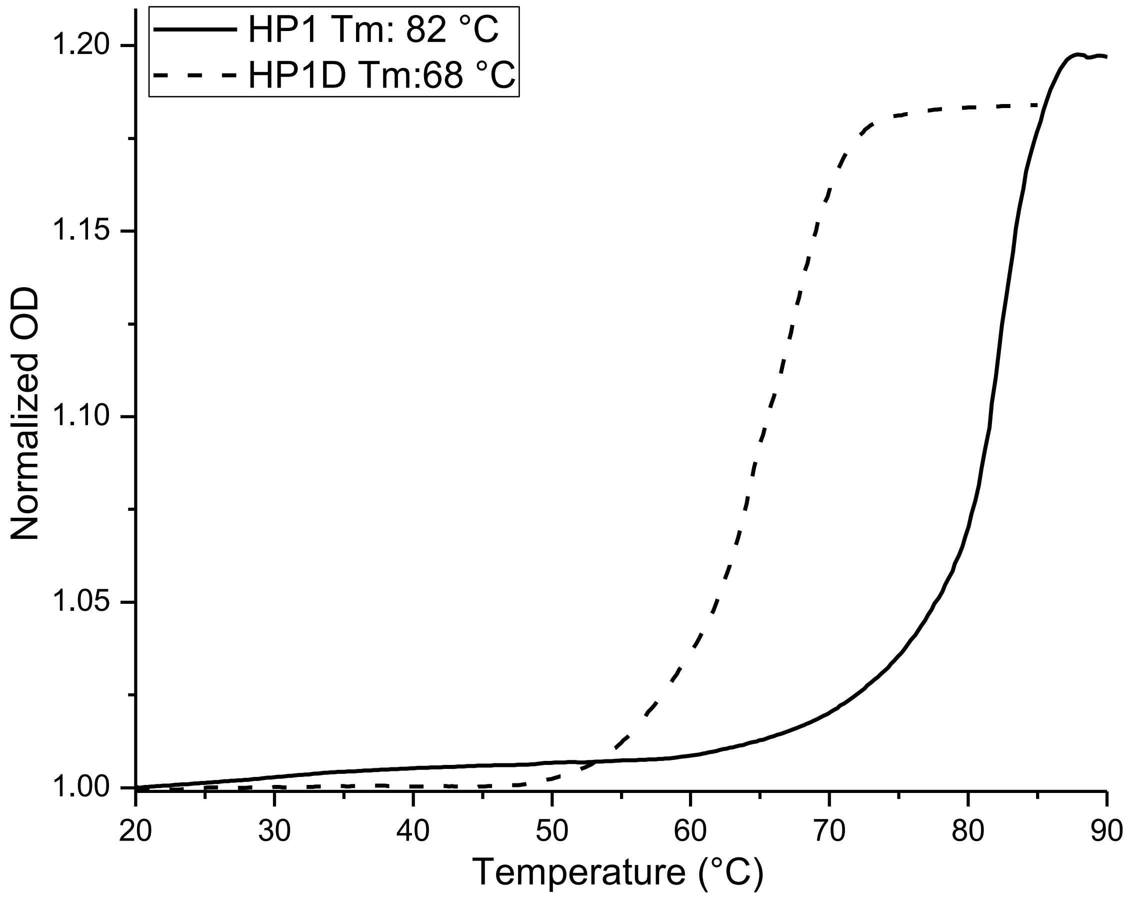

Supplementary Figure 45: UV-melting profiles of HP1 (solid line) and HP1D (dashed line). The concentration of HP1, W26, C26 strand were 10.0, 5.0, 5.0 µM, respectively. The samples were prepared by mixing the indicated strands in a PR buffer and annealed at 95 °C for 5 min, followed by a gradual cooling to room temperature before recording the UV-melting data.

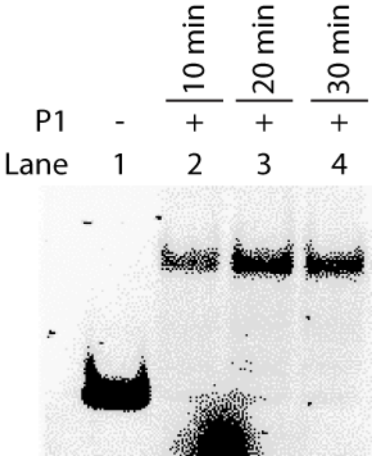

Supplementary Figure 46: Time-dependent strand invasion of HP1 by P1. The samples were prepared in a PR buffer and incubated at 37 ^{o}C for the indicated time-points. The concentrations of HP1 and P1 were 1.0 and 10.0 µM, respectively.

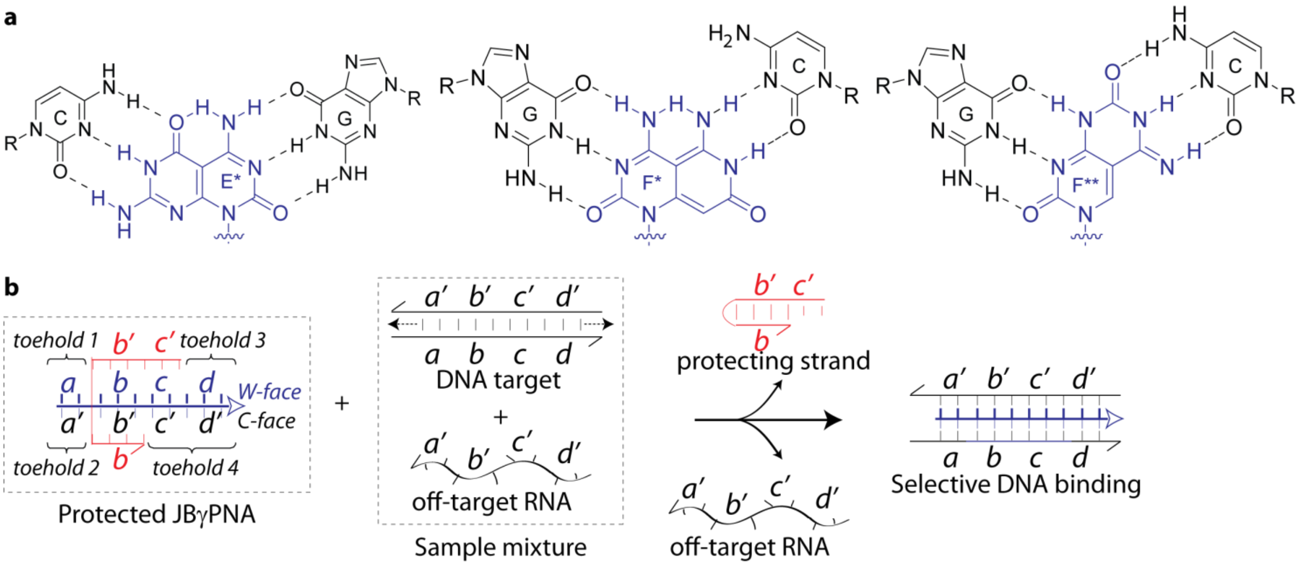

Supplementary Figure 47: Chemical structure of second-generation JBs and strategy for improving binding selectivity. (**a**) Chemical structures of second-generation E*, F*, and F** with improved hydrogen-bonding and base-stacking capabilities. (**b**) The design of a protecting probe for selective targeting double-stranded DNA (or RNA) over the single-stranded targets.

Supplementary Table 1: Atom types and charges for the PNA bases and the backbone.

Residue	Atom Name	Atom Type	Charge
BB	N3	N	-0.4336
BB	H7	H	0.2650
BB	C3	CT	0.0701
BB	H5	H1	0.1316
BB	C5	CT	-0.0419
BB	H10	HC	0.0176
BB	H8	HC	0.0176
BB	H9	HC	0.0176
BB	C2	CT	-0.1352
BB	H3	H1	0.0700
BB	H4	H1	0.0700
BB	N2	N	-0.0658
BB	C1	CT	-0.2626
BB	H1	H1	0.0972
BB	H2	H1	0.0972
BB	C6	C	0.6123
BB	O1	O	-0.5271
E	N4	N2	-0.8989
E	H1	H	0.4051
E	H2	H	0.4051
E	C1	CA	0.4964
E	N5	NA	-0.2262
E	H3	H	0.2941
E	C4	C	0.3312
E	O1	O	-0.5353
E	C3	CB	-0.0290
E	C6	CA	0.5946
E	N1	N2	-0.8993
E	H4	H	0.4082
E	H5	H	0.4082
E	N2	NC	-0.6350
E	C7	CA	0.2164
E	H6	H4	0.1165
E	N3	NC	-0.3929
E	C2	CB	0.1626
E	C5	CM	-0.2293
E	C8	CT	-0.0198
E	H7	HC	0.0553
E	H8	HC	0.0553
E	C9	C	0.4637
E	O2	O	-0.5470
B	C9	C	0.5063
B	O2	O	-0.5808
B	C8	CT	-0.0110
B	H7	HC	0.0520
B	H8	HC	0.0520
B	C1	CA	0.0125
B	C5	C	0.5341

B	O1	O	-0.5356
B	N1	NA	-0.3508
B	H2	H	0.3089
B	C4	CA	0.3879
B	N2	NC	-0.7063
B	C7	CQ	0.9210
B	N3	NC	-0.7181
B	N4	N2	-0.9263
B	H3	H	0.4019
B	H4	H	0.4019
B	C2	CA	-0.3976
B	H1	HA	0.2189
B	C3	CA	-0.1014
B	C6	CA	0.6083
B	N5	N2	-0.8396
B	H5	H	0.3809
B	H6	H	0.3809
F	O1	O	-0.6529
F	C1	C	0.8853
F	N1	NC	-0.7772
F	C5	CA	0.6600
F	C2	CM	-0.2044
F	C3	CA	0.6472
F	N4	NT	-0.9153
F	H7	H	0.3846
F	H8	H	0.3846
F	N5	N2	-0.8786
F	H6	H	0.3887
F	C4	CM	-0.0313
F	H3	H4	0.1859
F	N2	N2	-0.8208
F	H1	H	0.3928
F	H2	H	0.3928
F	N3	N*	-0.1726
F	C6	CT	-0.0425
F	H4	H1	0.0802
F	H5	H1	0.0802
F	C7	C	0.6397
F	O2	O	-0.6264
D	N2	NC	-0.6630
D	C1	CQ	0.7971
D	N1	NC	-0.5470
D	N4	N2	-0.8569
D	H3	H	0.3766
D	H4	H	0.3766
D	C4	CA	0.4841
D	N5	N2	-0.7600
D	H5	H	0.3865
D	H6	H	0.3865
D	C3	CA	-0.0422

D	C6	C	0.3493
D	O1	O	-0.5602
D	N3	NA	-0.2138
D	H1	H	0.3082
D	C7	CA	-0.1882
D	H2	H4	0.2071
D	C2	CA	0.2033
D	C5	CA	-0.0261
D	C8	CT	-0.0271
D	H7	HC	0.0268
D	H8	HC	0.0268
D	C9	C	0.5300
D	O2	O	-0.5744
* E, F, B and D are PNA bases and PNA backbone is denoted by BB.			

Supplementary Table 2: Chemical shifts of the assigned protons (in ppm) for W1-C1 (W1: 5’-CGCGCCTTTT-3’ and C1: 5’-TTTTGGCGCG-3’). All protons were assigned from the COSY and NOESY spectroscopy. (NA-Not Assigned)

Structure/ Nucleobase	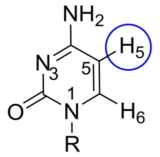	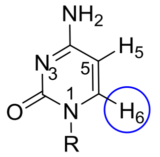	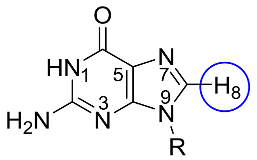	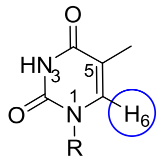	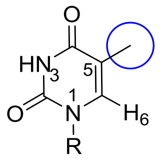	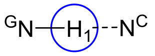	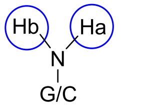	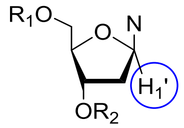
Watson S.								
C1	5.568	7.513					8.481/6.892	6.046
G2			7.759			12.950	6.775(bs)	5.907
C3	5.370	7.308					8.280/6.352	5.895
G4			7.920			12.887	6.945(bs)	5.722
C5	5.414	7.288					8.380/6.506	5.736
C6	5.876	7.593				NA	6.906(bs)	5.880
T				7.504	1.705			6.068
T				7.455	1.689			6.050
T				7.435	1.726			6.060
T				7.352	1.716			5.830
Crick S.								
T				7.565	1.767			6.161
T				7.562	1.767			6.161
T				7.536	1.752			6.117
T				7.540	1.766			6.125
G7			7.900			NA	NA	5.736
G8			7.900			12.950	6.775(bs)	5.736
C9	5.351	7.298					8.214/6.397	5.882
G10			7.864			12.870	6.945(bs)	5.874
C11	5.291	7.280					8.226/6.289	5.874
G12			7.864			13.010	NA	5.721

Supplementary Methods

MD simulations

We built the structure of CEG, GFC, ABT and TDA triads using chimera[7] and optimized them using HF/6-31G* basis set in Gaussian[8]. Using these optimized triads, the helical structure of the DNA-JBγPNA-DNA was constructed using the NAB module of Ambertools[9]. The PNA backbone from X-ray crystal structure, PDB-ID 3PA0[10] (note that the MP-side chain was replaced with methyl group), was grafted onto the DNA-JBγPNA-DNA helix and then energy minimized to obtain the initial structure used for simulations. The structure of JBγPNA-JBγPNA was adopted from an existing NMR structure as mentioned in the manuscript. All the initial structures corresponding to the W-P-C, W1-P1-C1 and P1-P1 complex can be found in the supplemental material files WPC.pdb, W1P1C1.pdb and P1P1.pdb respectively.

MD simulations were performed for W-P-C, W1-P1-C1 and P1-P1 complexes. Each complex was solvated with TIP3P[11] water molecules in a cubic box, and ions were added to maintain the physiological concentration. The systems were energy minimized using the steepest descent method (Press, W. H.; Teukolsky, S. A.; Vetterling, W. T.; Flannery, B. P. Numerical Recipes in FORTRAN; The Art of Scientific Computing, 3rd edition; Cambridge University Press: New York, 1993) and then heated to 300 K under a harmonic restraint of 25 kcal/mol/Å on all the heavy atoms. In a series of six short simulations, the restraint was gradually released. The final unrestrained simulation was performed for 500 ns for each system. Simulations were done in NPT conditions, where the Nose-Hoover thermostat[12,13] was used to maintain the temperature at 300K with a coupling constant of 0.4 ps and the Parinello-Rahman barostat[14] with a coupling constant of 1.0 ps was used to maintain the pressure at 1 bar. A time step of 2 fs was used, and the electrostatic interactions were treated using particle mesh Ewald[15] method with a cutoff at 10 Å. All the simulations were performed using GROMACS-5.1.4[16,17]. The AMBER parmbsc1 force field[18] was used in simulations for both the DNA and PNA. The charges for the non-standard PNA bases and the backbones were derived using RED[19]. The amber atom-types and charges for the E, F, B and D bases and the PNA backbone (BB) used in the simulations are given in Table S1. The bonded and the non-bonded parameters for these residues can be obtained from the parmbsc1 force field in Ambertools[9] using the atom types given below.

Solid-phase synthesis

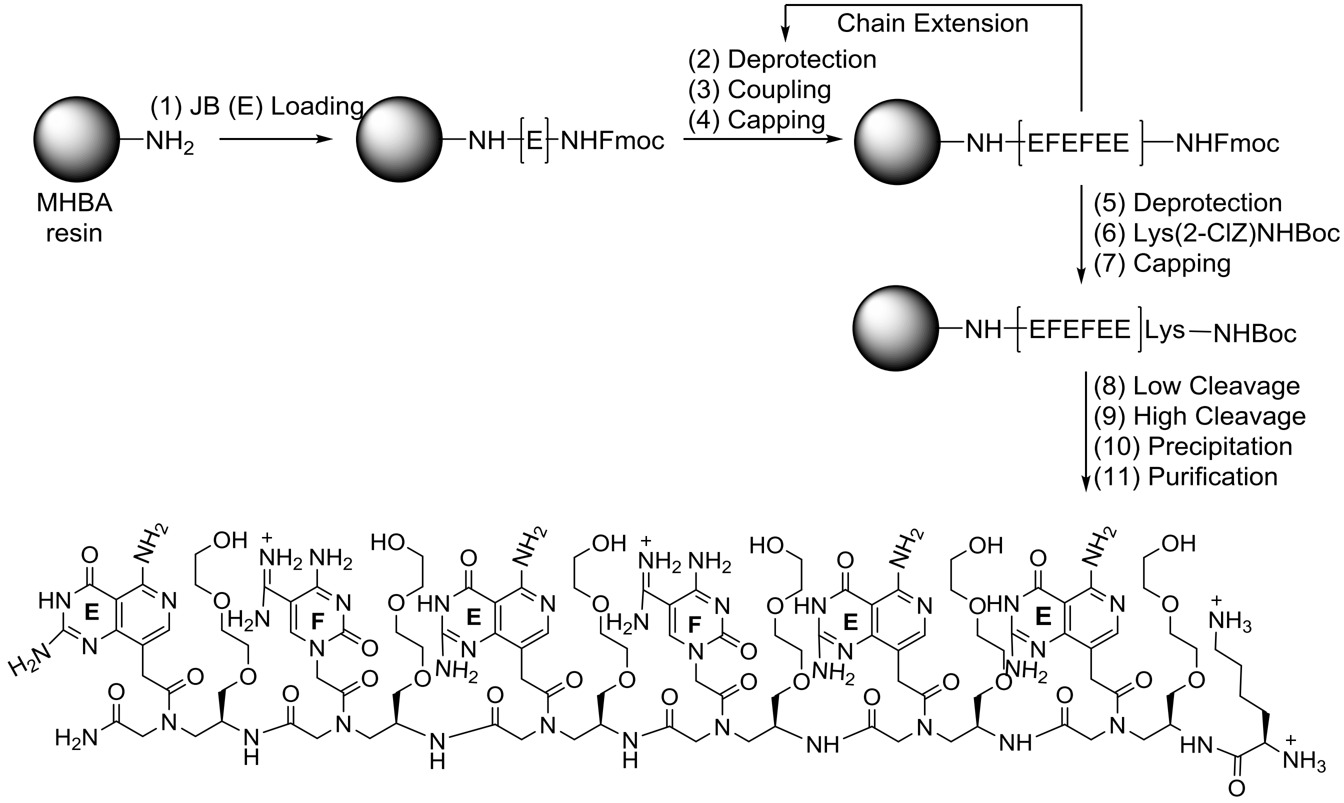

Oligomer Synthesis. The oligomers were synthesized on MBHA resin according to the published protocol with slight Modifications[20]. The resin was washed with 5% DIEA/DCM (2x), pyridine (2X), followed by DMF (4X) and DCM (4X).

Monomer coupling. Upon Kaiser-test confirmation (blue) of the resin, the first coupling solution was prepared by mixing the following materials per 50 mg of resin (amine loading: 0.45mmol/g).

First Coupling solution: The resin was washed with pyridine (1X) before monomer solution was added to the resin. 300 µL of 0.056 M **Monomer solution**, 70 µL of 0.30M **DIEA** solution, and 60 µL of 0.28 M **HBTU** solution were activated for 5 min and added to the resin. The resin was agitated for 8 h before proceeding to the next step.

***Second Coupling solution and later**:* 250 µL of 0.084 M Monomer Solution, 75 µL of 0.30 M DIEA Solution, and 75 µL of 0.28 M HBTU Solution. The mixture was activated for 5 min. The resin was washed with pyridine (1X) before monomer solution was added to the resin. The reaction proceeded for a minimum of 8 h with gentle agitation of the reaction vessel. Completion of the reaction was confirmed by Kaiser-test (pale yellow). The resin was washed with DMF (3X) and DCM (3X).

Capping. The unreacted amines were capped with freshly prepared capping solution. Capping solution (1:25:25, acetic anhydride:NMP:Py; 0.5 mL for 50 mg resin) was added to the resin and agitated the reaction vessel for 4 min. The resin was washed with DMF (4X) and DCM (5X).

Fmoc-deprotection. Fmoc-protecting group was removed by treating resin with a 20% piperidine (0.5 mL for 50 mg resin) solution in DMF (2X) for 7 min each, followed by washes with DMF (3X) and DCM (3X). The Fmoc-deprotection was confirmed by Kaiser-test (blue).

Cleavage. After the final coupling cycle, resin was washed with DMF (5X) and DCM (8X). To the resin, freshly prepared low cleavage solution (*m*-cresol/dimethylsulfide/TFA/TFMSA:100/300/550/50μL) was added, and the reaction vessel was gently agitated for 30 min at room temperature. The low cleavage solution was drained into the centrifuge vial and washed with the TFA solution. The high cleavage solution (*m*-cresol/thioanisole/TFA/TFMSA:150/150/900/300μL) was added to the resin and allowed to agitate for 30 min. The high cleavage solution was then collected in separate centrifuge canonical tube.
Precipitation. To the collected high cleavage solution, cold dry diethyl ether (-60 °C, 14 mL) was added and shaken. The precipitation occurred within 10 min of the time period. The precipitated oligomer was collected by centrifugation and washed with cold diethyl ether (2x) and dried in vacuum.
Purification. The crude oligomers were dissolved in 0.5 mL of 95% water, 5% acetonitrile, and 0.1% of TFA. The crude samples were purified by reverse phase analytical column using acetonitrile and water as a gradient. The resultant oligomer was confirmed by MALDI-TOF spectrometer.

Van't Hoff analysis[21,22]

The thermodynamic parameters were determined from concentration-dependent T_m measurements following Van't Hoff analysis. ΔH° and ΔS° were obtained from a plot of $1/T_m$ (K^{-1}) vs. ln C_T, where C_T is the total strand concentration. ΔH° was obtained from the slope of the plot, which should be a linear function, using the relationship slope = (n–1)R/ΔH, where n is the molecularity of the association interaction (in this case, n = 2) and R is the gas constant (R = 8.314 J/(mol.K)). ΔS° was determined from the y intercept (ln C_T = 0), where for a non-self-complementary oligonucleotide the following relationship stands: slope = [ΔS° - (n - 1)Rln2n/ΔH°]. ΔG° was calculated at 298.15 K using the equation ΔG° = ΔH°-TΔS°.

Supplementary References

1) Scheek, R. M., Boelens, R., Russo, N., van Boom, J. H., Kaptein, R. Sequential Resonance Assignments in NMR Spectra of Oligonucleotides by Two-Dimensional NMR Spectroscopy. *Biochemistry* **23**, 1371-1376 (1984).
2) Hare, D. R., Wemmer, D. E., Chou, S.-H., Drobny, G. *J. Mol. Biol.* **171**, 319-336 (1983).
3) Pandav K., Pandya P., Barthwal R., Kumar S. Structure Determination of DNA Duplexes by NMR. In: Khemani L., Srivastava M., Srivastava S. (eds) *Chemistry of Phytopotentials: Health, Energy and Environmental Perspectives. Springer, Berlin, Heidelberg.*Chapter **33** (2012).
4) Eriksson M.; Nielsen, P. E. Solution structure of a peptide nucleic acid-DNA duplex. *Nat. Struct. Biol*. **3**, 410-413 (1996).
5) He, W. Hatcher, E. Balaeff, A.; Beratan, D. N.; Gil, R. R.; Madrid, M.; Achim, C. Solution Structure of a Peptide Nucleic Acid Duplex from NMR Data: Features and Limitations. *J. Am. Chem. Soc.* **130**, 13264–13273 (2008).
6) Lane, A. N.; Ebel, S.; Brown, T. *Eur. J. Biochem*. **215**, 297-306 (1993).
7) Pettersen, E. F., Goddard,T. D., Huang, C. C., Couch, G. S., Greenblatt, D. M., Meng, E.C. , Ferrin, TE. UCSF Chimera--a visualization system for exploratory research and analysis. *J Comput Chem*. **25**, 1605-1612 (2004).
8) Frisch, M. J., Trucks, G.W., Schlegel, H. B., Scuseria, G. E. Robb, M. A., Cheeseman, J. R., Scalmani, G., Barone, V., Mennucci, B., Petersson, G. A., Nakatsuji, H., Caricato, M., Li, X., Hratchian, H. P., Izmaylov, A. F., Bloino, J., Zheng, G., Sonnenberg, J. L., Hada, M., Ehara, M., Toyota, K., Fukuda, R., Hasegawa, J., Ishida, M., Nakajima, T., Honda, Y., Kitao, O., Nakai, H., Vreven, T., Montgomery Jr., J.A., Peralta, J.E., Ogliaro, F., Bearpark, M.J., Heyd, J., Brothers, E.N., Kudin, K.N., Staroverov, V.N., Kobayashi, R., Normand, J., Raghavachari, K., Rendell, A.P., Burant, J.C., Iyengar, S.S., Tomasi, J., Cossi, M., Rega, N., Millam, N. J., Klene, M., Knox, J. E., Cross, J. B., Bakken, V., Adamo, C., Jaramillo, J., Gomperts, R., Stratmann, R.E., Yazyev, O., Austin, A. J., Cammi, R. Pomelli, C., Ochterski, J. W. Martin, R. L., Morokuma, K., Zakrzewski, V. G., Voth, G. A., Salvador, P., Dannenberg, J. J., Dapprich, S., Daniels, A. D., Farkas, Ö., Foresman, J. B., Ortiz, J. V., Cioslowski, J., Fox D.J. *Gaussian 09*, Gaussian Inc, Wallingford, CT, USA, (2009)
9) Macke, T. J., Case, D. A. Modeling Unusual Nucleic Acid Structures. *Molecular Modeling of Nucleic Acids,* **24**, 379-393 (1997).
10) Yeh, J. I., Shivachev, B., Rapireddy, S., Crawford, M. J., Gil, R. R., Du, S., Madrid, M., and Ly, D. H. Crystal Structure of Chiral γ PNA with Complementary DNA Strand—Insights into the Stability and Specificity of Recognition and Conformational Preorganization. *J. Am. Chem. Soc.,* **132**, 10717−10727 (2010).
11) Jorgensen, W. L., Chandrasekhar, J., Madura, J. D., Impey, R. W., Klein, M. L. Comparison of simple potential functions for simulating liquid water. *J. Chem. Phys*. **79**, 926-935 (1983).
12) Nose, S. A molecular dynamics method for simulations in the canonical ensemble. *Mol. Phys.* **52**, 255−268 (1984).
13) Hoover, W. G. Canonical dynamics: Equilibrium phase-space distributions. *Phys. Rev. A.* **31**, 1695−1695 (1985).
14) Parrinello, M., Rahman, A. Polymorphic transitions in single crystals: A new molecular dynamics method. *J. Appl. Phys.* **52**, 7182−7190 (1981).
15) Darden, T., York, D., Pedersen, L. Particle mesh Ewald: An N·log(N) method for Ewald sums in large systems. *J. Chem. Phys*. **98**, 10089-10092 (1993).
16) Hess, B., Kutzner, C., van der Spoel, D., Lindahl, E. GROMACS 4: Algorithms for highly efficient, load-balanced, and scalable molecular simulation. *J. Chem. Theory Comp.***41**, 435–447 (2008).
17) Dupradeau, F.-Y., Pigache, A., Zaffran, T., Savineau, C., Lelong, R., Grivel, N., Lelong, D., Rosanski, W., Cieplak, P. The R.E.D. tools: Advances in RESP and ESP charge derivation and

force field library building, The R.E.D. tools: advances in RESP and ESP charge derivation and force field library building. *Phys. Chem. Chem. Phys.* **12**, 7821-7839 (2010).
18) Abraham, M.J., Murtola, T., Schulz, R., Pall, S., Smith, J.C., Hess, B., Lindah, E. GROMACS: High performance molecular simulations through multi-level parallelism from laptops to supercomputers. *SoftwareX*, **1–2**, 19–25 (2015).
19) Ivani, I., Dans, P.D., Noy, A., Perez, A.; Faustino, I., Hospital, A., Walther, J., Andrio, P., Goni, R., Balaceanu, A., *et al*. Parmbsc1: a refined force field for DNA simulations. *Nat. Methods*, **13**, 55–58 (2016).
20) Christensen, L., Fitzpatrick, R., Gildea, B., Petersen, K. H., Hansen, H. F., Koch, T., Egholm, M., Buchardt, O., Nielsen, P. E., Coull, J., Berg, R. H. Solid-phase synthesis of peptide nucleic acids. *J. Pept. Sci*.**1**, 175–183 (1995).
21) Marky, L., Breslauer, K. J. Calculating thermodynamic data for transitions of any molecularity from equilibrium melting curves. *Biopolymers* **26**, 1601–1620 (1987).
22) Sahu, B., Sacui, I., Rapireddy, S., Zanotti, K. J., Bahal, R., Armitage, B. A., Ly, D. H. Synthesis and Characterization of Conformationally Preorganized, (R)-Diethylene Glycol-Containing γ-Peptide Nucleic Acids with Superior Hybridization Properties and Water Solubility. *J. Org. Chem.* **76**, 5614–5627 (2011).
